# Linear morphometrics fail to support strong sexual dimorphism in *Uintatherium anceps*

**DOI:** 10.1371/journal.pone.0345236

**Published:** 2026-10-07

**Authors:** Kevin D. Mulcahy

**Affiliations:** 1 Biodiversity Institute and Natural History Museum, University of Kansas, Lawrence, Kansas, United States of America; 2 Department of Ecology and Evolutionary Biology, University of Kansas, Lawrence, Kansas, United States of America; Liverpool John Moores University, UNITED KINGDOM OF GREAT BRITAIN AND NORTHERN IRELAND

## Abstract

Uintatheres, mammals belonging to the extinct order Dinocerata, are among the most recognizable of all Paleogene (~66–23 Ma) organisms. Unmistakable for their bizarre skulls with multiple pairs of horns and saber-like upper canines, uintatheres have captivated paleontologists since the late nineteenth century. Since their initial discovery, uintatheres have been regarded as a classic example of dramatic sexual dimorphism in the fossil record, with males purported to be larger and possess more prominent horns and canines than females. However, the hypothesis that uintatheres were highly sexually dimorphic has never been formally tested. Here, traditional, linear morphometrics are implemented with a collection including most known skulls of *Uintatherium anceps* to quantify patterns of cranial variation within this taxon. Use of univariate and multivariate statistical approaches provides some evidence of dimorphism in *Uintatherium*, particularly in the length of the canines, but the expected signals of strong sexual dimorphism are generally lacking. To verify the approaches used, the same suite of analyses was performed on a similarly sized dataset from *Bison bison*. Statistical signals of sexual dimorphism are clearer in this extant taxon. Comparison with other extant mammalian herbivores reveals that the estimated level of sexual dimorphism in skull length for *Uintatherium* is not especially high. In light of these findings, as well as the current understanding of uintathere systematics and paleoecology, it is argued that extreme sexual dimorphism should not be treated as the null hypothesis for this clade. These results may point to a broader pattern in mammalian evolution in which traits like horns and large canines emerge in both males and females of a species despite being primarily selected for in one sex.

## Introduction

Sexual dimorphism is the phenomenon in which individuals of different sexes of the same species differ in their morphology beyond primary sexual characteristics [1–5]. Naturalists have observed patterns of sexual dimorphism for centuries, and description of dimorphism and its causes factored heavily into much of Darwin’s work on sexual selection [1,2]. Among mammals, researchers historically accepted that males tended to be larger and possess more elaborate secondary sexual characteristics than females [1,2,6–10]. This notion was explained by higher investment in reproduction by females, and therefore higher selective pressures among males, especially in polygynous mating systems [2,6,10–13]. In such systems, it has been posited that both male-male competition and female choice for the most suitable mates lead to heightened sexual selection among males, resulting in the development of larger body sizes and/or prominent display characteristics [2,6,14–16]. Indeed, in many of the most famous and well-studied extant mammals, males are significantly larger than females and possess obvious secondary sexual characters (e.g., [2,17–19]).

However, while sexual dimorphism has been conclusively documented in many taxa, advances in mammalian research have demonstrated that patterns of dimorphism are not ubiquitous or uniform among mammals. In the 1970s, Ralls reviewed rates of sexual size dimorphism across mammalian clades and found that species with little dimorphism or larger females were surprisingly numerous [20,21]. In 2024, Tombak and colleagues surveyed extant placental mammals and found that males are not in fact measurably larger than females in most species [10]. Sexual dimorphism has also been shown to carry phylogenetic signal, with rates of dimorphism differing between mammalian orders [10,22]. For instance, while species of artiodactyls, carnivores, and primates almost universally exhibit larger males than females, monomorphism and larger females are common among rodents and bats [10,11,23–27]. Other clades of large mammals, including perissodactyls, exhibit only minimal to moderate degrees of sexual dimorphism in body size and the prominence of traits like enlarged canines and horns [7,28–33]. It has therefore become increasingly unclear whether classical larger-male patterns of sexual dimorphism were ancestral among most mammals.

Among fossil vertebrates, sexual dimorphism has a rich history of documentation and research. Especially in the field of paleoanthropology, the study of sexual dimorphism in the fossil record has formed a key component of research into sexual selection and the evolution of social behavior more broadly [23,34–36]. Instances of sexual dimorphism have been reported among extinct members of virtually all major vertebrate clades, including mammals, birds, and non-avian dinosaurs (e.g., [37–48]). However, researchers have also raised caution in attributing patterns of morphological variation among extinct taxa to sexual dimorphism [49–52]. In extant animals, it is relatively easy to test for and quantify patterns of sexual dimorphism, as males and females can usually be observationally or experimentally distinguished [53,54]. In contrast, even in truly dimorphic taxa, fossilized remains rarely preserve the primary sexual characters necessary to determine the sex of an individual [55,56]. This lack of a priori knowledge of individual sex precludes the use of simple statistical tests to quantify patterns of dimorphism, as patterns of morphological variation could instead represent ontogeny, taxonomic differences, phenotypic plasticity, or random individual variation [49,52,57–59]. Additional factors such as small sample sizes and potentially biased sex ratios present further challenges for the study of sexual dimorphism in the fossil record [49,52,56,60].

Still, approaches to detect and quantify patterns of sexual dimorphism in the fossil record have been developed and applied. One relatively intuitive approach to detect patterns of dimorphism is to test the distribution of a feature of interest for statistically significant deviation from normality or unimodality [52,61,62]. Other methods, including mixture modeling, variance based analyses, principal component analysis (PCA), and cluster analysis have been advocated as potentially more robust approaches to detect underlying patterns of dimorphism among traits of interest in the fossil record [27,52,62–71]. Still other novel frameworks to detect sexual dimorphism in the fossil record continue to be developed [72].

One group of fossil animals for which sexual dimorphism has been historically accepted are the uintatheres, extinct mammals belonging to the order Dinocerata [73–78]. Uintatheres ranged from the late Paleocene to the middle Eocene (~60−40 Ma) in Asia and North America, and were graviportal, vaguely ungulate-like eutherians, representing the first truly massive land mammals, with body masses potentially exceeding 4,500 kg [73,75–77,79,80]. Uintatheres, especially the derived uintatheriines, are easily recognized by their bizarre skulls, with later forms possessing three pairs of cranial protuberances and massive, saber-like canines (Fig 1) [73,76,78]. Likely due to their massive body size and enigmatic appearance, uintatheres have figured heavily in the history of paleontology, serving as key taxa in the infamous “Bone Wars” of Marsh and Cope [73,81,82]. Despite such a colorful history of research, the broader affinities of uintatheres remain unresolved, with various hypotheses aligning them with Laurasiatheria, Euarchontoglires, or Afrotheria [76,81,83]. Alternatively, uintatheres have been united with other extinct and early diverging mammalian clades such as Pantodonta, suggesting a potential phylogenetic position near the base of Placentalia [73,84,85].

**Fig 1:**
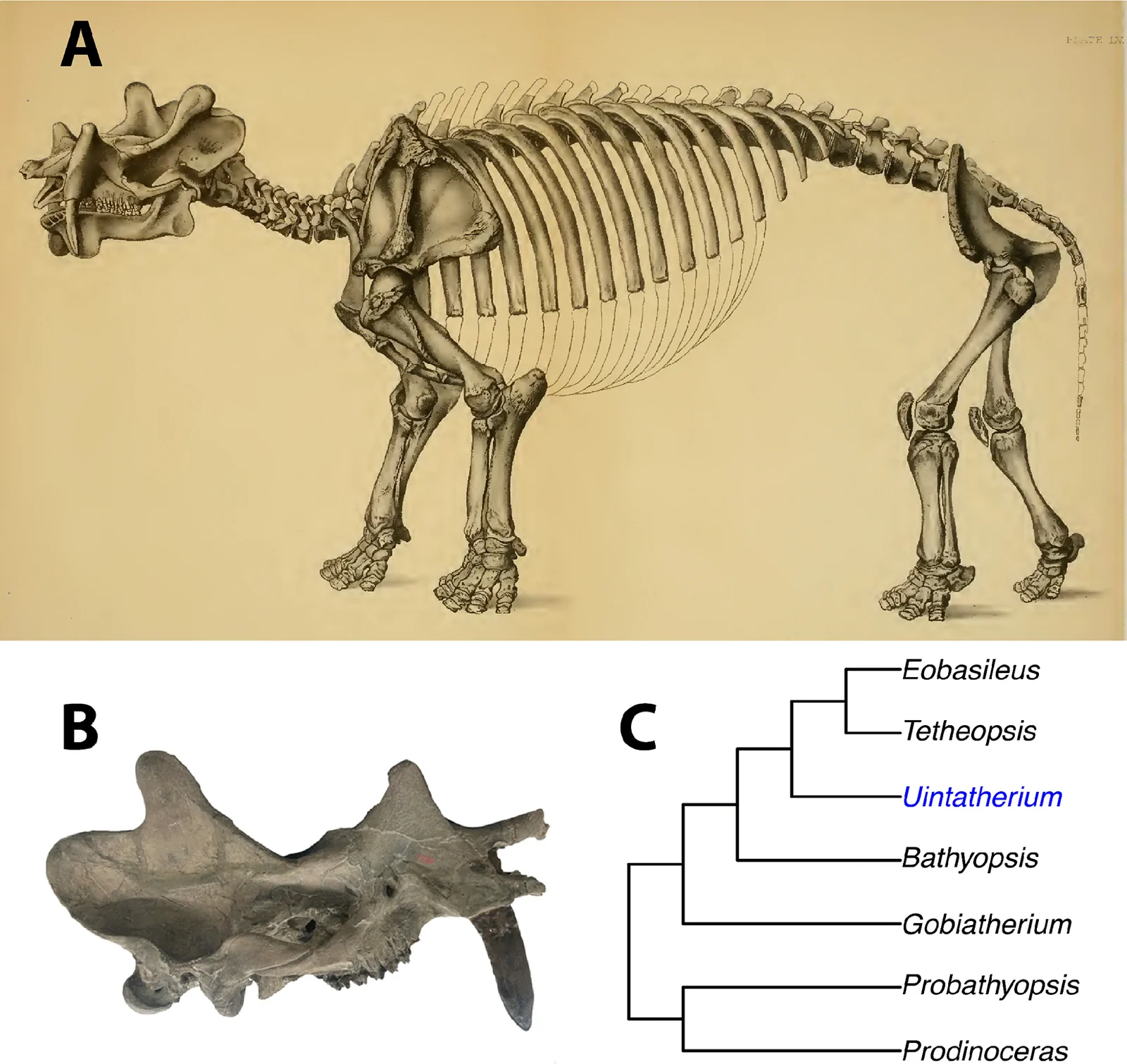
Osteology and systematics of Dinocerata. (**A**) Plate LV from Marsh [73] showing a restoration of *Uintatherium anceps* (Marsh’s holotype of *Dinoceras laticeps*, YPM VP 011036). Reprinted from Marsh [73] under a CC BY license, with permission of University of Connecticut Libraries, original copyright 1886. (**B**) Skull of *Uintatherium anceps* (YPM VP 011036). (**C**) Proposed phylogenetic relationships within Dinocerata, highlighting the position of *Uintatherium*. Adapted and modified from Schoch and Lucas [76]. **Abbreviations**: YPM VP, Vertebrate Paleontology Collections at the Yale Peabody Museum of Natural History.

In 1886, Marsh first posited that uintatheres were highly dimorphic, and this is one of the earliest reports of sexual dimorphism in the entire history of vertebrate paleontology [73]. Subsequent authors have expanded on Marsh’s initial claims and attributed the wide range of cranial variation observed within uintathere species to significant differences between sexes (Fig 2) [75,76,78]. Traditionally, large uintathere specimens with prominent horns and canines have been interpreted as male, while smaller individuals with less dramatic craniodental features have been identified as females [73,75,76,78,86]. However, to date, the hypothesis that the skulls of uintatheres display a high degree of sexual dimorphism has not been adequately tested or quantitatively validated. Furthermore, while previous authors have agreed that uintatheres were strongly dimorphic, they have differed in their interpretation as to how the sexes actually varied. For instance, Marsh claims that females of *U. anceps* lacked the prominent inframandibular flange seen in males, while Wheeler argues for the presence of fully developed flanges in individuals of both sexes [73,75].

**Fig 2:**
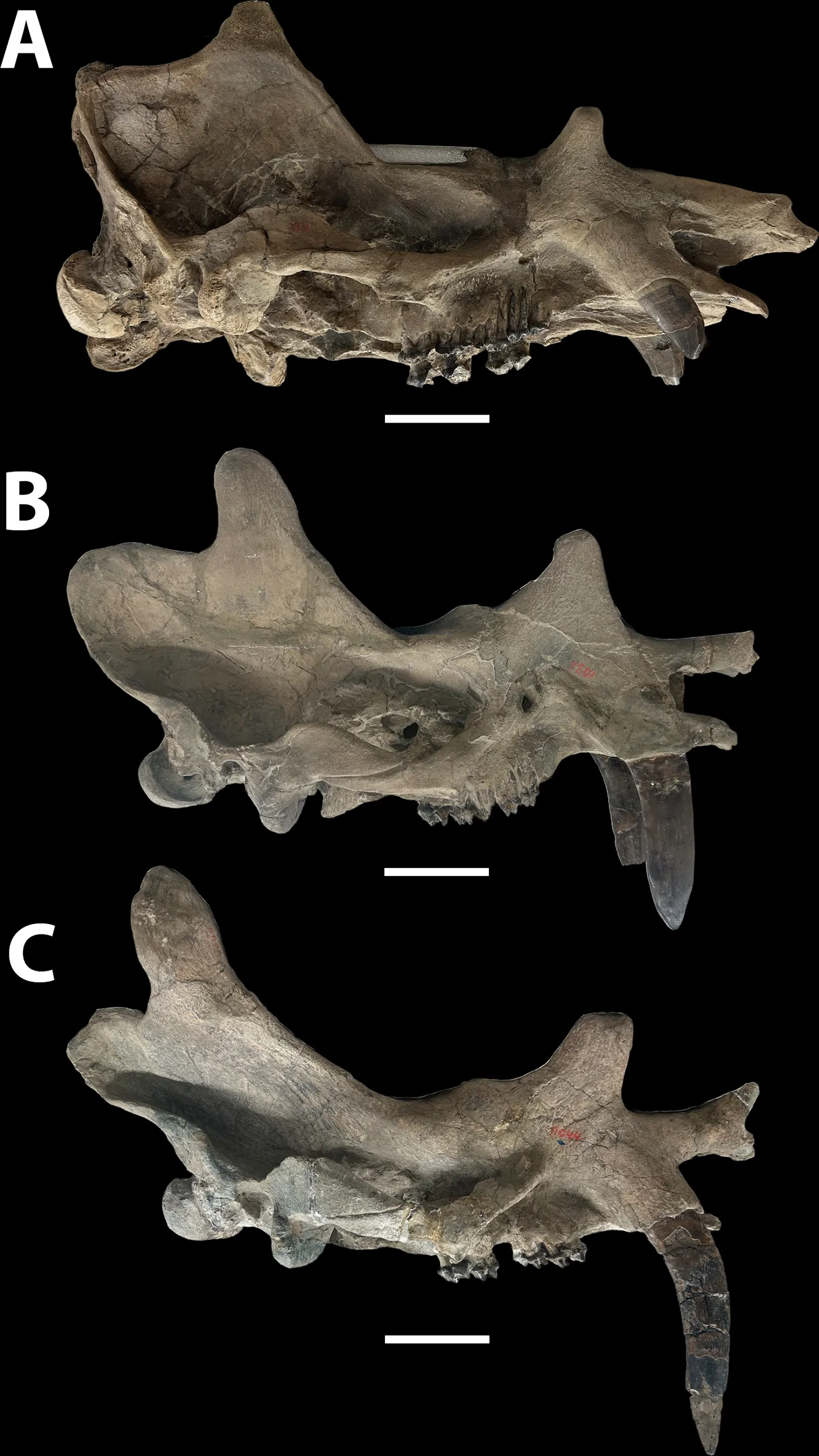
Cranial variation in the skulls of *Uintatherium anceps.* (**A**) AMNH FM 1671, purported female according to Wheeler [75] and Turnbull [78]. (**B**) YPM VP 011036, sex unreported by previous authors. (**C**) YPM VP 011044, purported male according to Marsh [73]. Scale bars equal 10 cm. **Abbreviations**: AMNH FM, Fossil Mammal Collections at the American Museum of Natural History; YPM VP, Vertebrate Paleontology Collections at the Yale Peabody Museum of Natural History.

Given their early divergence and potentially basal phylogenetic position, determining whether uintatheres were sexually dimorphic could yield insights into the character polarity of sexual dimorphism among placental mammals more broadly. Here, a quantitative morphometric analysis of cranial morphology in *Uintatherium anceps* is presented. The data gathered in this study is used to test the hypothesis that this taxon was sexually dimorphic. As previous authors have expressed skepticism towards traditional approaches used to detect patterns of sexual dimorphism in fossil taxa, a variety of methods including classical tests of univariate normality and unimodality, principal component analysis, cluster analysis, and variance-based estimations of sexual dimorphism were employed [27,49,52,58,71]. To validate the approaches used, a novel dataset from skulls of the American bison was collected to serve as an extant analogue. The American bison is a fitting analogue for *Uintatherium* due to its similar body size and the fact that it exhibits patterns of sexual dimorphism similar to those which have been proposed for dinoceratans [87–90]. Lastly, the estimated degree of dimorphism for cranial length in *Uintatherium* is compared to an assortment of extant mammalian herbivores. The following represents a robust analysis of purported patterns of sexual dimorphism in the fossil record which can serve as a reference for students of other taxa.

## Materials and methods

### Collections and permissions

Specimens referenced in this study are curated in the following collections: **AMNH FM**, Fossil Mammal Collections at the American Museum of Natural History, New York, New York, USA; **YPM VP**, Vertebrate Paleontology Collections at the Yale Peabody Museum of Natural History, New Haven, Connecticut, USA; **YPM VPPU**, Princeton University Vertebrate Paleontology Collections acquired by the Yale Peabody Museum of Natural History, New Haven, Connecticut, USA; **KU:Mamm**, mammalogy collections at the University of Kansas Natural History Museum, Lawrence, Kansas, USA. All necessary permissions were obtained for the described study, which complied with all relevant regulations.

### Specimens and cranial measurements

For this study, 26 complete and partial skulls of mature individuals (possessing full adult dentition) of *Uintatherium anceps* at the American Museum of Natural History and the Yale Peabody Museum of Natural History were analyzed. *U. anceps* was chosen as the focal taxon as it is by far the best represented uintatheriine. Together, this dataset encompasses most *U. anceps* crania known. All specimens come from roughly coeval Bridgerian-aged exposures in the Bridger and Washakie Basins of southwestern Wyoming (S2 File) [73,75,78,84,91–95]. As such, observed morphological disparity is unlikely to reflect spatial or temporal differences. When assessing evidence of sexual dimorphism, certain cranial features were of particular interest, including the anteriorly projecting premaxillaries and nasals (and prenasal bones on some specimens), the three pairs of horns or cranial protuberances (nasal, maxillary, and parietal), the posteriorly raised occipital crest, and the saber-like canines (Fig 3A).

**Fig 3:**
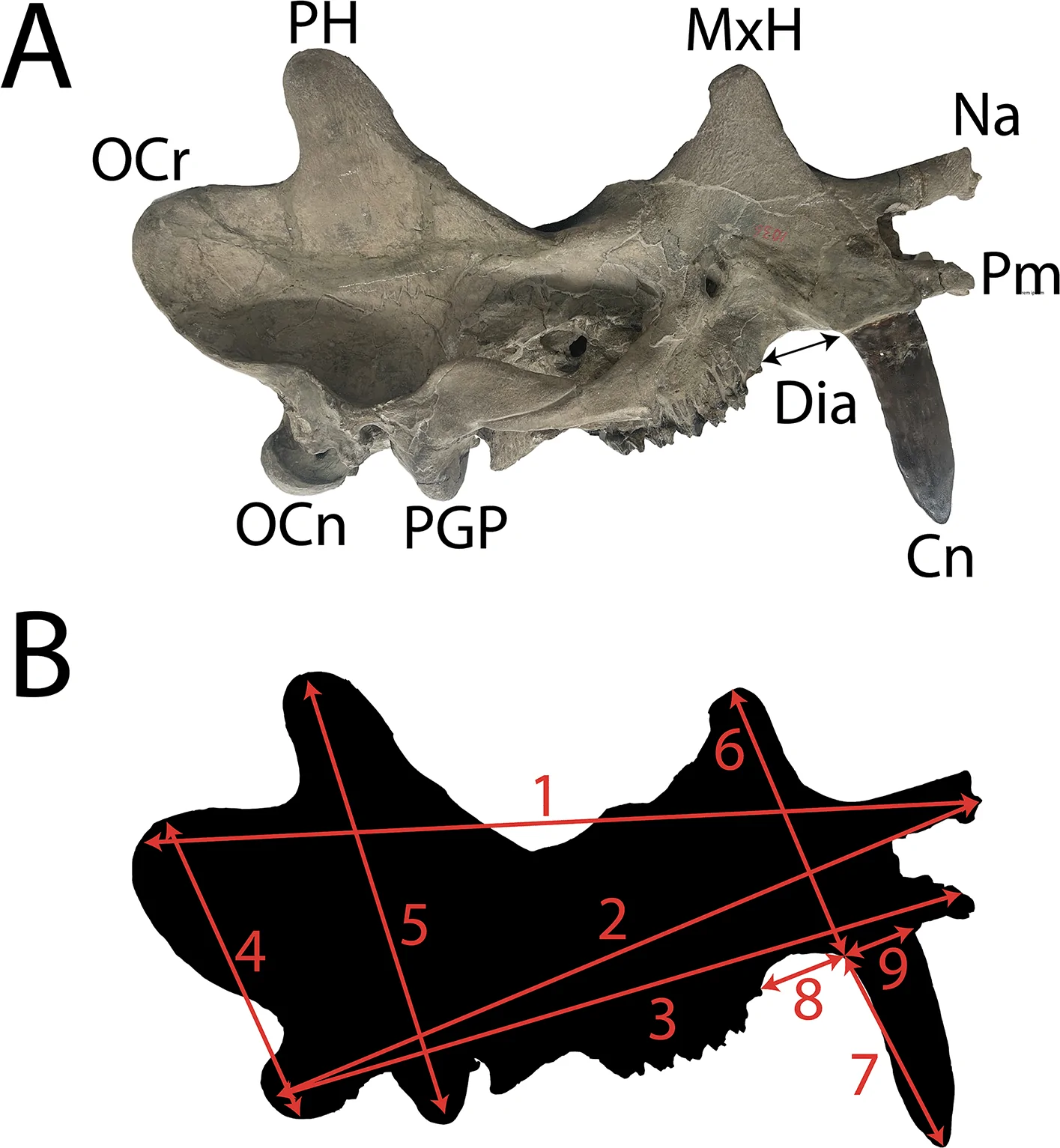
Cranial features and measurements referenced in study of *Uintatherium anceps.* (**A**) Relevant anatomical features on the skull of *Uintatherium anceps* (YPM VP 011036): OCr, Occipital crest; OCn, Occipital condyle; PGP, Postglenoid process; PH, Parietal horn/protuberance; MxH, Maxillary horn/protuberance; Na, Anterior extent of nasals; Pm, anterior extent of premaxillaries; Cn, Canine; Dia, Diastema. (**B**) Linear cranial metrics used in analyses: 1, Length of skull from nasals to occipital crest (nasOcc); 2, Length of skull from nasals to occipital condyles (nasCon); 3, Length of skull from premaxillaries to occipital condyles (preCon); 4, Height of skull from occipital condyle to occipital crest (occCon); 5, Height of parietal protuberance (parPro); 6, Height of maxillary protuberance (maxAlv); 7, Length of canine (alvCan); 8, Diastemal length (diaLen); 9, Width of canine at base (canWid). **Abbreviations**: YPM VP, Vertebrate Paleontology Collections at the Yale Peabody Museum of Natural History.

The large size of these skulls has precluded computed tomography (CT) scanning and three-dimensional geometric morphometric analyses. Due to their tremendous mass, age (both geological and historical), and fragile reconstruction with plaster, physical manipulation of the uintathere skulls was minimized. Specimens in the AMNH FM and YPM VP are firmly affixed to plaster or wooden bases, which allow the fossils to be easily moved between shelves but largely prohibit any rotation or free motion of the skull itself. As some skulls are oriented with the dorsal side facing upwards and others are oriented with the ventral side up, a traditional morphometric approach, through which patterns of morphological variation were captured by linear measurements of the skull that could be observed in the parasagittal plane, was employed.

Data used in analyses was gathered by taking images of the skulls in lateral view with a scale bar and measuring the distance between landmarks of interest in the software ImageJ. Measurements were also gathered manually using a tape measure, and sensitivity analyses were performed to quantify potential sources of error from the measurement techniques used (S3 Table, S1–S7 Figs, and S2, S3, S6, S7 Files). Measurements obtained in ImageJ were preferred to those gathered by hand, as the large size, varying degrees of preservation, and inconsistent skull orientation precluded consistency in manual measurements across specimens. Bland-Altman plots and linear models were generated to confirm that the difference between the by-hand and ImageJ values did not covary with the size of the skull (S1–S4 Figs, S3 Table, and S3, S6 Files). Results were non-significant in all tests in *Uintatherium*, indicating that measurement error did not increase with specimen size (S3 Table). Simulations were also performed to quantify the potential effects of photographic parallax, lens distortion, and variable scale bar placement (S5–S6 Figs and S7 File). In these simulations, when angle, zoom, and scale bar depth were held constant, measurements varied by no more than 13.5% across replicates (S7 File). To minimize these effects, all images used in this study were taken at an orthogonal angle to the specimen using the same camera, and the scale bar was placed at a consistent depth relative to the skulls.

Nine cranial metrics were chosen to be measured for each skull, capturing both the overall size of the specimen as well as the relative development of the purportedly dimorphic characters (Fig 3B and S1 Table). While this linear morphometric approach likely limits statistical power in comparison to two- or three-dimensional geometric morphometrics, it allows for inclusion of partial and damaged skulls which lack certain anatomical landmarks in the analysis. Linear morphometrics can be easily applied in analyses of purported patterns of dimorphism in other clades with similarly problematic fossil records, promoting broad reproducibility.

Most of these specimens endured some degree of damage or distortion through various taphonomic processes. In cases where features of interest were present but too damaged or distorted to be reliably measured, measurements were not included. Uintathere skulls exhibit bilateral symmetry, allowing for some or all cranial metrics to be measured on at least one side of the skull in most cases. Still, taphonomic distortion introduces analytical limitations into this study. For instance, after removal of highly damaged specimens, canine length could be measured in only ten of 26 specimens, limiting the power to detect dimorphism in this character. Such limitations underscore the need for multiple techniques in assessing sexual dimorphism in the fossil record, as different methods are more or less susceptible to type II errors under small sample sizes [52,71,72].

In-vivo wear and damage may further obscure signals of sexual dimorphism in traits like canine length. While it might be inferred that males and females wore their canines at similar rates if this damage was incurred through feeding, small sample size precludes any test of this assumption. Further, a number of the skulls included in this study have been partially reconstructed with plaster by previous researchers. The reconstructed portions of these skulls were often sculpted and painted to match the texture and color of the surrounding bone, making it difficult to discern the margins of the actual fossil material. The potential impacts of these reconstruction techniques on the analyses performed are addressed in the ‘Discussion’ section.

### Traditional approaches to detect sexual dimorphism

To assess evidence of sexual dimorphism, a variety of statistical approaches were employed using R statistical software. First, simple histograms and violin plots were generated to visualize the distribution of each cranial metric, using the plotting tools available in base R and ‘ggplot2’ [96,97]. Shapiro-Wilk tests of normality and Hartigan’s dip tests of unimodality were performed for each metric using base R and the package ‘diptest’ [98]. In each of these statistical tests, the null hypothesis is that the sample of cranial metrics was drawn from a normally or unimodally distributed population (i.e., one which exhibits sexual monomorphism). Dip test p-values were computed through Monte Carlo simulation with 2,000 replicates. Mann-Whitney U tests were also performed to determine if purported male and female specimens differed in their values for each metric.

To further assess evidence of multimodality, univariate mixture modeling was employed using the package ‘mclust’ to determine how many underlying normal distributions (Gaussians) best fit the data structure for each cranial metric [99]. For each metric, the model with the optimal number of clusters was identified based on the Bayesian Information Criterion (BIC) [100]. In principle, if *U. anceps* were highly sexually dimorphic, each metric exhibiting dimorphism should be best fit by a model with two components, one for males and one for females.

While statistically significant deviation from a normal, unimodal distribution in any one metric may provide evidence of sexual dimorphism, non-significant test results do not preclude it, nor do significant results confirm its presence. Therefore, several multivariate approaches were employed. As these analyses cannot be performed where values for individual metrics are missing, imputation was necessary for skulls that were partially broken. Where appropriate (three or fewer values missing for a specimen), imputation was performed with a Principal Components Analysis model using the R package ‘missMDA’ [101]. Particularly fragmentary specimens (more than three missing values) were not considered in multivariate analyses. Before clustering, all values were standardized so that each cranial metric was normalized and weighted equally in multivariate analyses.

Principal component analysis (PCA) is one method that can be used to visualize the distribution of specimens within a multidimensional morphospace. PCA is a common ordination technique by which multiple dimensions of variation are reduced into a small number of orthogonal principal components (PCs) capturing covariation across multiple metrics [102]. These principal components can be analyzed for their eigenvalues (how much of the overall variation in the dataset is captured by each principal component) and loadings (how much each variable contributes to each principal component). PCA was performed using the R package ‘FactoMineR’ and results were visualized in a bivariate plot displaying scores for PC1 and PC2 [103]. If uintatheres differ consistently in their morphology based on sex, one would expect purported males and females to occupy distinct morphospaces in PCA. PCA was also used to determine if purported male and female specimens showed statistically significant differences. A second PCA was performed, this time only including specimens for which sex has been postulated by previous authors [73,75,78]. Permutational multivariate analysis of variance (PERMANOVA) was performed using the R package ‘vegan’ to determine if significant PCs (accounting for the first 95% of the cumulative variance) differed between purported females and males [104].

Another multivariate method which can be used to explore purported patterns of sexual dimorphism is k-means cluster analysis. k-means clustering is partitional, meaning the user specifies how many discrete clusters the data should form [105]. If, as would be expected in a highly dimorphic taxon, male and female uintatheres displayed consistent differences in their overall morphology, splitting the dataset into the two most favorable clusters might indicate which specimens were males and which were females. k-means clustering was therefore performed to partition the data into two clusters using the ‘stats’ package and results were superimposed onto the bivariate plot generated through PCA. This allowed for direct comparison between the morphospaces of the two most favorable clusters and those occupied by purported males and females. Total within sum of squares, average silhouette width, and gap statistics were also used to determine the optimal number of clusters formed by the data using the ‘factoextra’ package [106].

Hierarchical clustering, by which skulls are clustered and arranged in a dendrogram based on similarities and differences across cranial metrics, was also performed using the R packages ‘ComplexHeatMap’ and ‘pvclust’ [107–109]. In hierarchical clustering, specimens that share similar scores across all cranial metrics are placed near one another in the resulting dendrogram. Similar in principle to PCA, if males and females differ consistently in their morphology, one would expect the purported members of each sex to cluster together on the dendrogram.

### Extant analogy and variance-based estimations of dimorphism in *Uintatherium*

To contextualize results and validate the approaches employed, all analyses were also performed in the extant American bison (*Bison bison*). *B. bison* was chosen as an extant analogue because of its large size, herbivorous feeding adaptations, and overall pattern of sexual dimorphism including moderate dimorphism in body size and strong dimorphism in the prominence of cranial horns [87,89,90]. Bison skulls retain a bony horn core or cornual process, providing an osteological correlate analogous to the horns of uintatheres [87,88]. Additionally, as *B. bison* was historically abundant across the central United States, the species is represented by extensive museum collections, with enough skulls to facilitate analyses of dimorphism. For this study, cranial metrics were gathered from 23 skulls of *B. bison* at KU:Mamm. Of these, nine were female, seven were male, and seven lacked associated sex metadata (specimen metadata can be found on the open-access KU:Mamm Specify database).

Seven cranial metrics were chosen in *B. bison*, capturing both the overall size of the skull and the relative prominence of the horns (Fig 4 and S2 Table). As in *Uintatherium*, all chosen measurements could be observed in the same anatomical plane. However, as *Bison* only possess one pair of horns which project laterally, specimens were photographed and measured in the frontal plane. Of the seven measurements chosen in *Bison*, some (e.g., length/width of cornual process) should be highly dimorphic, while others (e.g., length of the skull from prosthion to nasion) should exhibit relatively little dimorphism. Therefore, this analogy allows for the contextualization of the results obtained for each metric in *Uintatherium* among a suite of characters displaying a range of dimorphism in an extant taxon. Univariate and multivariate analyses were performed following the same procedures used in *Uintatherium*. For Mann-Whitney U tests and PERMANOVA, only eight bison specimens (four female and four male) were included to maintain equal sample size with the uintatheres whose sex has been previously inferred.

**Fig 4:**
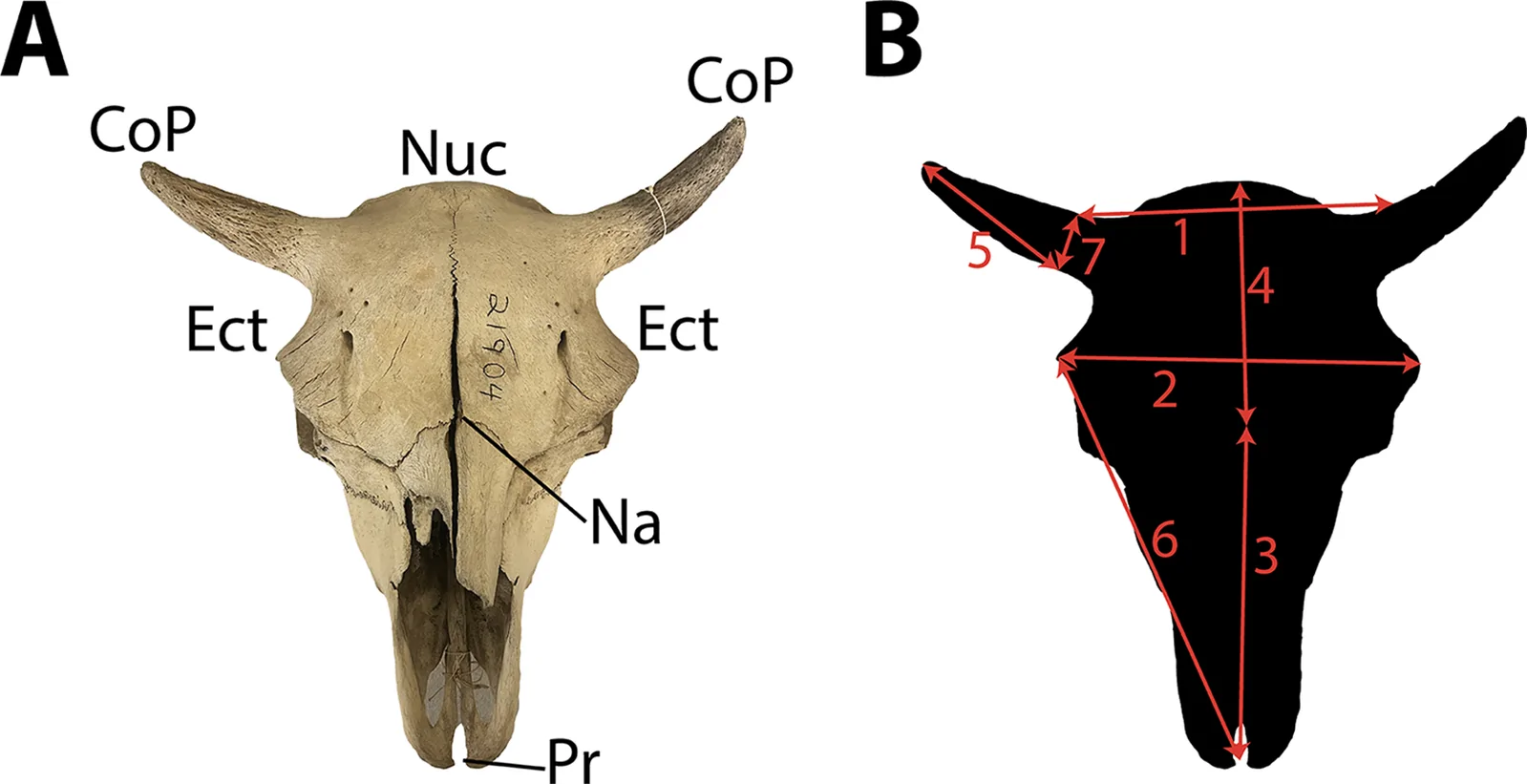
Cranial features and measurements referenced in study of *Bison bison.* (**A**) Relevant anatomical features on the skull of *Bison bison* (KU:Mamm 21904): CoP, Cornual process (frontal horn); Ect, Ectorbitale; Pr, Prosthion; Na, Nasion; Nus, Nuchal line. (**B**) Linear cranial measurements used in analyses: 1, Width of skull between bases of cornual processes (basFro); 2, Width of skull between left and right ectorbitale (ectEct); 3, Length from prosthion to nasion (proNas); 4, Length from nasion to nuchal line (nasNuc); 5, Length of cornual process from base to tip (basTip); 6, Distance between prosthion and ectorbitale (ectPro); 7, Width of cornual process at base (basWid). **Abbreviations**: KU:Mamm, mammalogy collections at the University of Kansas Natural History Museum.

Lack of homology between the characters chosen in *Uintatherium* and *Bison* precludes direct comparison of the results of multivariate analyses. However, as the traits selected in *Bison* display a similar range of sexual dimorphism as has been purported for the chosen characters in *Uintatherium*, multivariate analogy can indicate the signals of dimorphism expected to be recovered from analysis of these characters. Skull length was also measured in both species allowing for some direct comparison with *Uintatherium*. To verify that skull length as measured in the frontal plane was directly comparable to skull length in the sagittal plane, a linear model was generated using 3D scans of *Bison* skulls from Morphosource for which both measurements could be obtained. Skull length measured in the frontal plane was found to be a highly significant predictor of skull length in the sagittal plane (p = 0.011, R^2 = 0.9148), and the two values differed by no more than 2.16% for any specimen (S7 Fig and S6 File).

Lastly, the level of sexual dimorphism was estimated for each trait in *Uintatherium* using variance-based methods presented in Van Valkenburg and Sacco (2002) [27,71]. Sexual dimorphism indices (SDI – the ratio of the male to female mean for a trait) for each cranial metric in *Uintatherium* and *Bison* were generated using the means method (MM) and finite mixture analysis (FMA). These two methods yield estimates of the maximum amount of sexual dimorphism that could be represented in a sample based on the observed variance. In MM, the sample is simply split into two subsamples about the mean, and the ratio between these two subsample means is then calculated. In FMA, a sample is split into the two most variable subsamples that could be contained within a single unimodal distribution, and then, as in MM, SDI is estimated as the ratio of these two subsample means. Both MM and FMA are prone to overestimating dimorphism, so the estimates obtained can be treated as upper bounds on the degree of sexual dimorphism expressed by *U. anceps* for each trait of interest [71]. Estimates for sexual dimorphism in skull length, the only metric that was directly comparable between *Uintatherium* and *Bison*, were also compared to patterns seen among a variety of extant mammalian herbivores using data obtained from the literature [7,29,31,89,110–126].

## Results

### Univariate statistical tests

In *U. anceps*, one of the nine cranial metric distributions deviated significantly (p ≤ 0.05) from normality, and no metrics showed a significantly non-unimodal distribution (Table 1 and S8 Fig). Four metrics of interest in *B. bison* display distributions that differ significantly from normality (Table 1 and S9 Fig). Only one Mann-Whitney U test (parPro) demonstrated statistically significant difference between purported female and male uintatheres, while six of seven tests indicated significant differences between female and male bison (p ≤ 0.05) (S4 and S5 Files).

**Table 1:** Results of Shapiro-Wilk and Hartigan’s dip tests in *Uintatherium anceps* and *Bison bison.*

Genus	Measurement	n	W Stat	p	Dip Stat	p
***Uintatherium***	**nasOcc**	25	0.965	0.540	0.042	0.991
	**nasCon**	19	0.978	0.911	0.052	0.968
	**preCon**	14	0.934	0.343	0.081	0.617
	**occCon**	19	0.973	0.836	0.077	0.457
	**parPro**	19	0.938	0.241	0.079	0.408
	**maxAlv**	21	0.972	0.775	0.072	0.490
	**alvCan**	10	0.832	0.035*	0.131	0.094
	**diaLen**	29	0.952	0.426	0.076	0.482
	**canWid**	17	0.951	0.469	0.065	0.814
***Bison***	**basFro**	23	0.910	0.041*	0.097	0.063
	**ectEct**	23	0.871	0.007*	0.062	0.703
	**proNas**	20	0.954	0.431	0.063	0.782
	**nasNuc**	23	0.897	0.022*	0.071	0.456
	**basTip**	18	0.947	0.376	0.080	0.438
	**ectPro**	20	0.979	0.914	0.066	0.702
	**basWid**	23	0.840	0.002*	0.078	0.296

W statistics (Shapiro-Wilk test for normality), dip statistics (Hartigan’s dip test for unimodality), and associated p-values for the distributions of nine cranial metrics in *Uintatherium anceps* and seven cranial metrics in *Bison bison*. Asterisks indicate significant p-values (⍺ = 0.05). **Abbreviations**: nasOcc, length from nasals to occipital crest; nasCon, length from nasals to occipital condyles; preCon, length from premaxillaries to occipital condyles; occCon, height from occipital condyle to occipital crest; parPro, height of parietal protuberance; maxAlv, height of maxillary protuberance; alvCan, length of canine from alveolus to tip; diaLen, diastemal length; canWid, width of canine at alveolus; basFro, width between bases of cornual processes; ectEct, width between left and right ectorbitale; proNas, length from prosthion to nasion; nasNuc, length from nasion to nuchal line; basTip, length of cornual process; ectPro, length from prosthion to ectorbitale; basWid, width of cornual process at base.

### Mixture modeling

Among six of the nine cranial metrics analyzed in uintatheres, the preferred model only had one component, and no metric was best fit by a model with two components (Table 2). Among bison, two components were preferred for three of the selected cranial metrics (Table 2).

**Table 2:** Number of components preferred by mixture modeling for each cranial metric in *Uintatherium anceps* and *Bison bison.*

Genus	Measurement	Components	BIC
***Uintatherium***	**nasOcc**	1	−e155.804
	**nasCon**	1	−125.078
	**preCon**	1	−89.284
	**occCon**	1	−95.801
	**parPro**	1	−122.402
	**maxAlv**	1	−125.158
	**alvCan**	9	−32.024
	**diaLen**	3	−59.010
	**canWid**	6	−45.871
***Bison***	**basFro**	2	−121.150
	**ectEct**	2	−116.173
	**proNas**	1	−86.665
	**nasNuc**	3	−110.169
	**basTip**	1	−102.001
	**ectPro**	1	−81.890
	**basWid**	2	−76.239

**Abbreviations**: BIC, Bayesian Information Criterion; nasOcc, length from nasals to occipital crest; nasCon, length from nasals to occipital condyles; preCon, length from premaxillaries to occipital condyles; occCon, height from occipital condyle to occipital crest; parPro, height of parietal protuberance; maxAlv, height of maxillary protuberance; alvCan, length of canine from alveolus to tip; diaLen, diastemal length; canWid, width of canine at alveolus; basFro, width between bases of cornual processes; ectEct, width between left and right ectorbitale; proNas, length from prosthion to nasion; nasNuc, length from nasion to nuchal line; basTip, length of cornual process; ectPro, length from prosthion to ectorbitale; basWid, width of cornual process at base.

### Principal component analysis and k-means clustering

In multivariate analyses, 18 uintathere skulls remained after imputation, including three that have been considered females by previous authors and three which have been considered males [73,75,78].

Together, PC1 and PC2 account for over 80% of observed variance in *U. anceps*, about 60% and 20%, respectively (S4 Table). PC1 is positively correlated with all variables, while PC2 is positively correlated with the length and width of the canine and the height of the maxillary and parietal horns, but negatively correlated with variables capturing the total length of the skull (S5 Table). Thus, PC1 seems to reflect the overall size of the skull while PC2 mostly captures the relative prominence of the horns and canine. All purported female specimens show low to moderate PC1 and PC2 values; purported males vary widely in their PC1 and PC2 values (Fig 5A). Results of PCA and PERMANOVA do not indicate significant differences between purported male and female specimens (p > 0.05) (S4 File).

**Fig 5:**
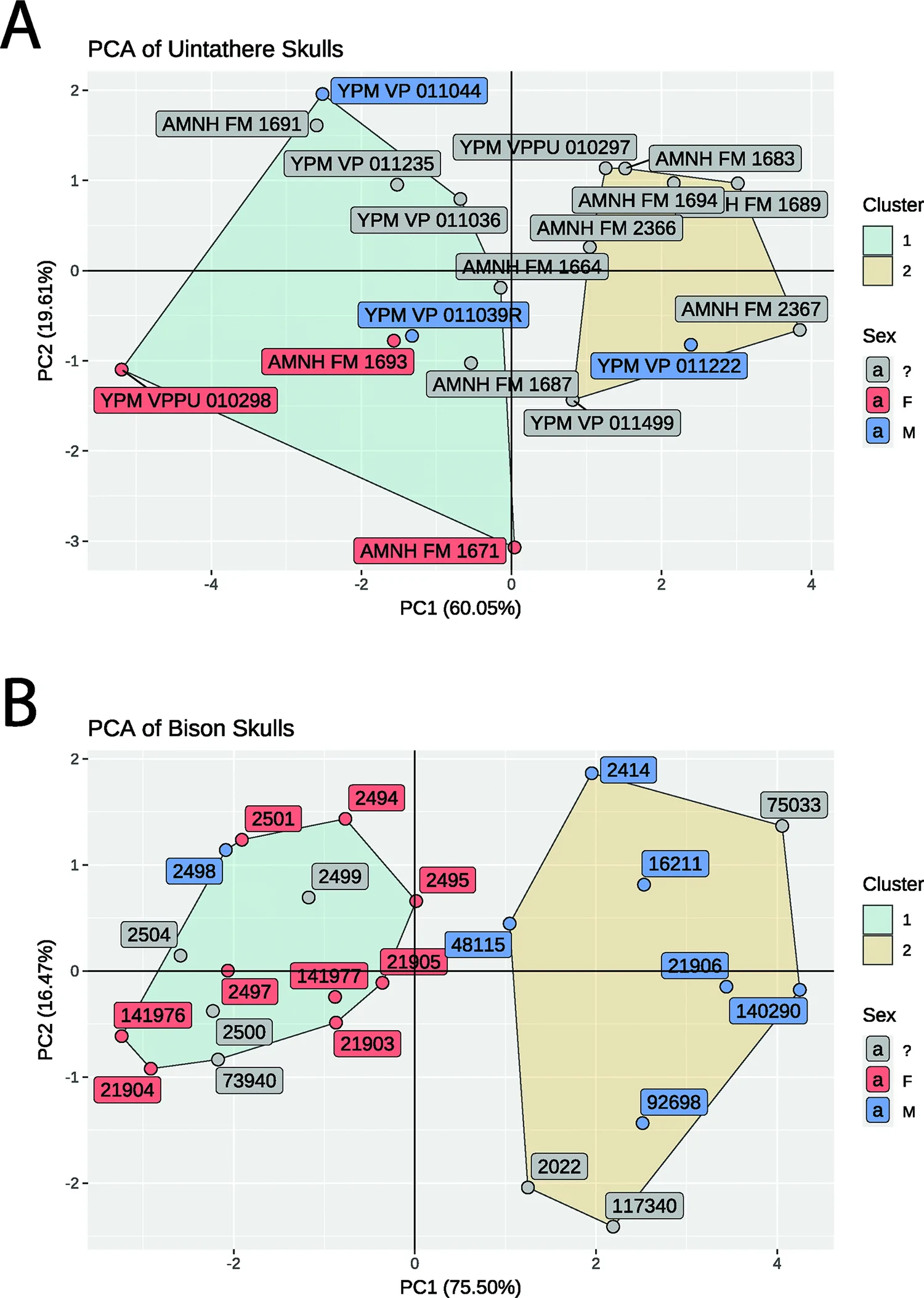
Results of PCA in *Uintatherium anceps* and *Bison bison.* (**A**) Bivariate plot displaying values of PC1 and PC2 for each specimen of *Uintatherium anceps*. Specimens labeled in red represent purported females, blue specimens are purported males, and gray specimens have not been attributed to either sex by previous authors. Teal and gold hulls represent clusters as designated by k-means clustering. (**B**) Bivariate plot displaying values of PC1 and PC2 for each specimen of *Bison bison*. Specimens labeled in red represent females, blue specimens are males, and gray specimens lacked sex metadata. As in *Uintatherium*, teal and gold hulls represent clusters as designated by k-means clustering.

The two most favorable clusters obtained through k-means cluster analysis (n = 8 and n = 10) are separated primarily along the PC1 axis (Fig 5A). Cluster 1 includes all purported females and two purported males, while cluster 2 contains the third proposed male. Of the three methods employed to determine the optimal number of clusters, only the silhouette method clearly favors two (S10 Fig).

In *Bison*, all variables contribute positively to PC1, while only the variables capturing the length of the anterior portion of the skull contribute positively to PC2 (S7 Table). Females have generally negative PC1 scores, while all but one male have positive PC1 scores (Fig 5B). Both sexes vary in their PC2 scores. When the two most favorable clusters are formed through k-means clustering, all females are assigned to the same cluster, while all males except for one fall into the other (Fig 6B). All three methods employed favor two clusters in *Bison* (S11 Fig). Results of PCA and PERMANOVA indicate significant differences between male and female specimens of *Bison* (p < 0.05) (S5 File).

**Fig 6:**
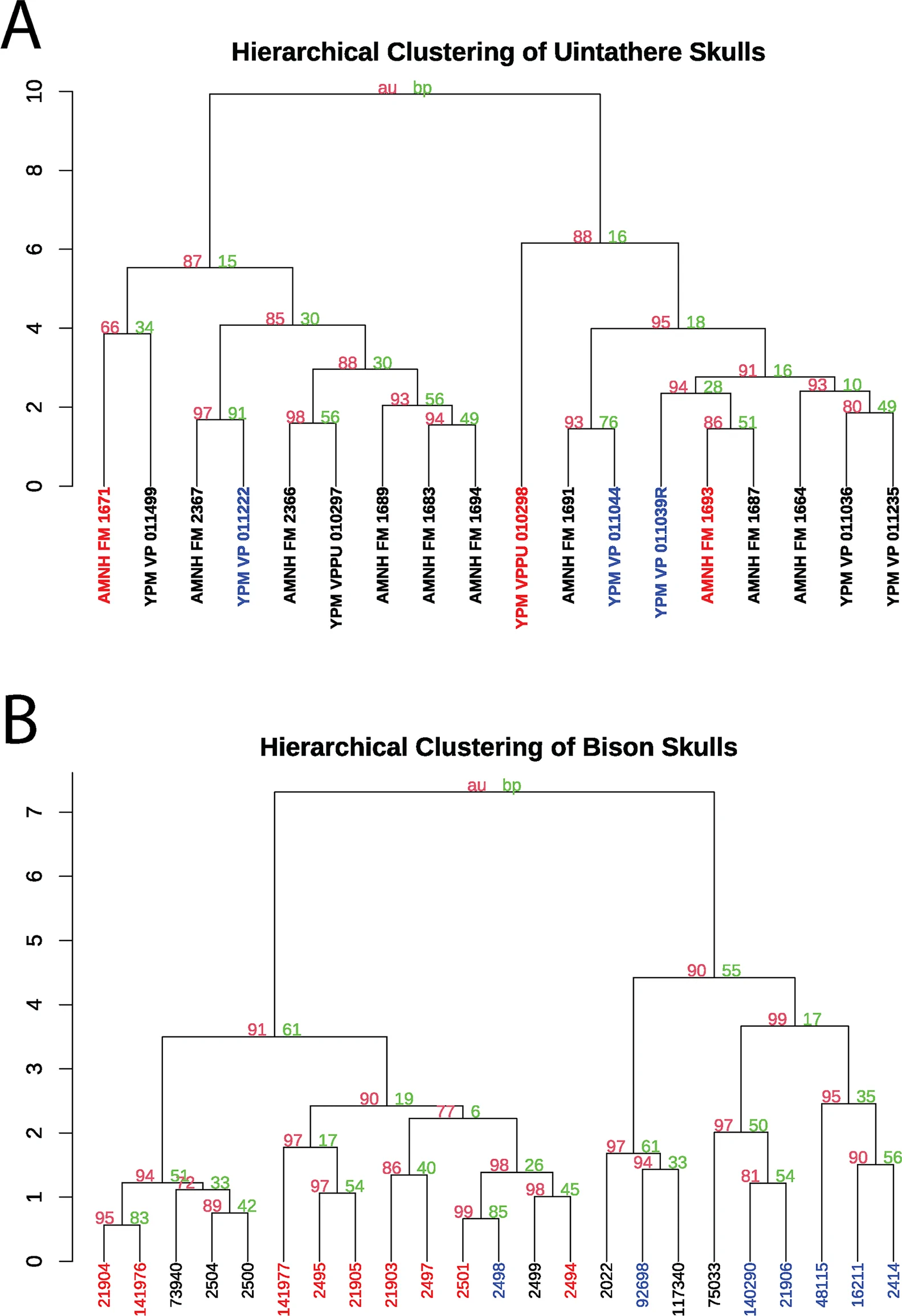
Hierarchical clustering of *Uintatherium anceps* and *Bison bison* crania. **(A)** Dendrogram displaying hierarchical clustering of *Uintatherium* skulls. Numbers above nodes represent support for a given cluster (higher values = more support). Specimens labeled in red and blue represent purported females and males according to previous authors, respectively. **(B)** Dendrogram of *Bison* skulls. Specimens labeled in red and blue represent females and males, respectively. All *Bison* specimens curated at the University of Kansas Natural History Museum (KU:Mamm). **Abbreviations**: au, approximately unbiased p-value; bp, bootstrap probability.

### Hierarchical clustering

In hierarchical clustering of *U. anceps*, purported females and males occur apparently at random across the dendrogram and do not cluster at all according to their hypothesized sex (Fig 6A and S12 Fig). Likewise, putative females and males do not appear to differ consistently in morphology, as they vary widely in most metrics (S12 Fig).

Bison skulls form two primary clusters, and with only one exception, all females fall in one cluster while all males fall in the other (Fig 6B and S12 Fig). Males are consistently larger than females, especially in metrics related to the size of the cornual processes and the width of the skull (S12 Fig).

### Variance-based estimates of sexual dimorphism in *Uintatherium*

Implementation of the means method (MM) and finite mixture analysis (FMA) indicate varying degrees of sexual dimorphism among the traits measured in *U. anceps*. Metrics related to horn and canine size exhibit higher estimates of sexual dimorphism than those capturing the overall size of the skull (Table 3). A similar pattern is observed in *Bison* (Table 3). Estimates of sexual dimorphism in skull length for *Uintatherium* obtained by MM and FMA approach but fall below the highest values seen in extant mammalian herbivores (Table 4).

**Table 3:** Estimated sexual dimorphism indices for cranial metrics in *Uintatherium anceps* and *Bison bison.*

Genus	Measurement	MM	FMA
***Uintatherium***	**nasOcc**	1.123	1.142
	**nasCon**	1.152	1.136
	**preCon**	1.145	1.112
	**occCon**	1.192	1.158
	**parPro**	1.244	1.239
	**maxAlv**	1.349	1.275
	**alvCan**	1.450	1.438
	**diaLen**	1.243	1.199
	**canWid**	1.266	1.233
***Bison***	**basFro**	1.292	1.173
	**ectEct**	1.242	1.136
	**proNas**	1.121	1.106
	**nasNuc**	1.257	1.162
	**basTip**	1.438	1.325
	**ectPro**	1.084	1.074
	**basWid**	1.658	1.340

Sexual dimorphism indices (SDI; ratio of male to female mean) as estimated by means method (MM) and finite mixture analysis (FMA) for cranial metrics in *Uintatherium* and *Bison*. **Abbreviations**: MM, means method; FMA, finite mixture analysis; nasOcc, length from nasals to occipital crest; nasCon, length from nasals to occipital condyles; preCon, length from premaxillaries to occipital condyles; occCon, height from occipital condyle to occipital crest; parPro, height of parietal protuberance; maxAlv, height of maxillary protuberance; alvCan, length of canine from alveolus to tip; diaLen, diastemal length; canWid, width of canine at base; basFro, width between bases of cornual processes; ectEct, width between left and right ectorbitale; proNas, length from prosthion to nasion; nasNuc, length from nasion to nuchal line; basTip, length of cornual process; ectPro, length from prosthion to ectorbitale; basWid, width of cornual process at base.

**Table 4:** Estimates of sexual dimorphism in the skull lengths of *Uintatherium* and various extant mammalian herbivores.

Taxon	Order	SDI	REF
*Alces alces*	Artiodactyla	1.06	[110]
*Bison bonasus*	Artiodactyla	1.063	[89]
*Bos gaurus*	Artiodactyla	1.194	[114]
*Camelus dromedarius*	Artiodactyla	1.067	[118]
*Capra pyrenaica*	Artiodactyla	1.098	[116]
*Cervus canadensis roosevelti*	Artiodactyla	1.072	[123]
*Cervus elaphus*	Artiodactyla	1.18	[112]
*Dama dama*	Artiodactyla	1.086	[113]
*Gazella bennettii*	Artiodactyla	1.126	[117]
*Hydropotes inermis argyropus*	Artiodactyla	0.968	[111]
*Megaloceros giganteus*	Artiodactyla	1.052	[121]
*Oryx gazella*	Artiodactyla	0.99	[124]
*Ovis aries*	Artiodactyla	1.005	[126]
*Pecari tajacu*	Artiodactyla	1	[115]
*Taurotragus derbianus*	Artiodactyla	1.05	[124]
*Tayassu pecari*	Artiodactyla	0.991	[115]
*Tragelaphus angasii*	Artiodactyla	1.15	[124]
*Dicerorhinus sumatrensis*	Perissodactyla	1.072	[125]
*Equus ferus caballus*	Perissodactyla	1.067	[29]
*Rhinoceros unicornis*	Perissodactyla	0.991	[7]
*Tapirus terrestris*	Perissodactyla	0.992	[31]
*Bradypus variegatus*	Pilosa	0.995	[119]
*Choloepus didactylus*	Pilosa	1.03	[119]
*Choloepus hoffmani*	Pilosa	0.978	[119]
*Elephas maximus*	Proboscidea	1.094	[120]
*Mammuthus meridionalis*	Proboscidea	1.146	[122]
*Bison bison*	Artiodactyla	1.097-1.116	
*Uintatherium anceps*	Dinocerata	1.123-1.142	

SDI in extant herbivores calculated as the ratio of male mean to female mean. Estimates of SDI in *Uintatherium anceps* and *Bison bison* calculated using MM and FMA. **Abbreviations:** SDI, sexual dimorphism index (male mean/female mean); REF, reference; MM, means method; FMA, finite mixture analysis.

## Discussion

Of all approaches employed, none provide evidence for an extreme degree of sexual dimorphism in *Uintatherium*. Results are generally indistinguishable from monomorphy or suggest only moderate sexual dimorphism. Comparative application of the same approaches in *Bison* reveal stronger signals of consistent sexual dimorphism. These findings indicate that *U. anceps* may have been less sexually dimorphic than previous authors have postulated, or at least that evidence of strong sexual dimorphism is currently lacking in this taxon.

Statistical tests fail to reject normality and unimodality in all uintathere metrics except canine length (Table 1). While there were four significant results in *Bison* (Table 1), failure to reject unimodality and normality in several known dimorphic traits reaffirms previous authors’ findings that classical univariate statistical tests lack sufficient power to detect sexual dimorphism in most cases. Univariate mixture modeling appeared to perform marginally better at detecting dimorphism than tests of normality and unimodality, but these results were also ambiguous (Table 2). Due to the small sample size, results of Mann-Whitney U tests cannot suggest an absence of sexual dimorphism in *Uintatherium*, but they do indicate that purported female and male uintathere specimens cannot be statistically distinguished for most chosen metrics. As such, previous authors’ conclusions regarding which individuals represent females and males may be unfounded.

Potentially more informative were the results of multivariate analyses. In hierarchical clustering of *Bison*, skulls were split into two primary clusters, which correspond well with sex (Fig 5B). However, in *Uintatherium*, purported females and males were distributed widely and unpredictably across the dendrogram (Fig 5A). This suggests either that hierarchical clustering in the current sample of *Uintatherium* crania does not correlate with sex or that previous authors have been incorrect in their interpretation of which specimens belong to which sex. This finding is corroborated by results of PCA and k-means cluster analysis, which suggest that purported male and female uintatheres cannot be distinguished by traditional morphometric approaches (Fig 6A). Likewise, use of PCA and PERMANOVA also suggests that purported female and male uintatheres differ less consistently than do female and male bison. The favoring of two clusters in *Bison* (S11 Fig), and the fact that these two clusters separate largely by sex (Fig 6B), further suggest that the expected signals of dimorphism in *Uintatherium* as postulated by previous authors are lacking in this sample of characters.

Implementation of the means method (MM) and finite mixture analysis (FMA) may provide some evidence for sexual dimorphism in *U. anceps*. As would be expected if *Uintatherium* exhibited sexual dimorphism in the prominence of their horns and canines, these traits are predicted to exhibit measurably higher degrees of dimorphism than other cranial metrics (Table 3). Additionally, in skull length, the only metric directly comparable between the two taxa, *Uintatherium* is predicted to have been slightly more dimorphic than *Bison* (Tables 3 and 4). Estimates of dimorphism in skull length for *Uintatherium* obtained through MM and FMA are higher than most extant mammalian herbivores, but these are likely to be overestimates (Table 4, Fig 7). Taken together, these results suggest that *Uintatherium* exhibited some sexual dimorphism in its cranial features, but that it was by no means extreme in comparison to modern herbivorous mammals.

**Fig 7:**
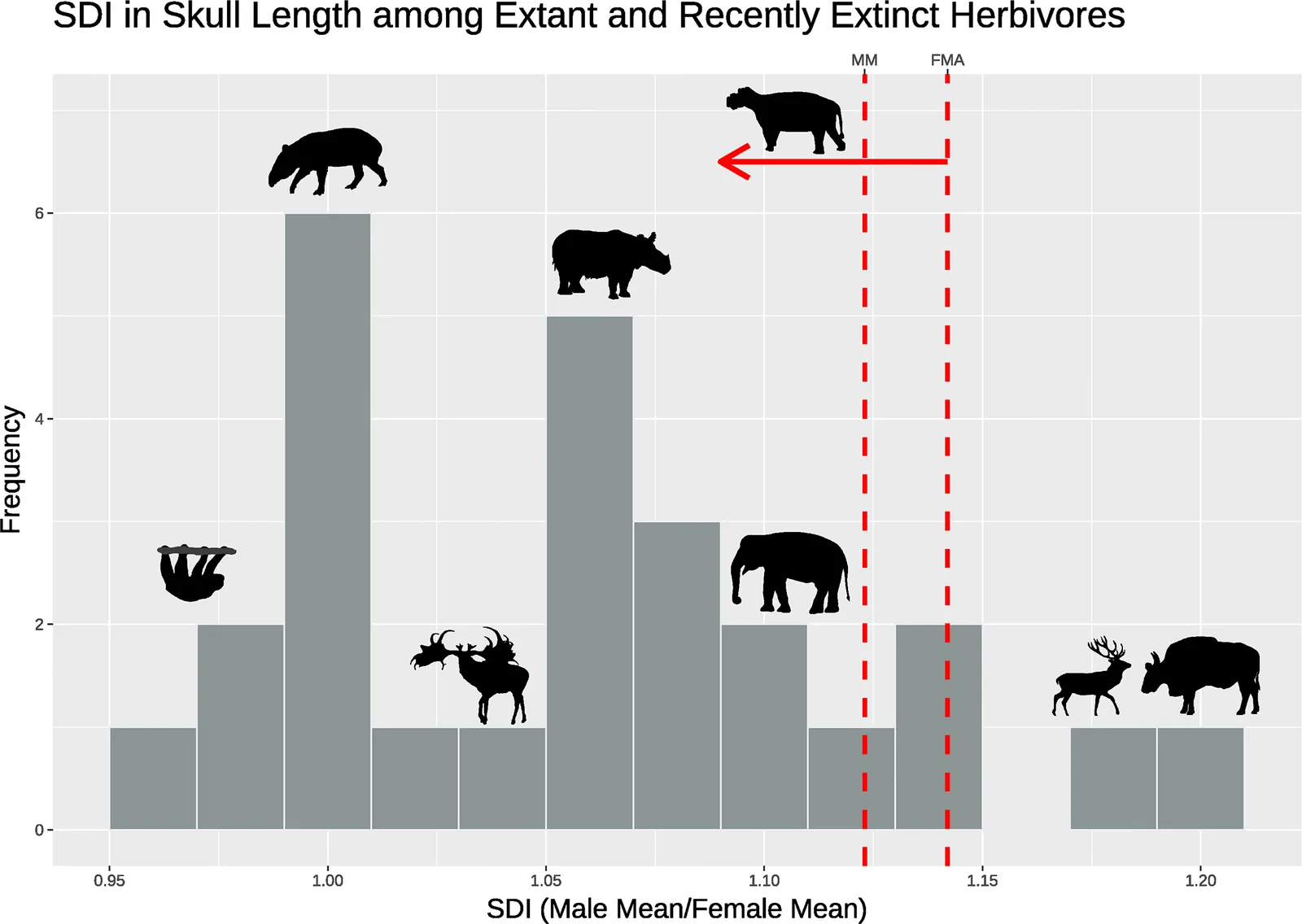
Variation in sexual dimorphism in skull length among extant and recently extinct mammalian herbivores. Histogram displays frequency of skull length SDI values among extant and recently extinct mammalian herbivores in Table 4. Red lines indicate estimates of sexual dimorphism for skull length in *Uintatherium anceps* obtained using MM and FMA. Red arrow is included as these should be treated as maximum estimates. Reconstructions of selected herbivores (*Choloepus*, *Tapirus*, *Megaloceros*, *Dicerorhinus*, *Elephas*, *Cervus*, and *Bos*) reprinted from Phylopic under CC0 1.0 Universal Public Domain Licenses. Reconstruction of *Uintatherium* reprinted from Phylopic user ‘*Zimices*’ under a CC BY 3.0 License (https://creativecommons.org/licenses/by/3.0/). No changes were made to any of the reconstructions. **Abbreviations**: SDI, sexual dimorphism index; MM, means method, FMA; finite mixture analysis.

Heavily biased sex ratios have been documented elsewhere in the fossil record, and it is possible that a bias towards male specimens, as has been posited by previous authors, may obscure statistical signals of sexual dimorphism in *Uintatherium* [56,75,78,127,128]. However, research has shown that male and female mammals are subject to approximately equivalent postmortem preservation, making a taphonomic bias towards male uintatheres unlikely [128]. Collection bias towards larger, more robust specimens with prominent horns has been proposed as another potential explanation for disproportionate representation of males among the current sample of uintatheres, but, given both sexes’ immense size and bizarre appearance in comparison to coeval mammals, this argument may be uncompelling [75,76,127]. Furthermore, while male-biased sex ratios are well-documented in the mammalian fossil record, confirmed instances have been largely confined to polygynous species of herding mammals [127–129]. Such taxa are often characterized by wide-ranging males that are more broadly distributed across a landscape, and therefore more likely to fossilize [127–129]. Unlike other similarly sized and contemporaneous mammals, however, no uintathere death assemblage has ever been recovered, and thus there is no compelling evidence that they lived in polygynous herds [130,131]. Given the warm, wet, rugged, and heavily forested landscape in which uintatheres appear to have lived, it is perhaps more reasonable to infer that uintatheres were largely solitary, and that females and males were subject to similar spatial distributions [80,132–135].

It is also possible that the reconstruction techniques employed by previous researchers obscure signal of sexual dimorphism among these skulls of *U. anceps*. As these reconstructed portions were designed to be generally indistinguishable from the original fossil material, it was largely impossible to exclude them from the analyses. As such, the range of morphological variation observed in the crania of *U. anceps* is likely influenced to some degree by decisions made by previous researchers as to how these skulls should be reconstructed. Nonetheless, the error introduced by these reconstruction techniques should not be expected to wholly obfuscate signals of sexual dimorphism. These skulls were reconstructed to reflect the perspectives of previous curators and researchers, most of whom believed that uintatheres were strongly dimorphic. As such, if anything, it might be expected that the horns and canines on the largest skulls should be artificially exaggerated. In this light, it is perhaps even more noteworthy that evidence for strong sexual dimorphism in *U. anceps* is generally lacking.

These results should not lead one to conclusively reject the hypothesis that *U. anceps* was sexually dimorphic, nor do they show that the taxon was totally or nearly monomorphic. Instead, they refute the classical interpretation that the species exhibits evidence of extreme sexual dimorphism [73,75,78]. These results show that previous conceptualizations of sexual dimorphism in *Uintatherium* lack statistical support. Given their unresolved phylogenetic affinities and the clade-specific nature of sexual dimorphism among extant mammals, there is little justification for assuming that uintatheres were strongly sexually dimorphic a priori. Large herbivorous extant mammals with apparently similar ecological preferences have been shown to exhibit limited degrees of dimorphism, so it is entirely plausible that these taxa could have been only moderately dimorphic [7,136].

It is interesting to consider what purpose the elaborate cranial features of uintatheres may have served if they were not highly sexually dimorphic. Purely functional explanations (e.g., roles in feeding for the canines or muscle attachment for the protuberances) seem unreasonable given the extreme and unusual morphologies of these structures. Extant species of large mammalian herbivores with cranial armaments vary in whether these features are expressed in only one or both sexes [137,138]. Even for neontologists, the presence of horns and similar structures in the females of taxa which exhibit polygynous mating systems, male-male competition, and/or female choice is somewhat perplexing. Explanations ranging from intrasexual competition between females, to mutual mate choice, to generalized defense against predators have been proposed, but no consensus has been reached regarding the emergence of apparently costly structures in both sexes [139–145]. The potential presence of large horns and canines in both male and female uintatheres adds to this apparent gap in our current theory of sexual selection. The repeated evolution of cranial armaments among both sexes of mammalian herbivores may reflect general constraints in the development of such structures across all individuals of a single species [146,147]. Enlarged horns and canines in uintatheres may very well have evolved as male sexual display structures; nonetheless, shared developmental architecture may have ensured their development, at least to some degree, in both sexes.

Taken together, the results of this study do not preclude the presence of sexual dimorphism in Dinocerata altogether; to the contrary, they provide the first quantitative evidence of at least modest dimorphism in uintathere canine length and horn size (Tables 1 and 3, S8 Fig). Traits not examined in this study, such as the inframandibular flange, may have been subject to different patterns of sexual variation. Patterns of sexual dimorphism may have also varied between uintathere taxa, as in extant mammalian orders [10,119,148]. Nonetheless, these results suggest that reinterpretation of uintatheres, particularly *U. anceps*, as only slightly or moderately dimorphic represents a viable hypothesis in light of currently available data. Only by analyzing additional taxa, applying more robust morphometric techniques, and uncovering additional fossil specimens can we make further progress in assessing sexual dimorphism among uintatheres.

Given the potentially basal position of uintatheres on the placental phylogeny, these results might also indicate that strong sexual dimorphism was not ancestral among the infraclass. Similar analyses in other groups of early-diverging clades are needed to further assess the evolution of sexual dimorphism among placental mammals. The linear morphometric approach employed here is easily reproducible and broadly applicable in the study of sexual dimorphism among other extinct animals. Through a quantitative, comparative framework, we may begin to understand when and how many times sexual dimorphism has evolved across the tree of life.

## Supporting information

S1 Table Uintathere cranial measurements, abbreviations, and definitions as used in this study. All measurements obtained in ImageJ.
(DOCX)

S2 Table Bison cranial measurements, abbreviations, and definitions as used in this study. All measurements obtained in ImageJ.
(DOCX)

S3 Table Tests for measurement error in *Uintatherium anceps* and *Bison bison* cranial metrics. Results of linear models evaluating the relationship between the mean of hand-measured and ImageJ values and the absolute and proportional difference between these two values for each cranial metric in *Uintatherium anceps* and *Bison bison*. **Abbreviations**: n, number of specimens for which values were obtained both by hand and by ImageJ for each metric.
(DOCX)

S4 Table Eigenvalues of and variance explained by each component in principal component analysis of *Uintatherium anceps* cranial metrics. **Abbreviations**: PC, principal component.
(DOCX)

S5 Table Principal component loadings of cranial metrics in *Uintatherium anceps.* **Abbreviations**: PC, principal component.
(DOCX)

S6 Table Eigenvalues of and variance explained by each component in principal component analysis of *Bison bison* cranial metrics. **Abbreviations**: PC, principal component.
(DOCX)

S7 Table Principal component loadings of cranial metrics in *Bison bison.* **Abbreviations**: PC, principal component.
(DOCX)

S1 Figure Proportional difference between manually measured and ImageJ values for cranial metric in *Uintatherium anceps.* Plots display the percentage difference in the hand-measured and ImageJ values as a function of the mean of the two values for each cranial metric in *Uintatherium anceps*. (A), length of skull from nasals to occipital crest; (B), length of skull from nasals to occipital condyles; (C), length of skull from premaxillaries to occipital condyles; (D), height of skull from top of occipital crest to bottom of occipital condyle; (E), height of skull from top parietal protuberance to bottom of postglenoid process; (F), height of maxillary protuberance; (G), canine length; (H), diastemal length.
(TIF)

S2 Figure Proportional difference between manually measured and ImageJ values for cranial metric in *Bison bison.* Plots display the percentage difference in the hand-measured and ImageJ values as a function of the mean of the two values for each cranial metric in *Bison bison*. (A), width between bases of cornual processes; (B), width between left and right ectorbitale; (C), length from prosthion to nasion; (D), length from nasion to nuchal line; (E), length of cornual process; (F), length from prosthion to ectorbitale.
(TIF)

S3 Figure Bland-Altman plots for each cranial metric in *Uintatherium anceps.* Plots display the relationship between the mean of hand-measured and ImageJ values and the difference between hand-measured and ImageJ values for each cranial metric in *Uintatherium anceps*. (A), length of skull from nasals to occipital crest; (B), length of skull from nasals to occipital condyles; (C), length of skull from premaxillaries to occipital condyles; (D), height of skull from top of occipital crest to bottom of occipital condyle; (E), height of skull from top parietal protuberance to bottom of postglenoid process; (F), height of maxillary protuberance; (G), canine length; (H), diastemal length.
(TIF)

S4 Figure Bland-Altman plots for each cranial metric in *Bison bison.* Plots display the relationship between the mean of hand-measured and ImageJ values and the difference between hand-measured and ImageJ values for each cranial metric in *Bison bison*. (A), width between bases of cornual processes; (B), width between left and right ectorbitale; (C), length from prosthion to nasion; (D), length from nasion to nuchal line; (E), length of cornual process; (F), length from prosthion to ectorbitale.
(TIF)

S5 Figure Effects of scalebar placement and camera angle in measurements of *Bison* cranial metrics. Plots display the effects of differing camera angle and scale bar placement on repeated measurements of the same metrics in *Bison*. **Abbreviations**: basFro, width between bases of cornual processes; ectEct, width between left and right ectorbitale; proNas, length from prosthion to nasion; nasNuc, length from nasion to nuchal line; basTip, length of cornual process; ectPro, length from prosthion to ectorbitale; basWid, width of cornual process at base.
(TIF)

S6 Figure Effects of camera angle and zoom in measurements of *Bison* cranial metrics. Plots display the effects of differing camera angle and zoom on repeated measurements of the same metrics in *Bison*. Abbreviations: basFro, width between bases of cornual processes; ectEct, width between left and right ectorbitale; proNas, length from prosthion to nasion; nasNuc, length from nasion to nuchal line; basTip, length of cornual process; ectPro, length from prosthion to ectorbitale; basWid, width of cornual process at base.
(TIF)

S7 Figure Linear regression of *Bison* skull length in the frontal and sagittal planes. Plot generated using available skulls of *Bison* from Morphosource. **Abbreviations:** m, meters.
(TIF)

S8 Figure Distributions of cranial metrics in *Uintatherium anceps.* Random normal data generated with n, mean, and standard deviation equal to that of parPro. **Abbreviations:** nasOcc, length from nasals to occipital crest; nasCon, length from nasals to occipital condyles; preCon, length from premaxillaries to occipital condyles; occCon, height from occipital condyle to occipital crest; parPro, height of parietal protuberance; maxAlv, height of maxillary protuberance; alvCan, length of canine from alveolus to tip; diaLen, diastemal length; canWid, canine width.
(TIF)

S9 Figure Distributions of cranial metrics in *Bison bison.* **Abbreviations**: basFro, width between bases of cornual processes (frontal horns); ectEct, width between left and right ectorbitale; proNas, length from prosthion to nasion; nasNuc, length from nasion to nuchal line; basTip, length of cornual process; ectPro, length from prosthion to ectorbitale; basWid, width of cornual process at base.
(TIF)

S10 Figure Number of clusters present in *Uintatherium anceps* crania. Optimal number of clusters in *Uintatherium* data as indicated by total within sum of squares, average silhouette width, and gap statistic.
(TIF)

S11 Figure Number of clusters present in *Bison bison* crania. Optimal number of clusters in *Bison* data as indicated by total within sum of squares, average silhouette width, and gap statistic.
(TIF)

S12 Figure Hierarchical clustering of *Uintatherium anceps* and *Bison bison* crania. **(A)** Heat map displaying hierarchical clustering of *Uintatherium* skulls. Skulls labeled in red and blue represent purported females and males according to previous authors, respectively. **(B)** Heat map of *Bison* skulls. All specimens curated at the University of Kansas Natural History Museum (KU:Mamm). Specimens labeled in red and blue represent females and males, respectively. **Abbreviations**: nasOcc, length from nasals to occipital crest; nasCon, length from nasals to occipital condyles; preCon, length from premaxillaries to occipital condyles; occCon, height from occipital condyle to occipital crest; parPro, height of parietal protuberance; maxAlv, height of maxillary protuberance; alvCan, length of canine from alveolus to tip; diaLen, diastemal length; canWid, canine width; basFro, width between bases of cornual processes (frontal horns); ectEct, width between left and right ectorbitale; proNas, length from prosthion to nasion; nasNuc, length from nasion to nuchal line; basTip, length of cornual process; ectPro, length from prosthion to ectorbitale; basWid, width of cornual process at base.
(TIF)

S1 File Data used in analyses. Sheet 1, uintathere data; sheet 2, bison data.
(XLSX)

S2 File Supplemental information and data. Sheet 1, locality information for uintathere specimens; sheet 2, measurements of uintathere skulls gathered by hand and used in tests for correlation between skull size and measurement error; sheet 3, measurements of uintathere skulls obtained through ImageJ and used in tests for correlation between skull size and measurement error; sheet 4, measurements of bison skulls gathered by hand and used in tests for correlation between skull size and measurement error; sheet 5, mean number of standard deviations in the expected observed range for samples of different size. Used in finite mixture analysis. Adapted from Pearson (1932) and Godfrey (1993) [66,67]; sheet 6, measurements of bison skull with differing camera angle, zoom, and scale bar placement. Used in simulation tests to quantify error in measuring skulls using ImageJ.
(XLSX)

S3 File Supplemental methods and results. Methods used in sensitivity analyses and simulations with summary of results.
(DOCX)

S4 File Code used in morphometric analyses of uintathere crania. (HTML)

S5 File Code used in morphometric analyses of bison crania. (HTML)

S6 File Code used in sensitivity analyses. (HTML)

S7 File Code used in simulations. (HTML)
