# Supplementary material for: Linear morphometrics fail to support strong sexual dimorphism in Uintatherium anceps
Source: PLoS One. 2026 Oct 7;21(10):e0345236. doi: 10.1371/journal.pone.0345236 (PMC13645082; doi:10.1371/journal.pone.0345236)
Supplement: Supplementary file 1 [file pone.0345236.s001.docx]

| **Measurement** | **Abbreviation** | **Definition** |
| --- | --- | --- |
| **Length from nasals to occipital crest** | nasOcc | Distance from the anteriormost point of the nasals or prenasals (if present) to the posteriormost point of the lateral crest |
| **Length from nasals to occipital condyles** | nasCon | Distance from the anteriormost point of the nasals or prenasals (if present) to the posteriormost point of the occipital condyle |
| **Length from premaxillaries to occipital condyles** | preCon | Distance from the anteriormost point of the premaxillaries to the posteriormost point of the occipital condyle |
| **Height from occipital crest to occipital condyles** | occCon | Distance from the inferiormost point of the occipital condyle to the superiormost point of the lateral crest |
| **Height from parietal protuberance to tip of postglenoid process** | parPro | Distance from the dorsalmost tip of the parietal protuberance to the inferiormost tip of the postglenoid process |
| **Height of maxillary protuberance** | maxAlv | Distance from the dorsalmost tip of the maxillary protuberance to the posteriormost point along the canine alveolar border |
| **Length of canine from alveolar border to tip** | alvCan | Distance from the posteriormost point along the canine alveolar border to the distalmost tip of the canine |
| **Diastemal length** | diaLen | Distance from the posteriormost point along the canine alveolar border to the anteriormost point of the P2 alveolar border |
| **Width of canine at base** | canWid | Distance from the anteriormost to the posteriormost points of canine at the alveolar border |
