# Supplementary material for: Linear morphometrics fail to support strong sexual dimorphism in Uintatherium anceps
Source: PLoS One. 2026 Oct 7;21(10):e0345236. doi: 10.1371/journal.pone.0345236 (PMC13645082; doi:10.1371/journal.pone.0345236)
Supplement: Supplementary file 2 [file pone.0345236.s002.docx]

| **Measurement** | **Abbreviation** | **Definition** |
| --- | --- | --- |
| Width between bases of cornual processes | basFro | Distance between the superiormost points of the margins where the smooth texture of the frontal bone becomes rugose on the cornual processes |
| Width between left and right ectorbitale | ectEct | Width between the lateralmost points of the bone around the orbits |
| Length from prosthion to nasion | proNas | Distance from the prosthion to the nasion |
| Length from nasion to nuchal line | nasNuc | Distance from the nasion to the medial point of the nuchal line |
| Length of cornual process | basTip | Distance from the inferiormost point of the margin where the texture of the frontal bone becomes rugose on the cornual process to the distalmost tip of the cornual process |
| Length from prosthion to ectorbitale | ectPro | Distance from the prosthion to the lateralmost point of the orbit |
| Width of cornual process at base | basWid | Distance from the superiormost to the inferiormost points of the margin where the texture of the frontal bone becomes rugose on the cornual process |
