# Supplementary material for: Linear morphometrics fail to support strong sexual dimorphism in Uintatherium anceps
Source: PLoS One. 2026 Oct 7;21(10):e0345236. doi: 10.1371/journal.pone.0345236 (PMC13645082; doi:10.1371/journal.pone.0345236)
Supplement: Supplementary file 3 [file pone.0345236.s003.docx]

| **Taxon** | **Measurement** | **n** | **p-value (absolute)** | **p-value (proportional)** |
| --- | --- | --- | --- | --- |
| ***Uintatherium*** | **nasOcc** | 13 | 0.415 | 0.266 |
|  | **nasCon** | 8 | 0.105 | 0.051 |
|  | **preCon** | 6 | 0.564 | 0.384 |
|  | **occCon** | 8 | 0.655 | 0.413 |
|  | **parPro** | 8 | 0.409 | 0.969 |
|  | **maxAlv** | 10 | 0.911 | 0.631 |
|  | **alvCan** | 6 | 0.342 | 0.372 |
|  | **diaLen** | 10 | 0.397 | 0.363 |
| ***Bison*** | **basFro** | 23 | 0.002* | 0.087 |
|  | **ectEct** | 23 | 0.001* | 0.071 |
|  | **proNas** | 20 | 0.772 | 0.789 |
|  | **nasNuc** | 23 | 0.162 | 0.271 |
|  | **basTip** | 18 | 0.577 | 0.131 |
|  | **ectPro** | 20 | 0.433 | 0.846 |
