# Supplementary material for: Linear morphometrics fail to support strong sexual dimorphism in Uintatherium anceps
Source: PLoS One. 2026 Oct 7;21(10):e0345236. doi: 10.1371/journal.pone.0345236 (PMC13645082; doi:10.1371/journal.pone.0345236)
Supplement: Supplementary file 4 [file pone.0345236.s004.docx]

| **Principal Component** | **Eigenvalue** | **Percentage of Variance** | **Cumulative Percentage of Variance** |
| --- | --- | --- | --- |
| PC1 | 4.34751368 | 58.72546054 | 58.72546054 |
| PC2 | 1.8190263 | 24.57109168 | 83.29655222 |
| PC3 | 0.79178443 | 10.69528671 | 93.99183893 |
| PC4 | 0.18516692 | 2.50120261 | 96.49304154 |
| PC5 | 0.12280083 | 1.65877225 | 98.15181379 |
| PC6 | 0.08213521 | 1.109468152 | 99.26128195 |
| PC7 | 0.04268793 | 0.576621098 | 99.83790304 |
| PC8 | 0.01200022 | 0.162096955 | 100 |
