# Supplementary material for: Linear morphometrics fail to support strong sexual dimorphism in Uintatherium anceps
Source: PLoS One. 2026 Oct 7;21(10):e0345236. doi: 10.1371/journal.pone.0345236 (PMC13645082; doi:10.1371/journal.pone.0345236)
Supplement: Supplementary file 5 [file pone.0345236.s005.docx]

|  | **PC1** | **PC2** | **PC3** | **PC4** | **PC5** |
| --- | --- | --- | --- | --- | --- |
| **Length from nasals to occipital crest** | 0.8738583 | -0.11844 | -0.281266 | 0.2526395 | -0.0400051 |
| **Length from nasals to occipital condyles** | 0.8932335 | -0.36742 | -0.0475787 | 0.02369153 | 0.03218606 |
| **Length from premaxillaries to occipital condyles** | 0.92155999 | -0.3381785 | -0.0048906 | 0.00573459 | -0.0202354 |
| **Height from occipital crest to occipital condyle** | 0.89118645 | 0.11721703 | 0.13414668 | -0.3082779 | -0.0234994 |
| **Height from parietal protuberance to tip of postglenoid process** | 0.67420311 | 0.52211418 | -0.3448777 | -0.0459256 | 0.22264895 |
| **Height of maxillary protuberance** | 0.63977655 | 0.68551437 | 0.06721821 | 0.02363559 | -0.2343069 |
| **Length of canine from alveolus to tip** | -0.120526 | 0.88987768 | 0.14588609 | 0.10553705 | 0.06329886 |
| **Diastemal length** | 0.51394822 | -0.086492 | 0.74003328 | 0.10910965 | 0.10355563 |
