# Supplementary material for: Linear morphometrics fail to support strong sexual dimorphism in Uintatherium anceps
Source: PLoS One. 2026 Oct 7;21(10):e0345236. doi: 10.1371/journal.pone.0345236 (PMC13645082; doi:10.1371/journal.pone.0345236)
Supplement: Supplementary file 6 [file pone.0345236.s006.docx]

| **Principal Component** | **Eigenvalue** | **Percentage of Variance** | **Cumulative Percentage of Variance** |
| --- | --- | --- | --- |
| PC1 | 4.47232548 | 74.5387580 | 74.53876 |
| PC2 | 1.10823535 | 18.4705891 | 93.00935 |
| PC3 | 0.15817414 | 2.6362356 | 95.64558 |
| PC4 | 0.12521915 | 2.0869859 | 97.73257 |
| PC5 | 0.10902581 | 1.8170968 | 99.54967 |
| PC6 | 0.02702008 | 0.4503346 | 100.00000 |
