# Supplementary material for: Linear morphometrics fail to support strong sexual dimorphism in Uintatherium anceps
Source: PLoS One. 2026 Oct 7;21(10):e0345236. doi: 10.1371/journal.pone.0345236 (PMC13645082; doi:10.1371/journal.pone.0345236)
Supplement: Supplementary file 7 [file pone.0345236.s007.docx]

|  | **PC1** | **PC2** | **PC3** | **PC4** | **PC5** |
| --- | --- | --- | --- | --- | --- |
| **Width between bases of cornual processes** | 0.9380201 | -0.15508741 | 0.17822758 | 0.098231295 | -0.23359246 |
| **Width between left and right ectorbitale** | 0.9163598 | -0.25815737 | 0.12602306 | -0.265596801 | 0.08012383 |
| **Length from prosthion to nasion** | 0.7209750 | 0.67785960 | 0.08477941 | -0.006020815 | 0.05203577 |
| **Length from nasion to nuchal line** | 0.7615236 | -0.60378065 | -0.01420894 | 0.165956616 | 0.15950489 |
| **Length of cornual process** | 0.9384320 | -0.03440128 | -0.31884618 | -0.072374602 | -0.10369095 |
| **Length from prosthion to ectorbitale** | 0.8788349 | 0.43853148 | -0.03840477 | 0.110508709 | 0.09559913 |
