# Supplementary material for: Linear morphometrics fail to support strong sexual dimorphism in Uintatherium anceps
Source: PLoS One. 2026 Oct 7;21(10):e0345236. doi: 10.1371/journal.pone.0345236 (PMC13645082; doi:10.1371/journal.pone.0345236)
Supplement: Supplementary file 8 [file pone.0345236.s022.docx]

Supplemental Methods and Results for ‘Linear morphometrics fail to support strong sexual dimorphism in *Uintatherium anceps*’

**Materials and Methods**

**Differences between values obtained manually and through ImageJ**

In addition to being calculated in ImageJ, the value for each cranial metric in *Uintatherium*, with the exception of canine width, was measured manually using a tape measure. Canine width was incorporated into this study later into its development, so it was not measured manually and is thus not included in these sensitivity analyses. For each metric, the percentage difference between the values obtained through ImageJ and manually was calculated by subtracting the ImageJ value from the by-hand measurement and then dividing by the mean of the two values. Bland-Altman plots and linear models were generated to determine if the absolute and proportional difference between the values obtained manually and through ImageJ were correlated with skull size.

The same methods were performed for each metric in *Bison*. As the width of the cornual processes, much like width of canine in *Uintatherium*, was incorporated later in the development of this study, it is not included in these analyses.

**Comparability of skull length as measured in the frontal and sagittal planes**

Published scans of bison skulls were used to demonstrate if skull length measured in the frontal plane was directly comparable to skull length when measured in the sagittal plane. Five scans were obtained from Morphosource, and measurement of skull length from prosthion to the nuchal line was measured in the frontal and sagittal planes using the measurement tool in ‘Morphosource’ (Table 1). A linear model was generated to determine if skull length measured in the frontal plane was a significant predictor of skull length measured in the sagittal plane.

| **Specimen** | **Skull length (frontal, meters)** | **Skull length (sagittal, meters)** | **Hyperlink** | **Project ID** | **Media ID** |
| --- | --- | --- | --- | --- | --- |
| AMNH:Mammals:M-22914 | 0.544 | 0.535 | https://www.morphosource.org/concern/media/000580991?locale=en | 000581124 | 000580991 |
| imnh:r:534 | 0.562 | 0.55 | https://www.morphosource.org/concern/media/000106118?locale=en | 00000C960 | 000106118 |
| imnh:r:163 | 0.552 | 0.55 | https://www.morphosource.org/concern/media/000106630?locale=en | 00000C960 | 000106630 |
| NHMUK:PAL:PV M 15712 | 0.575 | 0.586 | https://www.morphosource.org/concern/media/000831617?locale=en |  | 000831617 |
| nhmuk:194.9.6.29.1 | 0.493 | 0.497 | https://www.morphosource.org/concern/media/000160858?locale=en | 00000C428 | 000160858 |

**Table 1:** Specimens used in test to determine if skull length measured in the frontal plane was directly comparable to skull length when measured in the sagittal plane. All data obtained from Morphosource.

**Simulation to quantify effects of lens distortion, camera angle, and scalebar placement**

A simulated analysis was performed to quantify potential sources of error in measuring skulls using photographs and ImageJ. A complete, but uncatalogued skull of *Bison* in the collections of the University of Kansas Natural History Museum and Biodiveristy Institute was repeatedly photographed under different conditions. Three variables were manipulated: camera angle, scalebar placement, and camera zoom. Pictures were first taken at either a direct orthogonal, high (oriented slightly facing the superior portion of the skull), or low (oriented slightly facing the inferior portion of the skull) angle, and the scalebar was placed atop the skull, on the surface on which the skull was sitting, or on a base approximately halfway between the surface and the closest part of the skull. Three images were taken of the skull in each treatment (same angle and same scalebar placement). The effect of camera zoom was also incorporated by taking additional photos of the skull at a higher zoom (21/35 mm vs 14/35 mm for the rest of the images) with the scalebar placed on a base approximately halfway between the surface and the closest part of the skull. Angle was again manipulated in these zoomed pictures, with three photos taken at orthogonal, high, and low angles, respectively. Two ANOVAs were performed for each metric, one quantifying the relative effects of camera angle and scalebar placement, and one quantifying the relative effects of camera angle and lens zoom. Effect size was calculated for each term using the R package, ‘effectSize’. Results were visualized using base R and ‘ggplot2’.

Within each treatment (constant camera angle, zoom, and scalebar placement), the maximum percentage difference was calculated as the ratio of the largest to the smallest value for each metric in that treatment. The mean and maximum percentage difference across all treatments was calculated. To simulate the effects of this within-treatment error on results, all analyses performed in the main paper were again performed in *Bison* and *Uintatherium*, this time with each measurement allowed to vary by up to the maximum percentage difference observed for a metric in any treatment.

**Results**

**Differences between values obtained manually and through ImageJ**

Measurements obtained manually and through ImageJ were found to differ by up to 40%, though most differences were less drastic (S1-S2 Figs and S6 File). Which of the two sets of measurements are more biologically accurate cannot be determined from these results. In most metrics however, differences were systematic (e.g., all ImageJ values were larger than those obtained by-hand, etc.), indicating it is most important to maintain consistent measurement practices for all specimens (i.e., only use values obtained manually or by ImageJ across all specimens). Results of linear models indicated that the absolute difference between the manual and ImageJ values did not differ with size for any uintathere metric, while results were significant for two metrics in bison (S3-S4 Figs, S3 Table, and S6 File). The proportional difference between the two measurements did not appear to vary significantly with size for any uintathere or bison metric (S3-S4 Figs, S3 Table, S6 File).

**Comparability of skull length as measured in the frontal and sagittal planes**

Even with only five scans available, skull length measured in the frontal plane was found to be a significant predictor of skull length measured in the sagittal plane (p = 0.011, R^2^ = 0.9148) (S7 Fig, S6 File). The two values differed by less than 3% for all specimens (S6 File).

**Simulation to quantify effects of lens distortion, camera angle, and scalebar placement**

Results indicate that scalebar placement had the greatest effect on simulated measurements obtained through ImageJ (S5 Fig, S7 File). Camera angle and zoom, as well as interaction terms, also affected obtained values, though these effects were less extreme for most measurements (S5-S6 Figs, S7 File). When all three variables were held constant, which was done as much as possible in the actual study, values differed by no more than 13.5% and a mean of about 5% (S7 File). Results of uintathere and bison analyses when values were allowed to vary by up to 13.5% from the values presented in manuscript are included in S7 File. While individual results differ from those presented in the paper, general patterns remain the same for *Uintatherium* and *Bison*.
