# Supplementary material for: Linear morphometrics fail to support strong sexual dimorphism in Uintatherium anceps
Source: PLoS One. 2026 Oct 7;21(10):e0345236. doi: 10.1371/journal.pone.0345236 (PMC13645082; doi:10.1371/journal.pone.0345236)
Supplement: Supplementary file 9 [file pone.0345236.s023.html]

Uintathere Analyses


# Uintathere Analyses

#### 2026-07-24

Set wd and load packages

```
#setwd("")

library(dplyr)
```

```
## 
## Attaching package: 'dplyr'
```

```
## The following objects are masked from 'package:stats':
## 
##     filter, lag
```

```
## The following objects are masked from 'package:base':
## 
##     intersect, setdiff, setequal, union
```

```
library(diptest)
library(Amelia)
```

```
## Loading required package: Rcpp
```

```
## ## 
## ## Amelia II: Multiple Imputation
## ## (Version 1.8.3, built: 2024-11-07)
## ## Copyright (C) 2005-2026 James Honaker, Gary King and Matthew Blackwell
## ## Refer to http://gking.harvard.edu/amelia/ for more information
## ##
```

```
library(cluster)
library(ggfortify)
```

```
## Loading required package: ggplot2
```

```
library(ggplot2)
library(ggforce)
library(factoextra)
```

```
## Welcome to factoextra!
```

```
## Want to learn more? See two factoextra-related books at https://www.datanovia.com/en/product/practical-guide-to-principal-component-methods-in-r/
```

```
library(FactoMineR)
library(missMDA)
library(VIM)
```

```
## Loading required package: colorspace
```

```
## Loading required package: grid
```

```
## Registered S3 method overwritten by 'car':
##   method           from
##   na.action.merMod lme4
```

```
## VIM is ready to use.
```

```
## Suggestions and bug-reports can be submitted at: https://github.com/statistikat/VIM/issues
```

```
## 
## Attaching package: 'VIM'
```

```
## The following object is masked from 'package:datasets':
## 
##     sleep
```

```
library(cowplot)
library(plyr)
```

```
## ------------------------------------------------------------------------------
```

```
## You have loaded plyr after dplyr - this is likely to cause problems.
## If you need functions from both plyr and dplyr, please load plyr first, then dplyr:
## library(plyr); library(dplyr)
```

```
## ------------------------------------------------------------------------------
```

```
## 
## Attaching package: 'plyr'
```

```
## The following objects are masked from 'package:dplyr':
## 
##     arrange, count, desc, mutate, rename, summarise, summarize
```

```
library(ggrepel)
library(gridExtra)
```

```
## 
## Attaching package: 'gridExtra'
```

```
## The following object is masked from 'package:dplyr':
## 
##     combine
```

```
library(mclust)
```

```
## Package 'mclust' version 6.1.3
## Type 'citation("mclust")' for citing this R package in publications.
```

```
## 
## Attaching package: 'mclust'
```

```
## The following object is masked from 'package:plyr':
## 
##     count
```

```
## The following object is masked from 'package:VIM':
## 
##     diabetes
```

```
## The following object is masked from 'package:dplyr':
## 
##     count
```

```
library(GGally)
library(BayesFactor)
```

```
## Loading required package: coda
```

```
## Loading required package: Matrix
```

```
## ************
## Welcome to BayesFactor 0.9.12-4.8. If you have questions, please contact Richard Morey (richarddmorey@gmail.com).
## 
## Type BFManual() to open the manual.
## ************
```

```
library(pheatmap)
library(ComplexHeatmap)
```

```
## ========================================
## ComplexHeatmap version 2.24.1
## Bioconductor page: http://bioconductor.org/packages/ComplexHeatmap/
## Github page: https://github.com/jokergoo/ComplexHeatmap
## Documentation: http://jokergoo.github.io/ComplexHeatmap-reference
## 
## If you use it in published research, please cite either one:
## - Gu, Z. Complex Heatmap Visualization. iMeta 2022.
## - Gu, Z. Complex heatmaps reveal patterns and correlations in multidimensional 
##     genomic data. Bioinformatics 2016.
## 
## 
## The new InteractiveComplexHeatmap package can directly export static 
## complex heatmaps into an interactive Shiny app with zero effort. Have a try!
## 
## This message can be suppressed by:
##   suppressPackageStartupMessages(library(ComplexHeatmap))
## ========================================
## ! pheatmap() has been masked by ComplexHeatmap::pheatmap(). Most of the arguments
##    in the original pheatmap() are identically supported in the new function. You 
##    can still use the original function by explicitly calling pheatmap::pheatmap().
```

```
## 
## Attaching package: 'ComplexHeatmap'
```

```
## The following object is masked from 'package:pheatmap':
## 
##     pheatmap
```

```
## The following object is masked from 'package:VIM':
## 
##     prepare
```

```
library(circlize)
```

```
## ========================================
## circlize version 0.4.18
## CRAN page: https://cran.r-project.org/package=circlize
## Github page: https://github.com/jokergoo/circlize
## Documentation: https://jokergoo.github.io/circlize_book/book/
## 
## If you use it in published research, please cite:
## Gu, Z. circlize implements and enhances circular visualization
##   in R. Bioinformatics 2014.
## 
## This message can be suppressed by:
##   suppressPackageStartupMessages(library(circlize))
## ========================================
```

```
library(mixtools)
```

```
## mixtools package, version 2.0.0.1, Released 2022-12-04
## This package is based upon work supported by the National Science Foundation under Grant No. SES-0518772 and the Chan Zuckerberg Initiative: Essential Open Source Software for Science (Grant No. 2020-255193).
```

```
## 
## Attaching package: 'mixtools'
```

```
## The following object is masked from 'package:mclust':
## 
##     dmvnorm
```

```
## The following object is masked from 'package:grid':
## 
##     depth
```

```
library(patchwork)
```

```
## 
## Attaching package: 'patchwork'
```

```
## The following object is masked from 'package:cowplot':
## 
##     align_plots
```

```
library(EnvStats)
```

```
## 
## Attaching package: 'EnvStats'
```

```
## The following object is masked from 'package:Matrix':
## 
##     print
```

```
## The following objects are masked from 'package:stats':
## 
##     predict, predict.lm
```

```
## The following object is masked from 'package:base':
## 
##     print.default
```

```
library(dimorph)
library(effectsize)
library(pvclust)
library(ggnewscale)
library(MASS)
```

```
## 
## Attaching package: 'MASS'
```

```
## The following object is masked from 'package:EnvStats':
## 
##     boxcox
```

```
## The following object is masked from 'package:patchwork':
## 
##     area
```

```
## The following object is masked from 'package:dplyr':
## 
##     select
```

```
library(vegan)
```

```
## Loading required package: permute
```

```
## 
## Attaching package: 'vegan'
```

```
## The following object is masked from 'package:EnvStats':
## 
##     calibrate
```

Load and manipulate data

```
Uintatheres <- read.csv("Uintathere_Measurements.csv", check.names = F) #load dataframe
Uintatheres <- Uintatheres[Uintatheres$Specimen != "YPM VP 011039L",] #remove damaged view of YPM 11039
rownames(Uintatheres) <- Uintatheres[,1] #rename rows as specimen numbers
uinFem <- Uintatheres$Specimen[Uintatheres$sex=="F"]
uinMale <- Uintatheres$Specimen[Uintatheres$sex=="M"]
uinNB <- Uintatheres$Specimen[Uintatheres$sex=="?"]
Uintatheres <- Uintatheres[,4:13] #remove helper variables
#Uintatheres[1,]$alvCan <- NA #This canine is potentially pathologic so it's inclusion is questionable

str(Uintatheres)
```

```
## 'data.frame':    26 obs. of  10 variables:
##  $ nasOcc: num  73.4 65.6 71.4 72.7 75.4 ...
##  $ nasCon: num  73.9 60.2 69.1 67.5 71.7 ...
##  $ preCon: num  68.9 56.9 NA 62.3 66.6 ...
##  $ occCon: num  26.7 22.5 24.8 27.6 30.3 ...
##  $ parPro: num  32.7 37.5 43.2 NA 42.6 ...
##  $ maxAlv: num  19.1 24.3 21.4 28.4 30.5 ...
##  $ maxCan: num  26.7 NA 37 49.9 45.6 ...
##  $ alvCan: num  8.49 NA NA 22.07 NA ...
##  $ diaLen: num  7.92 5.58 7.2 9.08 8.81 ...
##  $ canWid: num  4.75 5.36 5.39 7.11 7.09 ...
```

```
colnames(Uintatheres)
```

```
##  [1] "nasOcc" "nasCon" "preCon" "occCon" "parPro" "maxAlv" "maxCan" "alvCan"
##  [9] "diaLen" "canWid"
```

PART 1: Generate histograms for each variable and test for normality
and unimodality

Setting up “Stats” dataframe to store values of various test
statistics of each measurement

```
columns <- colnames(Uintatheres)
columns <- c("Length from Nasals to Occipital Crest", 
             "Length from Nasals to Occipital Condyles", 
             "Length from Premaxillaries to Occipital Condyles", 
             "Height from Occipital Crest to Occipital Condyles",
             "Height from Parietal Protuberance to tip of Postglenoid Process",
             "Height of Maxillary protuberance from Canine Alveolus", 
             #             "Height from Maxillary Protuberance to Tip of Canine", #For now, I have commented out this measurement
             "Length of Canine from Alveolus to Tip",
             "Diastemal Length",
             "Canine Width at Base")
NVariables <- length(columns)

Uintatheres <- Uintatheres[,-7] #Remove old variable

Measurement <- vector()
n <- vector()
wStatistic <- vector()
pValue <- vector()
dipStatistic <- vector() 

Stats <- data.frame(Measurement, n, wStatistic, pValue, dipStatistic, pValue)
```

RUN for-loop that plots histograms for each variable, runs
Shapiro-Wilk and Hartigan’s Dip Test, and stores these variables in
“Stats” dataframe.

```
for (i in 1:NVariables) {
  histi <- hist(Uintatheres[,i][!is.na(Uintatheres[,i])], main = paste(columns[i]),
                col = "skyblue1", xlab = columns[i], cex.main = 2, cex.lab=1.5)
  lengthi <- unique(Uintatheres[,i][!is.na(Uintatheres[,i])])
  xfit <- seq(min(Uintatheres[,i][!is.na(Uintatheres[,i])], na.rm = T), 
              max(Uintatheres[,i][!is.na(Uintatheres[,i])], na.rm = T), 
              length = length(lengthi)) 
  yfit <- dnorm(xfit, mean = mean(Uintatheres[,i][!is.na(Uintatheres[,i])], na.rm = T), 
                sd = sd(Uintatheres[,i][!is.na(Uintatheres[,i])], na.rm = T)) 
  yfit <- yfit * diff(histi$mids[1:2]) * length(Uintatheres[,i][!is.na(Uintatheres[,i])]) 
  lines(xfit, yfit, col = "red", lwd = 3)
  Stats[i,1] <- columns[i]
  Stats[i,2] <- length(Uintatheres[,i][!is.na(Uintatheres[,i])])
  Stats[i,3] <- shapiro.test(Uintatheres[,i])[1]
  Stats[i,4] <- shapiro.test(Uintatheres[,i])[2]
  Stats[i,5] <- dip.test(Uintatheres[,i])[1]
  Stats[i,6] <- dip.test(Uintatheres[,i])[2]
  mtext(paste("w-statistic: ", Stats[i,3], "; p-value: ", Stats[i,4], "\n",
              "dip-statistic: ", Stats[i,5], "; p-value: ", Stats[i,6],  sep = ""), 
        padj = .3, side = 4, cex = 2)
}
```

```
par(mfrow = c(1,1))
```

Generate Violin Plots for Each Measurement

```
par(mfrow = c(3,3))
par(mar=c(2,2,2,2))

dims <- list(length(colnames(Uintatheres)))
dims[[9]] <- ggplot(data = Uintatheres,
                    mapping = aes(x="", y= `nasals to occipital crest` )) +
  geom_violin( fill = "gray80", trim = F) + geom_point() + geom_boxplot(width = 0.1, fill = "gray70") +
  labs(y = "Length (cm)", x="", title = "Nasals to Occipital Crests")


for (i in 1:length(colnames(Uintatheres))) {
  dims[[i]] <-  ggplot(data = Uintatheres,
                       mapping = aes(x="", y=.data[[colnames(Uintatheres)[i]]])) +
    geom_violin( fill = "gray80", trim = F) + geom_point() + geom_boxplot(width = 0.1, fill = "gray70") +
    labs(y = "Length (cm)", x="", title = colnames(Uintatheres)[i])
}


grid.arrange(dims[[1]], dims[[2]], dims[[3]], dims[[4]], dims[[5]], dims[[6]], dims[[7]], dims[[8]], dims[[9]],
             ncol = 3, nrow = 3)
```

```
## Warning: Removed 2 rows containing non-finite outside the scale range
## (`stat_ydensity()`).
```

```
## Warning: Removed 2 rows containing non-finite outside the scale range
## (`stat_boxplot()`).
```

```
## Warning: Removed 2 rows containing missing values or values outside the scale range
## (`geom_point()`).
```

```
## Warning: Removed 7 rows containing non-finite outside the scale range
## (`stat_ydensity()`).
```

```
## Warning: Removed 7 rows containing non-finite outside the scale range
## (`stat_boxplot()`).
```

```
## Warning: Removed 7 rows containing missing values or values outside the scale range
## (`geom_point()`).
```

```
## Warning: Removed 12 rows containing non-finite outside the scale range
## (`stat_ydensity()`).
```

```
## Warning: Removed 12 rows containing non-finite outside the scale range
## (`stat_boxplot()`).
```

```
## Warning: Removed 12 rows containing missing values or values outside the scale range
## (`geom_point()`).
```

```
## Warning: Removed 7 rows containing non-finite outside the scale range
## (`stat_ydensity()`).
```

```
## Warning: Removed 7 rows containing non-finite outside the scale range
## (`stat_boxplot()`).
```

```
## Warning: Removed 7 rows containing missing values or values outside the scale range
## (`geom_point()`).
```

```
## Warning: Removed 7 rows containing non-finite outside the scale range
## (`stat_ydensity()`).
```

```
## Warning: Removed 7 rows containing non-finite outside the scale range
## (`stat_boxplot()`).
```

```
## Warning: Removed 7 rows containing missing values or values outside the scale range
## (`geom_point()`).
```

```
## Warning: Removed 5 rows containing non-finite outside the scale range
## (`stat_ydensity()`).
```

```
## Warning: Removed 5 rows containing non-finite outside the scale range
## (`stat_boxplot()`).
```

```
## Warning: Removed 5 rows containing missing values or values outside the scale range
## (`geom_point()`).
```

```
## Warning: Removed 16 rows containing non-finite outside the scale range
## (`stat_ydensity()`).
```

```
## Warning: Removed 16 rows containing non-finite outside the scale range
## (`stat_boxplot()`).
```

```
## Warning: Removed 16 rows containing missing values or values outside the scale range
## (`geom_point()`).
```

```
## Warning: Removed 7 rows containing non-finite outside the scale range
## (`stat_ydensity()`).
```

```
## Warning: Removed 7 rows containing non-finite outside the scale range
## (`stat_boxplot()`).
```

```
## Warning: Removed 7 rows containing missing values or values outside the scale range
## (`geom_point()`).
```

```
## Warning: Removed 9 rows containing non-finite outside the scale range
## (`stat_ydensity()`).
```

```
## Warning: Removed 9 rows containing non-finite outside the scale range
## (`stat_boxplot()`).
```

```
## Warning: Removed 9 rows containing missing values or values outside the scale range
## (`geom_point()`).
```

Generate random normal data for reference

```
set.seed(123) #generating random normal data for reference

MyMin <- min(Uintatheres$parPro, na.rm = T)
MyMax <- max(Uintatheres$parPro, na.rm = T)
MyMean <- mean(Uintatheres$parPro, na.rm = T)
MyVar <- var(Uintatheres$parPro, na.rm = T)
MySD <- sqrt(MyVar)
MyNorm <- rnorm(19, mean = MyMean, sd = MySD)
MyData <- data.frame(random = 1:19)
MyData$random <- MyNorm

random <- ggplot(data = MyData,
                 mapping = aes(x="", y= `random` )) +
  geom_violin( fill = "gray80", trim = F) + geom_point() + geom_boxplot(width = 0.1, fill = "gray70") +
  labs(y = "Length (cm)", x="", title = "Random Normal Data")

#plot with random normal data

grid.arrange(dims[[1]], dims[[2]], dims[[3]], dims[[4]], dims[[5]], dims[[6]], dims[[7]], dims[[8]], dims[[9]], random,
             ncol = 2, nrow = 5)
```

```
## Warning: Removed 2 rows containing non-finite outside the scale range
## (`stat_ydensity()`).
```

```
## Warning: Removed 2 rows containing non-finite outside the scale range
## (`stat_boxplot()`).
```

```
## Warning: Removed 2 rows containing missing values or values outside the scale range
## (`geom_point()`).
```

```
## Warning: Removed 7 rows containing non-finite outside the scale range
## (`stat_ydensity()`).
```

```
## Warning: Removed 7 rows containing non-finite outside the scale range
## (`stat_boxplot()`).
```

```
## Warning: Removed 7 rows containing missing values or values outside the scale range
## (`geom_point()`).
```

```
## Warning: Removed 12 rows containing non-finite outside the scale range
## (`stat_ydensity()`).
```

```
## Warning: Removed 12 rows containing non-finite outside the scale range
## (`stat_boxplot()`).
```

```
## Warning: Removed 12 rows containing missing values or values outside the scale range
## (`geom_point()`).
```

```
## Warning: Removed 7 rows containing non-finite outside the scale range
## (`stat_ydensity()`).
```

```
## Warning: Removed 7 rows containing non-finite outside the scale range
## (`stat_boxplot()`).
```

```
## Warning: Removed 7 rows containing missing values or values outside the scale range
## (`geom_point()`).
```

```
## Warning: Removed 7 rows containing non-finite outside the scale range
## (`stat_ydensity()`).
```

```
## Warning: Removed 7 rows containing non-finite outside the scale range
## (`stat_boxplot()`).
```

```
## Warning: Removed 7 rows containing missing values or values outside the scale range
## (`geom_point()`).
```

```
## Warning: Removed 5 rows containing non-finite outside the scale range
## (`stat_ydensity()`).
```

```
## Warning: Removed 5 rows containing non-finite outside the scale range
## (`stat_boxplot()`).
```

```
## Warning: Removed 5 rows containing missing values or values outside the scale range
## (`geom_point()`).
```

```
## Warning: Removed 16 rows containing non-finite outside the scale range
## (`stat_ydensity()`).
```

```
## Warning: Removed 16 rows containing non-finite outside the scale range
## (`stat_boxplot()`).
```

```
## Warning: Removed 16 rows containing missing values or values outside the scale range
## (`geom_point()`).
```

```
## Warning: Removed 7 rows containing non-finite outside the scale range
## (`stat_ydensity()`).
```

```
## Warning: Removed 7 rows containing non-finite outside the scale range
## (`stat_boxplot()`).
```

```
## Warning: Removed 7 rows containing missing values or values outside the scale range
## (`geom_point()`).
```

```
## Warning: Removed 9 rows containing non-finite outside the scale range
## (`stat_ydensity()`).
```

```
## Warning: Removed 9 rows containing non-finite outside the scale range
## (`stat_boxplot()`).
```

```
## Warning: Removed 9 rows containing missing values or values outside the scale range
## (`geom_point()`).
```

Perform Mann-Whitney U tests to determine if purported female and
male specimens are statistically distinguishable

```
UintatheresSexed <- read.csv("Uintathere_Measurements.csv", check.names = F) #load second dataframe
UintatheresSexed <- UintatheresSexed[UintatheresSexed$Specimen != "YPM VP 011039L",] #remove damaged view of YPM 11039
rownames(UintatheresSexed) <- UintatheresSexed[,1] #rename rows as specimen numbers
uinFem <- UintatheresSexed$Specimen[UintatheresSexed$sex=="F"]
uinMale <- UintatheresSexed$Specimen[UintatheresSexed$sex=="M"]
uinNB <- UintatheresSexed$Specimen[UintatheresSexed$sex=="?"]
UintatheresSexed <- UintatheresSexed[,4:14] #remove helper variables
UintatheresSexed <- UintatheresSexed[,-7] #remove old variable
UintatheresSexed <- UintatheresSexed[UintatheresSexed$sex != "?",] #only include specimens for which sex has been suggested
UintatheresSexed$sex <- as.factor(UintatheresSexed$sex)
#Uintatheres[1,]$alvCan <- NA #This canine is potentially pathologic so it's inclusion is questionable

wilcox.test(nasOcc ~ sex,
            data = UintatheresSexed, 
            alternative = "less")
```

```
## 
##  Wilcoxon rank sum exact test
## 
## data:  nasOcc by sex
## W = 3, p-value = 0.2
## alternative hypothesis: true location shift is less than 0
```

```
wilcox.test(nasOcc ~ sex,
            data = UintatheresSexed, 
            alternative = "less")
```

```
## 
##  Wilcoxon rank sum exact test
## 
## data:  nasOcc by sex
## W = 3, p-value = 0.2
## alternative hypothesis: true location shift is less than 0
```

```
wilcox.test(nasCon ~ sex,
            data = UintatheresSexed, 
            alternative = "less")
```

```
## 
##  Wilcoxon rank sum exact test
## 
## data:  nasCon by sex
## W = 3, p-value = 0.6
## alternative hypothesis: true location shift is less than 0
```

```
wilcox.test(preCon ~ sex,
            data = UintatheresSexed, 
            alternative = "less")
```

```
## 
##  Wilcoxon rank sum exact test
## 
## data:  preCon by sex
## W = 4, p-value = 0.8
## alternative hypothesis: true location shift is less than 0
```

```
wilcox.test(occCon ~ sex,
            data = UintatheresSexed, 
            alternative = "less")
```

```
## 
##  Wilcoxon rank sum exact test
## 
## data:  occCon by sex
## W = 4, p-value = 0.8
## alternative hypothesis: true location shift is less than 0
```

```
wilcox.test(parPro ~ sex,
            data = UintatheresSexed, 
            alternative = "less")
```

```
## 
##  Wilcoxon rank sum exact test
## 
## data:  parPro by sex
## W = 0, p-value = 0.05
## alternative hypothesis: true location shift is less than 0
```

```
wilcox.test(maxAlv ~ sex,
            data = UintatheresSexed, 
            alternative = "less")
```

```
## 
##  Wilcoxon rank sum exact test
## 
## data:  maxAlv by sex
## W = 2, p-value = 0.1143
## alternative hypothesis: true location shift is less than 0
```

```
wilcox.test(alvCan ~ sex,
            data = UintatheresSexed, 
            alternative = "less")
```

```
## 
##  Wilcoxon rank sum exact test
## 
## data:  alvCan by sex
## W = 0, p-value = 0.25
## alternative hypothesis: true location shift is less than 0
```

```
wilcox.test(diaLen ~ sex,
            data = UintatheresSexed, 
            alternative = "less")
```

```
## 
##  Wilcoxon rank sum exact test
## 
## data:  diaLen by sex
## W = 11, p-value = 0.9714
## alternative hypothesis: true location shift is less than 0
```

```
wilcox.test(canWid ~ sex,
            data = UintatheresSexed, 
            alternative = "less")
```

```
## 
##  Wilcoxon rank sum exact test
## 
## data:  canWid by sex
## W = 0, p-value = 0.06667
## alternative hypothesis: true location shift is less than 0
```

Mixture modeling

```
mixtureModels <- data.frame(
  metric = character(),
  numberComponents = numeric(),
  BIC = numeric()
)

for (i in 1:length(colnames(Uintatheres))) {
  mixMod <- Mclust(Uintatheres[,i][!is.na(Uintatheres[,i])])
  assign(paste(colnames(Uintatheres)[i], "mixMod"), mixMod)
  mixtureModels[i,1] <- colnames(Uintatheres)[i]
  mixtureModels[i,2] <- mixMod$G
  mixtureModels[i,3] <- mixMod$bic
}
```

PART 2: Multivariate analysis

First, missing data must be imputed for PCA (Impute if 3 or fewer
missing, otherwise, remove specimen)

```
aggr(Uintatheres)
```

```
uinTrim <- Uintatheres[rowSums(is.na(Uintatheres)) < 5,]
aggr(uinTrim)
```

```
uinScale <- scale(uinTrim)


ut.ncp <- estim_ncpPCA(uinScale, method.cv= "Kfold")
```

```
##   |                                                                              |                                                                      |   0%  |                                                                              |=                                                                     |   1%  |                                                                              |=                                                                     |   2%  |                                                                              |==                                                                    |   3%  |                                                                              |===                                                                   |   4%  |                                                                              |====                                                                  |   5%  |                                                                              |====                                                                  |   6%  |                                                                              |=====                                                                 |   7%  |                                                                              |======                                                                |   8%  |                                                                              |======                                                                |   9%  |                                                                              |=======                                                               |  10%  |                                                                              |========                                                              |  11%  |                                                                              |========                                                              |  12%  |                                                                              |=========                                                             |  13%  |                                                                              |==========                                                            |  14%  |                                                                              |===========                                                           |  15%  |                                                                              |===========                                                           |  16%  |                                                                              |============                                                          |  17%  |                                                                              |=============                                                         |  18%  |                                                                              |=============                                                         |  19%  |                                                                              |==============                                                        |  20%  |                                                                              |===============                                                       |  21%  |                                                                              |================                                                      |  22%  |                                                                              |================                                                      |  23%  |                                                                              |=================                                                     |  24%  |                                                                              |==================                                                    |  25%  |                                                                              |==================                                                    |  26%  |                                                                              |===================                                                   |  27%  |                                                                              |====================                                                  |  28%  |                                                                              |=====================                                                 |  29%  |                                                                              |=====================                                                 |  30%  |                                                                              |======================                                                |  31%  |                                                                              |=======================                                               |  32%  |                                                                              |=======================                                               |  33%  |                                                                              |========================                                              |  34%  |                                                                              |=========================                                             |  35%  |                                                                              |=========================                                             |  36%  |                                                                              |==========================                                            |  37%  |                                                                              |===========================                                           |  38%  |                                                                              |============================                                          |  39%  |                                                                              |============================                                          |  40%  |                                                                              |=============================                                         |  41%  |                                                                              |==============================                                        |  42%  |                                                                              |==============================                                        |  43%  |                                                                              |===============================                                       |  44%  |                                                                              |================================                                      |  45%  |                                                                              |=================================                                     |  46%  |                                                                              |=================================                                     |  47%  |                                                                              |==================================                                    |  48%  |                                                                              |===================================                                   |  49%  |                                                                              |===================================                                   |  51%  |                                                                              |====================================                                  |  52%  |                                                                              |=====================================                                 |  53%  |                                                                              |=====================================                                 |  54%  |                                                                              |======================================                                |  55%  |                                                                              |=======================================                               |  56%  |                                                                              |========================================                              |  57%  |                                                                              |========================================                              |  58%  |                                                                              |=========================================                             |  59%  |                                                                              |==========================================                            |  60%  |                                                                              |==========================================                            |  61%  |                                                                              |===========================================                           |  62%  |                                                                              |============================================                          |  63%  |                                                                              |=============================================                         |  64%  |                                                                              |=============================================                         |  65%  |                                                                              |==============================================                        |  66%  |                                                                              |===============================================                       |  67%  |                                                                              |===============================================                       |  68%  |                                                                              |================================================                      |  69%  |                                                                              |=================================================                     |  70%  |                                                                              |=================================================                     |  71%  |                                                                              |==================================================                    |  72%  |                                                                              |===================================================                   |  73%  |                                                                              |====================================================                  |  74%  |                                                                              |====================================================                  |  75%  |                                                                              |=====================================================                 |  76%  |                                                                              |======================================================                |  77%  |                                                                              |======================================================                |  78%  |                                                                              |=======================================================               |  79%  |                                                                              |========================================================              |  80%  |                                                                              |=========================================================             |  81%  |                                                                              |=========================================================             |  82%  |                                                                              |==========================================================            |  83%  |                                                                              |===========================================================           |  84%  |                                                                              |===========================================================           |  85%  |                                                                              |============================================================          |  86%  |                                                                              |=============================================================         |  87%  |                                                                              |==============================================================        |  88%  |                                                                              |==============================================================        |  89%  |                                                                              |===============================================================       |  90%  |                                                                              |================================================================      |  91%  |                                                                              |================================================================      |  92%  |                                                                              |=================================================================     |  93%  |                                                                              |==================================================================    |  94%  |                                                                              |==================================================================    |  95%  |                                                                              |===================================================================   |  96%  |                                                                              |====================================================================  |  97%  |                                                                              |===================================================================== |  98%  |                                                                              |===================================================================== |  99%  |                                                                              |======================================================================| 100%
```

```
plot(ut.ncp$criterion~names(ut.ncp$criterion),xlab="number of dimensions", 
     ylab ="ncp criterion")
```

```
ncp <- as.numeric(ut.ncp$ncp[1]) 

uinImp <- imputePCA(uinScale, ncp = ncp)
uinImp <- uinImp$completeObs
uinImp <- as.data.frame(uinImp)
```

Hierarchical Clustering

```
uinSex <- uinImp
uinSex$sex <- NA

uinSex$sex <- ifelse(rownames(uinSex) %in% uinFem, "F", 
                     ifelse(rownames(uinSex) %in% uinMale, "M", "?"))

row_colors <- ifelse(uinSex$sex == "F", "red", 
                     ifelse(uinSex$sex == "M", "blue", "black"))

Heatmap(uinImp,
        name = "Z-score",
        cluster_rows = TRUE,
        cluster_columns = TRUE,
        clustering_distance_rows = "euclidean",
        clustering_distance_columns = "euclidean",
        clustering_method_rows = "complete",
        clustering_method_columns = "complete",
        column_names_rot = 65,
        column_names_gp = gpar(fontsize = 10),
        row_names_gp = gpar(col = row_colors, fontsize = 15),
        row_dend_width = unit(75, "points")
)
```

```
## Warning: The input is a data frame-like object, convert it to a matrix.
```

```
###ALTERNATIVE CLUSTERING WITH BOOTSTRAP SUPPORT VALUES

bootMap <- pvclust(t(uinImp), method.dist="euclidean", method.hclust="complete", nboot=1000)
```

```
## Bootstrap (r = 0.44)... Done.
## Bootstrap (r = 0.56)... Done.
## Bootstrap (r = 0.67)... Done.
## Bootstrap (r = 0.78)... Done.
## Bootstrap (r = 0.89)... Done.
## Bootstrap (r = 1.0)... Done.
## Bootstrap (r = 1.11)... Done.
## Bootstrap (r = 1.22)... Done.
## Bootstrap (r = 1.33)... Done.
```

```
plot(bootMap)
pvrect(bootMap, alpha=0.95)
```

```
par(mar = c(4.5, 2, 2, 2))
plot(bootMap, labels = FALSE, hang = -5, main = "Hierarchical Clustering of Uintathere Skulls", print.num = F,
     xlab = "", sub="")
tipLabels <- bootMap$hclust$labels[bootMap$hclust$order]
tipColors <- ifelse(bootMap$hclust$labels[bootMap$hclust$order] %in% uinFem, "red",
                    ifelse(bootMap$hclust$labels[bootMap$hclust$order] %in% uinMale, "blue", "black"))
text(x = 1:length(tipLabels), y = -0.02, labels = tipLabels, col = tipColors,
     srt = 90,adj = 1,xpd = TRUE, cex = 0.75, font = 2)
```

```
dev.off()
```

```
## null device 
##           1
```

```
uinImp$sex <- uinSex$sex
uinImp$sex <- as.factor(uinImp$sex)
```

PCA

```
uinImpPCA <- PCA(uinImp[,1:9], scale.unit=F)
```

```
# write.csv(uinImpPCA$eig, "eigVals.csv")
# write.csv(uinImpPCA$var$coord, "pcLoad.csv")
# write.csv(uinImpPCA$var$cos2, "pcContVar.csv")
# write.csv(uinImpPCA$var$contrib, "varContPc.csv")

fviz_pca_var(uinImpPCA, col.var = "contrib")
```

```
fviz_pca_ind(uinImpPCA)
```

```
fviz_pca_biplot(uinImpPCA)
```

```
#Designate purported males and females to be separated into different hulls in PCA

coords <- uinImpPCA$ind$coord
coords <- as.data.frame(coords)
isFem <- rownames(coords) %in% uinFem
coords$Fem <- isFem
coords$sex <- uinSex$sex
coords$sex <- as.factor(coords$sex)

hull_sex <- coords %>%
  group_by(sex) %>%
  slice(chull(Dim.1, Dim.2))

hull_sex$sex <- as.factor(hull_sex$sex)
hull_sex$Sex <- hull_sex$sex

coords$Sex <- as.factor(coords$sex)

MyPlot <- ggplot(coords, aes(Dim.1, Dim.2)) + geom_point(shape = 21)
MyPlot + aes(fill = factor(sex)) + geom_polygon(data = hull_sex, alpha = 0.5) +
  labs(x = "PC1", y = "PC2", title = "PCA of Uintatherium Skulls")+ geom_vline(xintercept = 0) + geom_hline(yintercept = 0) +
  theme(legend.title = element_blank()) +
  geom_label_repel(aes(label = rownames(coords))) +
  scale_fill_manual(values = c("#B8B8B8", "#F8766D", "#619CFF"))
```

```
MyPlot +
  geom_polygon(data = hull_sex, aes(group = sex, fill = sex), alpha = 0.5) +
  labs(x = "PC1", y = "PC2", title = "PCA of Uintatherium Skulls") +
  geom_vline(xintercept = 0) +
  geom_hline(yintercept = 0) +
  geom_label_repel(aes(label = rownames(coords))) +
  scale_fill_manual(
    name = "Sex",  # legend title
    values = c(
      "F" = "#F8766D",
      "M" = "#619CFF",
      "?" = "#B8B8B8"
    )
  )
```

Determine number of clusters data appears to form

```
gapStat <- fviz_nbclust(uinImp[,1:9], kmeans, k.max = 8, method = "gap_stat")
fviz_nbclust(uinImp[,1:9], kmeans, k.max = 8, method = "gap_stat")
```

```
gapStat <- fviz_nbclust(uinImp[,1:9], kmeans, k.max = 17, method = "gap_stat")
wss <- fviz_nbclust(uinImp[,1:9], kmeans, k.max = 17, method = "wss")
silh <- fviz_nbclust(uinImp[,1:9], kmeans, k.max = 17, method = "silhouette")

wss / silh / gapStat
```

Use k-means clustering to form two most favorable clusters

```
km.UinTrimImp <- kmeans(uinImp[,1:9], centers = 2)
UintatheresClustered <- uinImp
clustVec <- km.UinTrimImp$cluster
UintatheresClustered <- as.data.frame(UintatheresClustered)
UintatheresClustered$cluster = clustVec
UintatheresClustered$cluster <- as.factor(UintatheresClustered$cluster)

ClusteredPCA <- PCA(UintatheresClustered[,1:9])
```

```
clusterCoords <-ClusteredPCA$ind$coord
clusterCoords <- as.data.frame(coords)
clusterCoords <- clusterCoords[,-c(6,7)]
clusterCoords$Cluster <- UintatheresClustered$cluster

hull_cluster <- clusterCoords %>%
  group_by(Cluster) %>%
  slice(chull(Dim.1, Dim.2))

MyClusterPlot <- ggplot(clusterCoords, aes(x = Dim.1, y = Dim.2)) + geom_point(shape = 21)

MyClusterPlot + aes(fill = Cluster) + geom_polygon(data = hull_cluster, alpha = 0.5) +
  labs(x = "PC1", y = "PC2", colour = "Cluster", title = "PCA of Uintatherium Skulls") + 
  geom_vline(xintercept = 0) + geom_hline(yintercept = 0) +
  geom_label_repel(aes(label = rownames(clusterCoords))) +
  scale_fill_manual(values = c("#619CFF", "#F8766D"))
```

```
## Ignoring unknown labels:
## • colour : "Cluster"
```

```
hull_sex$sex <- as.factor(hull_sex$sex)
hull_sex$Sex <- hull_sex$sex
```

Plot purported sex and cluster on the same PCA

```
MyClusterPlot +
  geom_polygon(
    data = hull_cluster,
    aes(x = Dim.1, y = Dim.2, group = Cluster, fill = factor(Cluster)),
    alpha = 0.25,
    linewidth = 0.3,
    colour = "gray25",
    inherit.aes = FALSE) +
  scale_fill_manual(name = "Cluster", values = c("1" = "#4DB6AC", "2" = "#D4A72C")) +
  new_scale_fill() +
  geom_point(aes(fill = Sex), shape = 21, size = 3) +
  geom_label_repel(aes(label = rownames(clusterCoords), fill = Sex),colour = "black" ) +
  scale_fill_manual(values = c("?" = "#B8B8B8","F" = "#F8766D","M" = "#619CFF")) +
  labs(x = "PC1 (60.05%)", y = "PC2 (19.61%)", title = "PCA of Uintathere Skulls") +
  geom_vline(xintercept = 0) +
  geom_hline(yintercept = 0)
```

Use PCA and PERMANOVA to determine if purported females and males
consistently differ across metrics

```
### FIRST NEED TO IMPUTE MISSING DATA WITH ONLY SEXED SKULLS

UintatheresSexedScaled <- scale(UintatheresSexed[,1:9])
UintatheresSexedScaled <- as.data.frame(UintatheresSexedScaled)

ut.ncp <- estim_ncpPCA(UintatheresSexedScaled, method.cv= "Kfold")
```

```
##   |                                                                              |                                                                      |   0%  |                                                                              |=                                                                     |   1%  |                                                                              |=                                                                     |   2%  |                                                                              |==                                                                    |   3%  |                                                                              |===                                                                   |   4%  |                                                                              |====                                                                  |   5%  |                                                                              |====                                                                  |   6%  |                                                                              |=====                                                                 |   7%  |                                                                              |======                                                                |   8%  |                                                                              |======                                                                |   9%  |                                                                              |=======                                                               |  10%  |                                                                              |========                                                              |  11%  |                                                                              |========                                                              |  12%  |                                                                              |=========                                                             |  13%  |                                                                              |==========                                                            |  14%  |                                                                              |===========                                                           |  15%  |                                                                              |===========                                                           |  16%  |                                                                              |============                                                          |  17%  |                                                                              |=============                                                         |  18%  |                                                                              |=============                                                         |  19%  |                                                                              |==============                                                        |  20%  |                                                                              |===============                                                       |  21%  |                                                                              |================                                                      |  22%  |                                                                              |================                                                      |  23%  |                                                                              |=================                                                     |  24%  |                                                                              |==================                                                    |  25%  |                                                                              |==================                                                    |  26%  |                                                                              |===================                                                   |  27%  |                                                                              |====================                                                  |  28%  |                                                                              |=====================                                                 |  29%  |                                                                              |=====================                                                 |  30%  |                                                                              |======================                                                |  31%  |                                                                              |=======================                                               |  32%  |                                                                              |=======================                                               |  33%  |                                                                              |========================                                              |  34%  |                                                                              |=========================                                             |  35%  |                                                                              |=========================                                             |  36%  |                                                                              |==========================                                            |  37%  |                                                                              |===========================                                           |  38%  |                                                                              |============================                                          |  39%  |                                                                              |============================                                          |  40%  |                                                                              |=============================                                         |  41%  |                                                                              |==============================                                        |  42%  |                                                                              |==============================                                        |  43%  |                                                                              |===============================                                       |  44%  |                                                                              |================================                                      |  45%  |                                                                              |=================================                                     |  46%  |                                                                              |=================================                                     |  47%  |                                                                              |==================================                                    |  48%  |                                                                              |===================================                                   |  49%  |                                                                              |===================================                                   |  51%  |                                                                              |====================================                                  |  52%  |                                                                              |=====================================                                 |  53%  |                                                                              |=====================================                                 |  54%  |                                                                              |======================================                                |  55%  |                                                                              |=======================================                               |  56%  |                                                                              |========================================                              |  57%  |                                                                              |========================================                              |  58%  |                                                                              |=========================================                             |  59%  |                                                                              |==========================================                            |  60%  |                                                                              |==========================================                            |  61%  |                                                                              |===========================================                           |  62%  |                                                                              |============================================                          |  63%  |                                                                              |=============================================                         |  64%  |                                                                              |=============================================                         |  65%  |                                                                              |==============================================                        |  66%  |                                                                              |===============================================                       |  67%  |                                                                              |===============================================                       |  68%  |                                                                              |================================================                      |  69%  |                                                                              |=================================================                     |  70%  |                                                                              |=================================================                     |  71%  |                                                                              |==================================================                    |  72%  |                                                                              |===================================================                   |  73%  |                                                                              |====================================================                  |  74%  |                                                                              |====================================================                  |  75%  |                                                                              |=====================================================                 |  76%  |                                                                              |======================================================                |  77%  |                                                                              |======================================================                |  78%  |                                                                              |=======================================================               |  79%
```

```
## Warning in impute(X, ncp = ncp, scale = scale, method = method, ind.sup =
## ind.sup, : Stopped after 1000 iterations
```

```
##   |                                                                              |========================================================              |  80%  |                                                                              |=========================================================             |  81%  |                                                                              |=========================================================             |  82%  |                                                                              |==========================================================            |  83%  |                                                                              |===========================================================           |  84%  |                                                                              |===========================================================           |  85%
```

```
## Warning in impute(X, ncp = ncp, scale = scale, method = method, ind.sup =
## ind.sup, : Stopped after 1000 iterations
```

```
##   |                                                                              |============================================================          |  86%  |                                                                              |=============================================================         |  87%  |                                                                              |==============================================================        |  88%  |                                                                              |==============================================================        |  89%  |                                                                              |===============================================================       |  90%  |                                                                              |================================================================      |  91%  |                                                                              |================================================================      |  92%  |                                                                              |=================================================================     |  93%  |                                                                              |==================================================================    |  94%  |                                                                              |==================================================================    |  95%  |                                                                              |===================================================================   |  96%  |                                                                              |====================================================================  |  97%  |                                                                              |===================================================================== |  98%  |                                                                              |===================================================================== |  99%  |                                                                              |======================================================================| 100%
```

```
ncp <- as.numeric(ut.ncp$ncp[1]) 

uintatheresSexedImputed <- imputePCA(UintatheresSexedScaled, ncp = ncp)
uintatheresSexedImputed <- uintatheresSexedImputed$completeObs
uintatheresSexedImputed <- as.data.frame(uintatheresSexedImputed)
uintatheresSexedImputed$sex <- UintatheresSexed$sex

### PCA

sexedPCA <- PCA(uintatheresSexedImputed[,1:9], scale.unit=F)
```

```
###########################RUNNING PERMANOVA TO SEE IF PC SCORE IS AFFECTED BY SEX

sexedPCA$eig
```

```
##        eigenvalue percentage of variance cumulative percentage of variance
## comp 1 3.77689591             59.0113085                          59.01131
## comp 2 2.04505305             31.9524973                          90.96381
## comp 3 0.35783230              5.5908748                          96.55468
## comp 4 0.16039098              2.5059948                          99.06068
## comp 5 0.05076979              0.7932417                          99.85392
```

```
sigCols <- sexedPCA$ind$coord[,1:3]
sigCols <- as.data.frame(sigCols)
sigCols$sex <- NA
sigCols$sex <- ifelse(rownames(sigCols) %in% uinFem, "F", 
                      ifelse(rownames(sigCols) %in% uinMale, "M", "?"))

sigCols$sex <- as.factor(sigCols$sex)
adonis2(sigCols[,c("Dim.1", "Dim.2", "Dim.3")] ~ sex, data = sigCols, method = "euc")
```

```
## Permutation test for adonis under reduced model
## Permutation: free
## Number of permutations: 999
## 
## adonis2(formula = sigCols[, c("Dim.1", "Dim.2", "Dim.3")] ~ sex, data = sigCols, method = "euc")
##          Df SumOfSqs      R2      F Pr(>F)  
## Model     1   15.386 0.31123 2.7111  0.063 .
## Residual  6   34.052 0.68877                
## Total     7   49.438 1.00000                
## ---
## Signif. codes:  0 '***' 0.001 '**' 0.01 '*' 0.05 '.' 0.1 ' ' 1
```

Lastly, estimate the degree of dimorphism in each metric using finite
mixture analysis and the method of means

```
estimateTable <- matrix(nrow = 9, ncol = 2)
rownames(estimateTable) <- colnames(Uintatheres)
colnames(estimateTable) <- c("FMA", "MM")
estimateTable <- as.data.frame(estimateTable)

pearsonTable <- read.csv("pearsonTable.csv")

for (i in 1:nrow(estimateTable)) {
  metric <- na.omit(Uintatheres[,i])
  mean <- mean(metric)
  OR <- max(metric) - min(metric)
  n <- length(metric)
  k <- pearsonTable$stDevsInRange[pearsonTable$SampleSize == n]
  sigmaTot <- OR/k
  expSub <- k*(2^.5) #expected number of subpop st devs in total OR
  twoSubPercent <- 2/expSub #portion of OR expected to be occupied by 2 sigmaSub
  oneSubPercent <- twoSubPercent/2 #portion of OR expected to be occupied by 1 sigmaSub
  distanceToSub <- oneSubPercent*OR #numeric value of one sigmaSub <- distance from overall mean to mean of each subpop
  bigMean <- mean + distanceToSub
  smallMean <- mean - distanceToSub
  fmaEstimate <- bigMean/smallMean
  estimateTable$FMA[i] <- fmaEstimate
  bigs <- metric[metric > mean(metric)]
  bigMean <- mean(bigs)
  smalls <- metric[metric < mean(metric)]
  smallMean <- mean(smalls)
  mmEstimate <- bigMean/smallMean
  estimateTable$MM[i] <- mmEstimate
}
```
