# Supplementary material for: Linear morphometrics fail to support strong sexual dimorphism in Uintatherium anceps
Source: PLoS One. 2026 Oct 7;21(10):e0345236. doi: 10.1371/journal.pone.0345236 (PMC13645082; doi:10.1371/journal.pone.0345236)
Supplement: Supplementary file 10 [file pone.0345236.s024.html]

Bison Analyses


# Bison Analyses

#### 2026-07-24

Set wd and load packages

```
#setwd("")

library(dplyr)
```

```
## 
## Attaching package: 'dplyr'
```

```
## The following objects are masked from 'package:stats':
## 
##     filter, lag
```

```
## The following objects are masked from 'package:base':
## 
##     intersect, setdiff, setequal, union
```

```
library(diptest)
library(Amelia)
```

```
## Loading required package: Rcpp
```

```
## ## 
## ## Amelia II: Multiple Imputation
## ## (Version 1.8.3, built: 2024-11-07)
## ## Copyright (C) 2005-2026 James Honaker, Gary King and Matthew Blackwell
## ## Refer to http://gking.harvard.edu/amelia/ for more information
## ##
```

```
library(cluster)
library(ggfortify)
```

```
## Loading required package: ggplot2
```

```
library(ggplot2)
library(ggforce)
library(factoextra)
```

```
## Welcome to factoextra!
```

```
## Want to learn more? See two factoextra-related books at https://www.datanovia.com/en/product/practical-guide-to-principal-component-methods-in-r/
```

```
library(FactoMineR)
library(missMDA)
library(VIM)
```

```
## Loading required package: colorspace
```

```
## Loading required package: grid
```

```
## Registered S3 method overwritten by 'car':
##   method           from
##   na.action.merMod lme4
```

```
## VIM is ready to use.
```

```
## Suggestions and bug-reports can be submitted at: https://github.com/statistikat/VIM/issues
```

```
## 
## Attaching package: 'VIM'
```

```
## The following object is masked from 'package:datasets':
## 
##     sleep
```

```
library(cowplot)
library(plyr)
```

```
## ------------------------------------------------------------------------------
```

```
## You have loaded plyr after dplyr - this is likely to cause problems.
## If you need functions from both plyr and dplyr, please load plyr first, then dplyr:
## library(plyr); library(dplyr)
```

```
## ------------------------------------------------------------------------------
```

```
## 
## Attaching package: 'plyr'
```

```
## The following objects are masked from 'package:dplyr':
## 
##     arrange, count, desc, mutate, rename, summarise, summarize
```

```
library(ggrepel)
library(gridExtra)
```

```
## 
## Attaching package: 'gridExtra'
```

```
## The following object is masked from 'package:dplyr':
## 
##     combine
```

```
library(mclust)
```

```
## Package 'mclust' version 6.1.3
## Type 'citation("mclust")' for citing this R package in publications.
```

```
## 
## Attaching package: 'mclust'
```

```
## The following object is masked from 'package:plyr':
## 
##     count
```

```
## The following object is masked from 'package:VIM':
## 
##     diabetes
```

```
## The following object is masked from 'package:dplyr':
## 
##     count
```

```
library(GGally)
library(BayesFactor)
```

```
## Loading required package: coda
```

```
## Loading required package: Matrix
```

```
## ************
## Welcome to BayesFactor 0.9.12-4.8. If you have questions, please contact Richard Morey (richarddmorey@gmail.com).
## 
## Type BFManual() to open the manual.
## ************
```

```
library(pheatmap)
library(ComplexHeatmap)
```

```
## ========================================
## ComplexHeatmap version 2.24.1
## Bioconductor page: http://bioconductor.org/packages/ComplexHeatmap/
## Github page: https://github.com/jokergoo/ComplexHeatmap
## Documentation: http://jokergoo.github.io/ComplexHeatmap-reference
## 
## If you use it in published research, please cite either one:
## - Gu, Z. Complex Heatmap Visualization. iMeta 2022.
## - Gu, Z. Complex heatmaps reveal patterns and correlations in multidimensional 
##     genomic data. Bioinformatics 2016.
## 
## 
## The new InteractiveComplexHeatmap package can directly export static 
## complex heatmaps into an interactive Shiny app with zero effort. Have a try!
## 
## This message can be suppressed by:
##   suppressPackageStartupMessages(library(ComplexHeatmap))
## ========================================
## ! pheatmap() has been masked by ComplexHeatmap::pheatmap(). Most of the arguments
##    in the original pheatmap() are identically supported in the new function. You 
##    can still use the original function by explicitly calling pheatmap::pheatmap().
```

```
## 
## Attaching package: 'ComplexHeatmap'
```

```
## The following object is masked from 'package:pheatmap':
## 
##     pheatmap
```

```
## The following object is masked from 'package:VIM':
## 
##     prepare
```

```
library(circlize)
```

```
## ========================================
## circlize version 0.4.18
## CRAN page: https://cran.r-project.org/package=circlize
## Github page: https://github.com/jokergoo/circlize
## Documentation: https://jokergoo.github.io/circlize_book/book/
## 
## If you use it in published research, please cite:
## Gu, Z. circlize implements and enhances circular visualization
##   in R. Bioinformatics 2014.
## 
## This message can be suppressed by:
##   suppressPackageStartupMessages(library(circlize))
## ========================================
```

```
library(mixtools)
```

```
## mixtools package, version 2.0.0.1, Released 2022-12-04
## This package is based upon work supported by the National Science Foundation under Grant No. SES-0518772 and the Chan Zuckerberg Initiative: Essential Open Source Software for Science (Grant No. 2020-255193).
```

```
## 
## Attaching package: 'mixtools'
```

```
## The following object is masked from 'package:mclust':
## 
##     dmvnorm
```

```
## The following object is masked from 'package:grid':
## 
##     depth
```

```
library(patchwork)
```

```
## 
## Attaching package: 'patchwork'
```

```
## The following object is masked from 'package:cowplot':
## 
##     align_plots
```

```
library(EnvStats)
```

```
## 
## Attaching package: 'EnvStats'
```

```
## The following object is masked from 'package:Matrix':
## 
##     print
```

```
## The following objects are masked from 'package:stats':
## 
##     predict, predict.lm
```

```
## The following object is masked from 'package:base':
## 
##     print.default
```

```
library(dimorph)
library(effectsize)
library(pvclust)
library(ggnewscale)
library(MASS)
```

```
## 
## Attaching package: 'MASS'
```

```
## The following object is masked from 'package:EnvStats':
## 
##     boxcox
```

```
## The following object is masked from 'package:patchwork':
## 
##     area
```

```
## The following object is masked from 'package:dplyr':
## 
##     select
```

```
library(vegan)
```

```
## Loading required package: permute
```

```
## 
## Attaching package: 'vegan'
```

```
## The following object is masked from 'package:EnvStats':
## 
##     calibrate
```

Load and manipulate data

```
bison <- read.csv("Bison_Measurements.csv", check.names = F) #load dataframe
bisonAdults <- bison[-20, ] #remove 68350, specimen with adult dentition still erupting
rownames(bisonAdults) <- bisonAdults[,1] #rename rows as specimen numbers
adultSex <- bisonAdults[,2] #make a vector of sex for adult specimens
bisonAdults <- bisonAdults[,-c(1:4)] #remove helper variables
for (i in 1:ncol(bisonAdults)) {
  bisonAdults[,i] <- as.numeric(bisonAdults[,i])
}

str(bisonAdults)
```

```
## 'data.frame':    23 obs. of  9 variables:
##  $ tipFro: num  41.1 NA 43.9 47.8 NA ...
##  $ basFro: num  19.8 26.5 22.1 20.2 18.8 ...
##  $ ectEct: num  24.6 29.5 27.4 24.1 23.6 ...
##  $ maxMax: num  13.3 16.5 14.4 14.2 12.4 ...
##  $ proNas: num  23 27.8 25.1 24.4 23.5 ...
##  $ nasNuc: num  16.2 19.1 19 19.4 16 ...
##  $ basTip: num  12.9 20.5 12.9 16.5 NA ...
##  $ ectPro: num  29.8 34.7 31.8 32.9 29.7 ...
##  $ basWid: num  4.34 7.14 5.07 5.17 4.3 ...
```

```
bisonAdults <- bisonAdults[,-4] #remove maxMax to retain 8 variables

bisonSex <- bisonAdults
bisonSex$sex <- adultSex
```

PART 1: Generate histograms for each variable and test for normality
and unimodality

Setting up “Stats” dataframe to store values of various test
statistics of each measurement

```
columns <- c("Width between tips of frontal Horns", "Width bewteen bases of Frontal Horns", 
             "Width between exorbitale",
             "Length from prosthion to nasion", "Length from nasion to nuchal crest", 
             "Length of frontal horn from base to tip", "Length of skull from orbit to prosthion", 
             "Width of frontal horn at base")
NVariables <- length(columns)
Measurement <- vector()
n <- vector()
wStatistic <- vector()
pValue <- vector()
dipStatistic <- vector() 

#The 'width  between tips of frontal horns' measurement is not considered in final analyses

Stats <- data.frame(Measurement, n, wStatistic, pValue, dipStatistic, pValue)
```

RUN for-loop that plots histograms for each variable, runs
Shapiro-Wilk and Hartigan’s Dip Test, and stores these variables in
“Stats” dataframe.

```
par(mfrow = c(4,2))
par(mar=c(2,2,2,2))

for (i in 1:NVariables) {
  histi <- hist(bisonAdults[,i][!is.na(bisonAdults[,i])], main = paste(columns[i]),
                col = "skyblue1", xlab = paste(columns[i], "(cm)"))
  lengthi <- unique(bisonAdults[,i][!is.na(bisonAdults[,i])])
  xfit <- seq(min(bisonAdults[,i][!is.na(bisonAdults[,i])], na.rm = T), 
              max(bisonAdults[,i][!is.na(bisonAdults[,i])], na.rm = T), 
              length = length(lengthi)) 
  yfit <- dnorm(xfit, mean = mean(bisonAdults[,i][!is.na(bisonAdults[,i])], na.rm = T), 
                sd = sd(bisonAdults[,i][!is.na(bisonAdults[,i])], na.rm = T)) 
  yfit <- yfit * diff(histi$mids[1:2]) * length(bisonAdults[,i][!is.na(bisonAdults[,i])]) 
  lines(xfit, yfit, col = "red", lwd = 3)
  Stats[i,1] <- columns[i]
  Stats[i,2] <- length(bisonAdults[,i][!is.na(bisonAdults[,i])])
  Stats[i,3] <- shapiro.test(bisonAdults[,i])[1]
  Stats[i,4] <- shapiro.test(bisonAdults[,i])[2]
  Stats[i,5] <- dip.test(bisonAdults[,i])[1]
  Stats[i,6] <- dip.test(bisonAdults[,i])[2]
  mtext(paste("w-statistic: ", Stats[i,3], "; p-value: ", Stats[i,4], "\n",
              "dip-statistic: ", Stats[i,5], "; p-value: ", Stats[i,6],  sep = ""), 
        padj = .3, side = 4, cex = 0.45)
}
```

```
par(mfrow = c(1,1))
```

Generate Violin Plots for Each Measurement

```
par(mfrow = c(4,2))
par(mar=c(2,2,2,2))

dims <- list(length(colnames(bisonAdults)))
dims[[8]] <- ggplot(data = bisonAdults,
                  mapping = aes(x="", y= 'width between tips of frontal Horns' )) +
  geom_violin( fill = "gray80", trim = F) + geom_point() + geom_boxplot(width = 0.1, fill = "gray70") +
  labs(y = "Length (cm)", x="", title = "Width between tips of frontal Horns")


for (i in 1:length(colnames(bisonAdults))) {
  dims[[i]] <-  ggplot(data = bisonAdults,
                       mapping = aes(x="", y=.data[[colnames(bisonAdults)[i]]])) +
    geom_violin( fill = "gray80", trim = F) + geom_point() + geom_boxplot(width = 0.1, fill = "gray70") +
    labs(y = "Length (cm)", x="", title = colnames(bisonAdults)[i])
}

grid.arrange(dims[[1]], dims[[2]], dims[[3]], dims[[4]], dims[[5]], dims[[6]], dims[[7]], dims[[8]],
             ncol = 3, nrow = 3)
```

```
## Warning: Removed 12 rows containing non-finite outside the scale range
## (`stat_ydensity()`).
```

```
## Warning: Removed 12 rows containing non-finite outside the scale range
## (`stat_boxplot()`).
```

```
## Warning: Removed 12 rows containing missing values or values outside the scale range
## (`geom_point()`).
```

```
## Warning: Removed 3 rows containing non-finite outside the scale range
## (`stat_ydensity()`).
```

```
## Warning: Removed 3 rows containing non-finite outside the scale range
## (`stat_boxplot()`).
```

```
## Warning: Removed 3 rows containing missing values or values outside the scale range
## (`geom_point()`).
```

```
## Warning: Removed 5 rows containing non-finite outside the scale range
## (`stat_ydensity()`).
```

```
## Warning: Removed 5 rows containing non-finite outside the scale range
## (`stat_boxplot()`).
```

```
## Warning: Removed 5 rows containing missing values or values outside the scale range
## (`geom_point()`).
```

```
## Warning: Removed 3 rows containing non-finite outside the scale range
## (`stat_ydensity()`).
```

```
## Warning: Removed 3 rows containing non-finite outside the scale range
## (`stat_boxplot()`).
```

```
## Warning: Removed 3 rows containing missing values or values outside the scale range
## (`geom_point()`).
```

Generate random normal data for reference

```
set.seed(123)

MyMin <- min(bisonAdults$tipFro, na.rm = T)
MyMax <- max(bisonAdults$tipFro, na.rm = T)
MyMean <- mean(bisonAdults$tipFro, na.rm = T)
MyVar <- var(bisonAdults$tipFro, na.rm = T)
MySD <- sqrt(MyVar)
MyNorm <- rnorm(22, mean = MyMean, sd = MySD)
MyData <- data.frame(random = 1:22)
MyData$random <- MyNorm

random <- ggplot(data = MyData,
       mapping = aes(x="", y= `random` )) +
  geom_violin( fill = "gray80", trim = F) + geom_point() + geom_boxplot(width = 0.1, fill = "gray70") +
  labs(y = "Length (cm)", x="", title = "Random Normal Data")

#plot with random normal data

grid.arrange(dims[[2]], dims[[3]], dims[[4]], dims[[5]], dims[[6]], dims[[7]], dims[[8]], random,
             ncol = 2, nrow = 4)
```

```
## Warning: Removed 3 rows containing non-finite outside the scale range
## (`stat_ydensity()`).
```

```
## Warning: Removed 3 rows containing non-finite outside the scale range
## (`stat_boxplot()`).
```

```
## Warning: Removed 3 rows containing missing values or values outside the scale range
## (`geom_point()`).
```

```
## Warning: Removed 5 rows containing non-finite outside the scale range
## (`stat_ydensity()`).
```

```
## Warning: Removed 5 rows containing non-finite outside the scale range
## (`stat_boxplot()`).
```

```
## Warning: Removed 5 rows containing missing values or values outside the scale range
## (`geom_point()`).
```

```
## Warning: Removed 3 rows containing non-finite outside the scale range
## (`stat_ydensity()`).
```

```
## Warning: Removed 3 rows containing non-finite outside the scale range
## (`stat_boxplot()`).
```

```
## Warning: Removed 3 rows containing missing values or values outside the scale range
## (`geom_point()`).
```

Perform Mann-Whitney U tests to determine if purported female and
male specimens are statistically distinguishable

```
#########Load and manipulate bison data into a new dataframe, this time only retaining skulls with sex metadata

bisonSexed <- bisonSex[bisonSex$sex != "?",]
bisonSexed$sex <- as.factor(bisonSexed$sex)
set.seed(123) #randomly subsample to only eight specimens (four male and four female) to maintain equal sample with
#uintatheres
bisonMale <- bisonSexed[bisonSexed$sex == "M",]
bisonFem <- bisonSexed[bisonSexed$sex == "F",]
bisFem <- rownames(bisonFem)
bisonMale[sample((1:7), 4, replace = F), ]
```

```
##          tipFro   basFro   ectEct   proNas   nasNuc   basTip   ectPro   basWid
## 2498   40.99945 20.37102 23.59379 26.00468 15.19759 13.44560 32.11058 4.047532
## 140290 65.64165 28.81338 32.50509       NA 22.31396 23.91033       NA 7.711568
## 48115        NA 24.44283 29.04033 26.18923 16.23400       NA 33.22525 7.759264
## 92698  58.15846 26.81012 30.29437       NA 22.58518 18.73339       NA 8.400840
##        sex
## 2498     M
## 140290   M
## 48115    M
## 92698    M
```

```
bisMale <- rownames(bisonMale)
bisonFem[sample((1:9), 4, replace = F), ]
```

```
##          tipFro   basFro   ectEct   proNas   nasNuc   basTip   ectPro   basWid
## 21903  43.93227 22.11621 27.37292 25.13745 18.97459 12.87695 31.83566 5.074173
## 2495         NA 23.90488 25.52614 26.22277 17.48581 18.05268 33.07281 5.120214
## 141977 47.77667 20.17002 24.13626 24.44891 19.37498 16.45033 32.94181 5.168106
## 2501         NA 18.95102 23.87582 26.29317 15.21182       NA 32.24142 4.771462
##        sex
## 21903    F
## 2495     F
## 141977   F
## 2501     F
```

```
bisonSub <- rbind(bisonMale[sample((1:7), 4, replace = F), ], bisonFem[sample((1:9), 4, replace = F), ])

wilcox.test(basFro ~ sex,
            data = bisonSub,
            alternative = "less")
```

```
## 
##  Wilcoxon rank sum exact test
## 
## data:  basFro by sex
## W = 0, p-value = 0.01429
## alternative hypothesis: true location shift is less than 0
```

```
wilcox.test(ectEct ~ sex,
            data = bisonSub,
            alternative = "less")
```

```
## 
##  Wilcoxon rank sum exact test
## 
## data:  ectEct by sex
## W = 0, p-value = 0.01429
## alternative hypothesis: true location shift is less than 0
```

```
wilcox.test(proNas ~ sex,
            data = bisonSub,
            alternative = "less")
```

```
## 
##  Wilcoxon rank sum exact test
## 
## data:  proNas by sex
## W = 1, p-value = 0.05714
## alternative hypothesis: true location shift is less than 0
```

```
wilcox.test(nasNuc ~ sex,
            data = bisonSub,
            alternative = "less")
```

```
## 
##  Wilcoxon rank sum exact test
## 
## data:  nasNuc by sex
## W = 5, p-value = 0.2429
## alternative hypothesis: true location shift is less than 0
```

```
wilcox.test(basTip ~ sex,
            data = bisonSub,
            alternative = "less")
```

```
## 
##  Wilcoxon rank sum exact test
## 
## data:  basTip by sex
## W = 0, p-value = 0.05
## alternative hypothesis: true location shift is less than 0
```

```
wilcox.test(ectPro ~ sex,
            data = bisonSub,
            alternative = "less")
```

```
## 
##  Wilcoxon rank sum exact test
## 
## data:  ectPro by sex
## W = 0, p-value = 0.02857
## alternative hypothesis: true location shift is less than 0
```

```
wilcox.test(basWid ~ sex,
            data = bisonSub,
            alternative = "less")
```

```
## 
##  Wilcoxon rank sum exact test
## 
## data:  basWid by sex
## W = 0, p-value = 0.01429
## alternative hypothesis: true location shift is less than 0
```

Mixture modeling

```
mixtureModels <- data.frame(
  metric = character(),
  numberComponents = numeric(),
  BIC = numeric()
)

for (i in 1:length(colnames(bisonAdults))) {
  mixMod <- Mclust(bisonAdults[,i][!is.na(bisonAdults[,i])])
  assign(paste(colnames(bisonAdults)[i], "mixMod"), mixMod)
  mixtureModels[i,1] <- colnames(bisonAdults)[i]
  mixtureModels[i,2] <- mixMod$G
  mixtureModels[i,3] <- mixMod$bic
}

par(mfrow=c(1,1))
```

PART 2: Multivariate analysis

First, missing data must be imputed for PCA (Impute if 3 or fewer
missing, otherwise, remove specimen)

```
bisonAdults <- bisonAdults[,-1] #remove extra horn variable

bisScale <- scale(bisonAdults)

bis.ncp <- estim_ncpPCA(bisScale, method.cv= "Kfold")
```

```
##   |                                                                              |                                                                      |   0%  |                                                                              |=                                                                     |   1%  |                                                                              |=                                                                     |   2%  |                                                                              |==                                                                    |   3%  |                                                                              |===                                                                   |   4%  |                                                                              |====                                                                  |   5%  |                                                                              |====                                                                  |   6%  |                                                                              |=====                                                                 |   7%  |                                                                              |======                                                                |   8%  |                                                                              |======                                                                |   9%  |                                                                              |=======                                                               |  10%  |                                                                              |========                                                              |  11%  |                                                                              |========                                                              |  12%  |                                                                              |=========                                                             |  13%  |                                                                              |==========                                                            |  14%  |                                                                              |===========                                                           |  15%  |                                                                              |===========                                                           |  16%  |                                                                              |============                                                          |  17%  |                                                                              |=============                                                         |  18%  |                                                                              |=============                                                         |  19%  |                                                                              |==============                                                        |  20%  |                                                                              |===============                                                       |  21%  |                                                                              |================                                                      |  22%  |                                                                              |================                                                      |  23%  |                                                                              |=================                                                     |  24%  |                                                                              |==================                                                    |  25%  |                                                                              |==================                                                    |  26%  |                                                                              |===================                                                   |  27%  |                                                                              |====================                                                  |  28%  |                                                                              |=====================                                                 |  29%  |                                                                              |=====================                                                 |  30%  |                                                                              |======================                                                |  31%  |                                                                              |=======================                                               |  32%  |                                                                              |=======================                                               |  33%  |                                                                              |========================                                              |  34%  |                                                                              |=========================                                             |  35%  |                                                                              |=========================                                             |  36%  |                                                                              |==========================                                            |  37%  |                                                                              |===========================                                           |  38%  |                                                                              |============================                                          |  39%  |                                                                              |============================                                          |  40%  |                                                                              |=============================                                         |  41%  |                                                                              |==============================                                        |  42%  |                                                                              |==============================                                        |  43%  |                                                                              |===============================                                       |  44%  |                                                                              |================================                                      |  45%  |                                                                              |=================================                                     |  46%  |                                                                              |=================================                                     |  47%  |                                                                              |==================================                                    |  48%  |                                                                              |===================================                                   |  49%  |                                                                              |===================================                                   |  51%  |                                                                              |====================================                                  |  52%  |                                                                              |=====================================                                 |  53%  |                                                                              |=====================================                                 |  54%  |                                                                              |======================================                                |  55%  |                                                                              |=======================================                               |  56%  |                                                                              |========================================                              |  57%  |                                                                              |========================================                              |  58%  |                                                                              |=========================================                             |  59%  |                                                                              |==========================================                            |  60%  |                                                                              |==========================================                            |  61%  |                                                                              |===========================================                           |  62%  |                                                                              |============================================                          |  63%  |                                                                              |=============================================                         |  64%  |                                                                              |=============================================                         |  65%  |                                                                              |==============================================                        |  66%  |                                                                              |===============================================                       |  67%  |                                                                              |===============================================                       |  68%  |                                                                              |================================================                      |  69%  |                                                                              |=================================================                     |  70%  |                                                                              |=================================================                     |  71%  |                                                                              |==================================================                    |  72%  |                                                                              |===================================================                   |  73%  |                                                                              |====================================================                  |  74%  |                                                                              |====================================================                  |  75%  |                                                                              |=====================================================                 |  76%  |                                                                              |======================================================                |  77%  |                                                                              |======================================================                |  78%  |                                                                              |=======================================================               |  79%  |                                                                              |========================================================              |  80%  |                                                                              |=========================================================             |  81%  |                                                                              |=========================================================             |  82%  |                                                                              |==========================================================            |  83%  |                                                                              |===========================================================           |  84%  |                                                                              |===========================================================           |  85%  |                                                                              |============================================================          |  86%  |                                                                              |=============================================================         |  87%  |                                                                              |==============================================================        |  88%  |                                                                              |==============================================================        |  89%  |                                                                              |===============================================================       |  90%  |                                                                              |================================================================      |  91%  |                                                                              |================================================================      |  92%  |                                                                              |=================================================================     |  93%  |                                                                              |==================================================================    |  94%  |                                                                              |==================================================================    |  95%  |                                                                              |===================================================================   |  96%  |                                                                              |====================================================================  |  97%  |                                                                              |===================================================================== |  98%  |                                                                              |===================================================================== |  99%  |                                                                              |======================================================================| 100%
```

```
plot(bis.ncp$criterion~names(bis.ncp$criterion),xlab="number of dimensions", 
     ylab = "ncp criterion")
```

```
ncp <- as.numeric(bis.ncp$ncp[1]) #The optimal number of dimensions(components) which should considered when imputing data, as obtained by k-fold cross validation

bis.impute <- imputePCA(bisScale, ncp = ncp)
bis.impute <- bis.impute$completeObs
bis.impute <- as.data.frame(bis.impute)

bis.impute #Missing values are now imputed according to the PCA cross-validation.
```

```
##            basFro      ectEct      proNas     nasNuc     basTip      ectPro
## 21904  -1.0244926 -0.69001780 -1.44240992 -0.8867058 -1.1337895 -1.58704649
## 16211   1.0025284  0.83957536  1.14266436  0.2835636  0.9812344  1.31520385
## 21903  -0.3236530  0.17234970 -0.29553801  0.2374177 -1.1372544 -0.38638653
## 141977 -0.9114627 -0.82899163 -0.66462612  0.3989657 -0.1461334  0.28173445
## 141976 -1.3293397 -0.98576051 -1.16038298 -0.9663962 -1.3554094 -1.65585751
## 92698   1.0940550  1.07617062 -0.10368424  1.6942182  0.4871016  0.38476341
## 2504   -1.3470355 -1.08062811 -0.61185726 -0.9864332 -0.9281992 -0.88756186
## 2499   -0.3146608 -0.88760045 -0.03562436 -0.5548207 -0.7899435  0.28021832
## 2500   -0.9859829 -1.12762361 -0.85493328 -0.5763015 -0.5285155 -1.06813837
## 2501   -1.2796365 -0.90956611  0.32397557 -1.2807821 -0.8571605 -0.14130941
## 2495    0.2165828 -0.39899885  0.28623876 -0.3632731  0.2982997  0.36085525
## 140290  1.6991021  1.76010978  1.08067101  1.5847851  1.9229912  1.47287169
## 2414    1.4172145 -0.05696192  1.82237914 -0.2964468  0.4527752  1.40018974
## 2494   -0.6050769 -0.52434812  0.64446543 -0.7669970 -0.4303873  0.65492305
## 21905   0.3511776 -0.31921873 -0.11500744  0.2286398 -0.2535899 -0.05381502
## 75033   0.9212758  1.73334294  2.20924974  0.6410545  1.1367856  2.02521395
## 117340  1.0494800  1.25492743 -1.09520715  1.7538807  1.1952431 -0.21516924
## 21906   0.9876352  1.59408072  1.00498388  1.2034788  1.1990291  1.11677084
## 48115   0.3790597  0.68820151  0.26826112 -0.8683517  0.2458679  0.45293213
## 73940  -0.7964860 -0.57731255 -1.13761128 -0.4113915 -0.9582520 -1.13959659
## 2498   -0.8507528 -0.99681935  0.16933444 -1.2865212 -0.9795314 -0.22033498
## 2497   -0.5267634 -0.68120270 -0.45835463 -0.4472142 -1.4724708 -0.53282558
## 2022    1.1772317  0.94629237 -0.79405416  1.6656312 -0.3040322 -0.28932262
##            basWid
## 21904  -0.9101133
## 16211   0.8257795
## 21903  -0.4552358
## 141977 -0.3968481
## 141976 -0.9347375
## 92698   1.6125854
## 2504   -0.8103300
## 2499   -0.6480345
## 2500   -0.6708351
## 2501   -0.6433975
## 2495   -0.4266167
## 140290  1.1841408
## 2414    0.3597209
## 2494   -0.8070995
## 21905  -0.7664437
## 75033   1.7679547
## 117340  1.2035145
## 21906   1.5747547
## 48115   1.2137883
## 73940  -0.6850602
## 2498   -1.0933844
## 2497   -1.0966916
## 2022    0.6025894
```

Hierarchical Clustering

```
bis.impute$sex <- adultSex

bis.impute$sex <- as.factor(bis.impute$sex)

bisNum <- bis.impute[,1:7]

bisScale <- as.data.frame(bisNum)

row_colors <- ifelse(adultSex == "F", "red", 
                     ifelse(adultSex == "M", "blue", "black"))
Heatmap(bisScale,
        name = "Z-score",
        cluster_rows = TRUE,
        cluster_columns = TRUE,
        column_names_rot = 65,
        column_names_gp = gpar(fontsize = 10),
        row_names_gp = gpar(col = row_colors, fontsize = 15),
        row_dend_width = unit(100, "points"))  # <- color the labels
```

```
## Warning: The input is a data frame-like object, convert it to a matrix.
```

```
###ALTERNATIVE CLUSTERING WITH BOOTSTRAP SUPPORT VALUES

bootMap <- pvclust(t(bisScale), method.dist="euclidean", method.hclust="complete", nboot=1000)
```

```
## Bootstrap (r = 0.43)... Done.
## Bootstrap (r = 0.57)... Done.
## Bootstrap (r = 0.71)... Done.
## Bootstrap (r = 0.86)... Done.
## Bootstrap (r = 1.0)... Done.
## Bootstrap (r = 1.14)... Done.
## Bootstrap (r = 1.29)... Done.
```

```
par(mar=c(1.25,2,2,2))
plot(bootMap,labels = FALSE, hang = -1, main = "Hierarchical Clustering of Bison Skulls", print.num = F)
tipLabels <- bootMap$hclust$labels[bootMap$hclust$order]
bisFem <- rownames(bisonSex)[bisonSex$sex=="F"]
bisMale <- rownames(bisonSex)[bisonSex$sex=="M"]
bisNB <- rownames(bisonSex)[bisonSex$sex=="?"]
tipColors <- ifelse(bootMap$hclust$labels[bootMap$hclust$order] %in% bisFem, "red",
                    ifelse(bootMap$hclust$labels[bootMap$hclust$order] %in% bisMale, "blue", "black"))
text(x = 1:length(tipLabels), y = -0.02, labels = tipLabels, col = tipColors,
  srt = 90,adj = 1,xpd = TRUE,cex = 0.75)
```

PCA

```
normPCA <- PCA(bisScale[,1:7])
```

```
#write.csv(normPCA$eig, "newBisonEigVals.csv")
#write.csv(normPCA$var$coord, "neBisonPCLoad.csv")
# write.csv(normPCA$var$cos2, "bisonPCContVar.csv")
# write.csv(normPCA$var$contrib, "bisonVarContPc.csv")
# 
fviz_pca_ind(normPCA)
```

```
fviz_pca_biplot(normPCA)
```

```
coords <- normPCA$ind$coord
coords <- as.data.frame(coords)
bisonSex <- bisonAdults
bisonSex$sex <- adultSex
isFem <- bisonSex$sex=="F"
coords$Fem <- isFem
coords$sex <- adultSex
coords$sex <- as.factor(coords$sex)

hull_sex <- coords %>%
  group_by(sex) %>%
  slice(chull(Dim.1, Dim.2))

coords$Sex <- as.factor(coords$sex)

MyPlot <- ggplot(coords, aes(Dim.1, Dim.2)) + geom_point(shape = 21)
MyPlot + aes(fill = factor(sex)) + geom_polygon(data = hull_sex, alpha = 0.5) +
  labs(x = "PC1", y = "PC2", title = "PCA of Bison Skulls")+ geom_vline(xintercept = 0) + geom_hline(yintercept = 0) +
  theme(legend.title = element_blank()) +
  geom_label_repel(aes(label = rownames(coords))) +
  scale_fill_manual(values = c("#B8B8B8", "#F8766D", "#619CFF"))
```

```
# write.csv(normPCA$eig, "eigVals.csv")
# write.csv(normPCA$var$coord, "pcLoad.csv")
# write.csv(normPCA$var$cos2, "pcContVar.csv")
# write.csv(normPCA$var$contrib, "varContPc.csv")
```

Determine number of clusters data appears to form

```
gapStat <- fviz_nbclust(bisScale[,1:7], kmeans, k.max = 22, method = "gap_stat")
wss <- fviz_nbclust(bisScale[,1:7], kmeans, k.max = 22, method = "wss")
silh <- fviz_nbclust(bisScale[,1:7], kmeans, k.max = 22, method = "silhouette")

wss / silh / gapStat
```

Use k-means clustering to form two most favorable clusters

```
kmBison <- kmeans(bisScale[,1:7], centers = 2)
bisClust <- bisScale
clustVec <- kmBison$cluster
bisClust <- as.data.frame(bisClust)
bisClust$cluster = clustVec
bisClust$cluster <- as.factor(bisClust$cluster)

ClusteredPCA <- PCA(bisClust[,1:7])
```

```
clusterCoords <-ClusteredPCA$ind$coord
clusterCoords <- as.data.frame(coords)
clusterCoords <- clusterCoords[,-c(6,7)]
clusterCoords$cluster <- bisClust$cluster
clusterCoords$Cluster <- as.factor(clusterCoords$cluster)

hull_cluster <- clusterCoords %>%
  group_by(cluster) %>%
  slice(chull(Dim.1, Dim.2))

MyClusterPlot <- ggplot(clusterCoords, aes(x = Dim.1, y = Dim.2)) + geom_point(shape = 21)

MyClusterPlot + aes(fill = cluster) + geom_polygon(data = hull_cluster, alpha = 0.5) +
  labs(x = "PC1", y = "PC2", colour = "Cluster", title = "PCA of Bison Skulls") + 
  geom_vline(xintercept = 0) + geom_hline(yintercept = 0) +
  geom_label_repel(aes(label = rownames(clusterCoords))) +
  scale_fill_manual(values = c("#619CFF", "#F8766D"))
```

```
## Ignoring unknown labels:
## • colour : "Cluster"
```

```
hull_sex$Sex <- as.factor(hull_sex$sex)
hull_cluster$Cluster <- as.factor(hull_cluster$cluster)
```

Plot purported sex and cluster on the same PCA

```
MyClusterPlot +
  geom_polygon(
    data = hull_cluster,
    aes(x = Dim.1, y = Dim.2, group = Cluster, fill = factor(Cluster)),
    alpha = 0.25,
    linewidth = 0.3,
    colour = "gray25",
    inherit.aes = FALSE) +
  scale_fill_manual(name = "Cluster", values = c("1" = "#4DB6AC", "2" = "#D4A72C")) +
  new_scale_fill() +
  geom_point(aes(fill = Sex), shape = 21, size = 3) +
  geom_label_repel(aes(label = rownames(clusterCoords), fill = Sex),colour = "black" ) +
  scale_fill_manual(values = c("?" = "#B8B8B8","F" = "#F8766D","M" = "#619CFF")) +
  labs(x = "PC1 (75.50%)", y = "PC2 (16.47%)", title = "PCA of Bison Skulls") +
  geom_vline(xintercept = 0) +
  geom_hline(yintercept = 0)
```

Use PCA and PERMANOVA to determine if purported females and males
consistently differ across metrics

```
### FIRST NEED TO IMPUTE MISSING DATA

bisonSexScaled <- scale(bisonSub[,1:7])
bisonSexedImputed <- imputePCA(bisonSexScaled)
bisonSexedImputed <- bisonSexedImputed$completeObs
bisonSexedImputed <- as.data.frame(bisonSexedImputed)
bisonSexedImputed$sex <- bisonSub$sex

### PCA

sexedPCA <- PCA(bisonSexedImputed[,1:7], scale.unit=F)
```

```
###########################RUNNING PERMANOVA TO SEE IF PC SCORE IS AFFECTED BY SEX

sexedPCA$eig
```

```
##        eigenvalue percentage of variance cumulative percentage of variance
## comp 1 4.16335259             76.0000747                          76.00007
## comp 2 0.75735470             13.8251596                          89.82523
## comp 3 0.35382896              6.4589840                          96.28422
## comp 4 0.15496388              2.8287939                          99.11301
## comp 5 0.03458645              0.6313597                          99.74437
```

```
sigCols <- sexedPCA$ind$coord[,1:3]
sigCols <- as.data.frame(sigCols)
sigCols$sex <- NA
sigCols$sex <- ifelse(rownames(sigCols) %in% bisFem, "F",
                      ifelse(rownames(sigCols) %in% bisMale, "M", "?"))

sigCols$sex <- as.factor(sigCols$sex)
adonis2(sigCols[,c("Dim.1", "Dim.2", "Dim.3")] ~ sex, data = sigCols, method = "euc")
```

```
## Permutation test for adonis under reduced model
## Permutation: free
## Number of permutations: 999
## 
## adonis2(formula = sigCols[, c("Dim.1", "Dim.2", "Dim.3")] ~ sex, data = sigCols, method = "euc")
##          Df SumOfSqs      R2      F Pr(>F)  
## Model     1   25.715 0.60941 9.3615  0.039 *
## Residual  6   16.481 0.39059                
## Total     7   42.196 1.00000                
## ---
## Signif. codes:  0 '***' 0.001 '**' 0.01 '*' 0.05 '.' 0.1 ' ' 1
```

Lastly, estimate the degree of dimorphism in each metric using finite
mixture analysis and the method of means

```
estimateTable <- matrix(nrow = 7, ncol = 2)
rownames(estimateTable) <- colnames(bisonAdults)
colnames(estimateTable) <- c("FMA", "MM")
estimateTable <- as.data.frame(estimateTable)

pearsonTable <- read.csv("pearsonTable.csv")

for (i in 1:nrow(estimateTable)) {
  metric <- na.omit(bisonAdults[,i])
  mean <- mean(metric)
  OR <- max(metric) - min(metric)
  n <- length(metric)
  k <- pearsonTable$stDevsInRange[pearsonTable$SampleSize == n]
  sigmaTot <- OR/k
  expSub <- k*(2^.5) #expected number of subpop st devs in total OR
  twoSubPercent <- 2/expSub #portion of OR expected to be occupied by 2 sigmaSub
  oneSubPercent <- twoSubPercent/2 #portion of OR expected to be occupied by 1 sigmaSub
  distanceToSub <- oneSubPercent*OR #numeric value of one sigmaSub <- distance from overall mean to mean of each subpop
  bigMean <- mean + distanceToSub
  smallMean <- mean - distanceToSub
  fmaEstimate <- bigMean/smallMean
  estimateTable$FMA[i] <- fmaEstimate
  bigs <- metric[metric > mean(metric)]
  bigMean <- mean(bigs)
  smalls <- metric[metric < mean(metric)]
  smallMean <- mean(smalls)
  mmEstimate <- bigMean/smallMean
  estimateTable$MM[i] <- mmEstimate
}
```
