# Supplementary material for: Linear morphometrics fail to support strong sexual dimorphism in Uintatherium anceps
Source: PLoS One. 2026 Oct 7;21(10):e0345236. doi: 10.1371/journal.pone.0345236 (PMC13645082; doi:10.1371/journal.pone.0345236)
Supplement: Supplementary file 11 [file pone.0345236.s025.html]

Sensitivity and Correlation Analyses


# Sensitivity and Correlation Analyses

#### 2026-07-24

Set wd and load packages

```
#setwd("")

library(ggplot2)
library(dplyr)
```

```
## 
## Attaching package: 'dplyr'
```

```
## The following objects are masked from 'package:stats':
## 
##     filter, lag
```

```
## The following objects are masked from 'package:base':
## 
##     intersect, setdiff, setequal, union
```

```
library(cowplot)
library(gridExtra)
```

```
## 
## Attaching package: 'gridExtra'
```

```
## The following object is masked from 'package:dplyr':
## 
##     combine
```

Load and manipulate uintathere data

```
byHand <- read.csv("Uintathere_By_Hand_Meausurements.csv")
imageJ <- read.csv("Uintathere_ImageJ_Measurements.csv", check.names = F) #load dataframe

#trim and manage dataframes to be directly comparable

imageJ <- imageJ[imageJ$Specimen != "YPM VP 011039L",] #remove damaged view of YPM 11039
rownames(imageJ) <- imageJ[,1] #rename rows as specimen numbers
imageJ <- imageJ[,4:12]
rownames(imageJ) <- gsub("[ -]", ".", rownames(imageJ))
rownames(imageJ)[26] <- "YPM.VPPU.010298"
rownames(imageJ)[12] <- "YPM.VPPU.010076"

byHand <- byHand[,-1]
byHandTrans <- t(byHand)
colnames(byHandTrans) <- byHandTrans[1,]
byHandTrans <- byHandTrans[-1,]
byHandTrans <- as.data.frame(byHandTrans)
byHandShort <- byHandTrans[,c(1,3,2,4,18,28,19,24,22)]

imageJshort <- imageJ[rownames(imageJ) %in% rownames(byHandShort),]
byHandShort <- byHandShort[rownames(byHandShort) %in% rownames(imageJshort),]
byHandSorted <- byHandShort[rownames(imageJshort), ]


byHandClean <- byHandSorted
rownames(byHandClean) <- rownames(imageJshort)

byHandClean[4,1] <- NA
byHandClean[12,1] <- NA
byHandClean[15,1] <- 62
byHandClean[1,2] <- 77
byHandClean[2,2] <- NA
byHandClean[3,2] <- 71
byHandClean[4,2] <- NA
byHandClean[6,2] <- NA
byHandClean[10,2] <- NA
byHandClean[11,2] <- 68
byHandClean[12,2] <- NA
byHandClean[13,2] <- NA
byHandClean[14,2] <- 68
byHandClean[15,2] <- 68
byHandClean[2,3] <- NA
byHandClean[4,3] <- NA
byHandClean[6,3] <- NA
byHandClean[7,3] <- NA
byHandClean[8,3] <- NA
byHandClean[10,3] <- NA
byHandClean[11,3] <- 64
byHandClean[12,3] <- NA
byHandClean[13,3] <- NA
byHandClean[14,3] <- 62.5
byHandClean[15,3] <- 57.5
byHandClean[2,4] <- NA
byHandClean[4,4] <- NA
byHandClean[6,4] <- NA
byHandClean[10,4] <- NA
byHandClean[12,4] <- NA
byHandClean[13,4] <- NA
byHandClean[14,4] <- 29
byHandClean[2,5] <- NA
byHandClean[4,5] <- NA
byHandClean[6,5] <- NA
byHandClean[7,5] <- NA
byHandClean[8,5] <- 40.5
byHandClean[10,5] <- NA
byHandClean[12,5] <- NA
byHandClean[13,5] <- NA
byHandClean[6,6] <- NA
byHandClean[7,6] <- 19
byHandClean[8,6] <- 17
byHandClean[13,6] <- NA
byHandClean[2,7] <- NA
byHandClean[4,7] <- 45
byHandClean[5,7] <- NA
byHandClean[6,7] <- NA
byHandClean[7,7] <- NA
byHandClean[8,7] <- NA
byHandClean[9,7] <- NA
byHandClean[10,7] <- NA
byHandClean[13,7] <- NA
byHandClean[15,7] <- NA
byHandClean[2,8] <- NA
byHandClean[4,8] <- 17.5
byHandClean[5,8] <- NA
byHandClean[6,8] <- NA
byHandClean[7,8] <- NA
byHandClean[8,8] <- NA
byHandClean[9,8] <- NA
byHandClean[10,8] <- NA
byHandClean[13,8] <- NA
byHandClean[15,8] <- NA
byHandClean[6,9] <- NA
byHandClean[7,9] <- NA
byHandClean[8,9] <- NA
byHandClean[10,9] <- NA
byHandClean[13,9] <- NA


metNames <- colnames(imageJshort)
```

Generate Bland-Altman Plots and linear models for each metric

```
plots <- list()

#generate Bland-Altman and Error plots

for (i in 1:length(metNames)) {
  df <- data.frame(
    specimen = rownames(byHandClean),
    hand = byHandClean[,i],
    imageJ = imageJshort[,i]
  )
  
  df$hand <- as.numeric(df$hand)
  df$imageJ <- as.numeric(df$imageJ)
  df <- na.omit(df)
  
  df <- df %>%
    mutate(
      difference = imageJ - hand,
      average = (hand+imageJ) / 2,
      percentBias = difference / average*100
    )
  
  meanBias <- mean(df$percentBias)
  meanDiff <- mean(df$difference)
  correlation <- cor(df$hand, df$imageJ)
  
  cat("Mean percent bias: ", paste(metNames[i]), round(meanBias, 2), "%\n")
  cat("Mean difference: ", paste(metNames[i]), round(meanDiff, 2), "mm\n")
  cat("Correlation: ", paste(metNames[i]), round(correlation, 4), "\n")
  
  diffPlot <- ggplot(df, aes(x = imageJ, y = hand)) +
    geom_point(size = 3) +
    geom_abline(slope = 1, intercept = 0, color = "red", linetype = "dashed") +
    theme_minimal() +
    labs(
      title = paste("Hand vs. ImageJ Measurements", metNames[i]),
      x = "ImageJ Measurement (cm)",
      y = "Hand Measurement (cm)"
    )
  
  BAPlot <- ggplot(df, aes(x = average, y = difference)) +
    geom_point(size = 3) +
    geom_hline(yintercept = meanDiff, color = "red", linetype = "dashed") +
    geom_hline(yintercept = meanDiff + 1.96 * sd(df$difference), linetype = "dotted") +
    geom_hline(yintercept = meanDiff - 1.96 * sd(df$difference), linetype = "dotted") +
    theme_minimal() +
    labs(
      title = paste(metNames[i], "Bland–Altman Plot"),
      x = "Average of Hand and ImageJ (cm)",
      y = "Difference (Hand - ImageJ) (cm)"
    )
  
  lm_model <- lm(diff ~ avg, data = data.frame(diff = df$hand - df$imageJ, 
                                               avg = (df$hand + df$imageJ)/2))
  
  correction_factor <- 1 + (meanBias / 100)
  df <- df %>%
    mutate(correctedImageJ = imageJ * correction_factor)
  
  assign(paste(metNames[i]), df)
  assign(paste(metNames[i], "diffPlot"), diffPlot)
  assign(paste(metNames[i], "BAPlot"), BAPlot)
  assign(paste(metNames[i], "linear model"), lm_model)
}
```

```
## Mean percent bias:  nasOcc -9.58 %
## Mean difference:  nasOcc -6.86 mm
## Correlation:  nasOcc 0.7844 
## Mean percent bias:  nasCon -13.5 %
## Mean difference:  nasCon -8.95 mm
## Correlation:  nasCon 0.8447 
## Mean percent bias:  preCon -10.07 %
## Mean difference:  preCon -6.23 mm
## Correlation:  preCon 0.8941 
## Mean percent bias:  occCon -14.45 %
## Mean difference:  occCon -3.71 mm
## Correlation:  occCon 0.6915 
## Mean percent bias:  parPro 6.77 %
## Mean difference:  parPro 2.5 mm
## Correlation:  parPro 0.9809 
## Mean percent bias:  maxAlv 5.33 %
## Mean difference:  maxAlv 1.17 mm
## Correlation:  maxAlv 0.9431 
## Mean percent bias:  maxCan 6.99 %
## Mean difference:  maxCan 3.16 mm
## Correlation:  maxCan 0.9236 
## Mean percent bias:  alvCan 7.39 %
## Mean difference:  alvCan 1.55 mm
## Correlation:  alvCan 0.6274 
## Mean percent bias:  diaLen 0.67 %
## Mean difference:  diaLen 0.01 mm
## Correlation:  diaLen 0.1835
```

Generate one large dataframe which contains all relevant values

```
colnames(byHandClean) <- metNames
bigDF <- cbind(byHandClean, imageJshort)

for (i in 1:length(metNames)) {
  avg <- paste(metNames[i], "avg")
  bigDF[,avg] <- NA
}

for (i in 1:length(metNames)) {
  dif <- paste(metNames[i], "dif")
  bigDF[dif] <- NA
}

for (i in 1:ncol(bigDF)) {
  bigDF[,i] <- as.numeric(bigDF[,i])
}

for (j in 1:nrow(bigDF)) {
  for (i in 1:9) {
    if (is.na(bigDF[j,i]) == F && is.na(bigDF[j,i+9]) == F){
      bigDF[j,i+18] <- mean(c(bigDF[j,i], bigDF[j,i+9]))
    }
    if (is.na(bigDF[j,i]) == F && is.na(bigDF[j,i+9]) == F){
      bigDF[j,i+27] <- bigDF[j,i] - bigDF[j,i+9]
    }
  }
}

plot(bigDF$`diaLen avg`, bigDF$`diaLen dif`)
```

Run linear models to see if the absolute and relative difference
between by-hand and ImageJ values for a measurement are correlated with
the mean value for that metric

```
summary(lm(nasOcc$difference ~ nasOcc$average))
```

```
## 
## Call:
## lm(formula = nasOcc$difference ~ nasOcc$average)
## 
## Residuals:
##     Min      1Q  Median      3Q     Max 
## -7.3146 -2.8425  0.7719  2.6992  4.8311 
## 
## Coefficients:
##                Estimate Std. Error t value Pr(>|t|)
## (Intercept)    -19.6177    15.1033  -1.299    0.221
## nasOcc$average   0.1763     0.2081   0.847    0.415
## 
## Residual standard error: 3.595 on 11 degrees of freedom
## Multiple R-squared:  0.06119,    Adjusted R-squared:  -0.02415 
## F-statistic: 0.717 on 1 and 11 DF,  p-value: 0.4152
```

```
summary(lm(nasOcc$percentBias ~ nasOcc$average))
```

```
## 
## Call:
## lm(formula = nasOcc$percentBias ~ nasOcc$average)
## 
## Residuals:
##     Min      1Q  Median      3Q     Max 
## -10.991  -3.626   1.164   3.326   6.874 
## 
## Coefficients:
##                Estimate Std. Error t value Pr(>|t|)
## (Intercept)    -34.7977    21.5875  -1.612    0.135
## nasOcc$average   0.3483     0.2975   1.171    0.266
## 
## Residual standard error: 5.138 on 11 degrees of freedom
## Multiple R-squared:  0.1108, Adjusted R-squared:  0.02995 
## F-statistic: 1.371 on 1 and 11 DF,  p-value: 0.2665
```

```
summary(lm(nasCon$difference ~ nasCon$average))
```

```
## 
## Call:
## lm(formula = nasCon$difference ~ nasCon$average)
## 
## Residuals:
##     Min      1Q  Median      3Q     Max 
## -3.7737 -1.7545  0.8849  1.6248  2.2809 
## 
## Coefficients:
##                Estimate Std. Error t value Pr(>|t|)  
## (Intercept)    -38.0203    15.2562  -2.492    0.047 *
## nasCon$average   0.4325     0.2266   1.908    0.105  
## ---
## Signif. codes:  0 '***' 0.001 '**' 0.01 '*' 0.05 '.' 0.1 ' ' 1
## 
## Residual standard error: 2.453 on 6 degrees of freedom
## Multiple R-squared:  0.3777, Adjusted R-squared:  0.274 
## F-statistic: 3.642 on 1 and 6 DF,  p-value: 0.1049
```

```
summary(lm(nasCon$percentBias ~ nasCon$average))
```

```
## 
## Call:
## lm(formula = nasCon$percentBias ~ nasCon$average)
## 
## Residuals:
##    Min     1Q Median     3Q    Max 
## -6.003 -2.426  1.384  2.392  3.616 
## 
## Coefficients:
##                Estimate Std. Error t value Pr(>|t|)  
## (Intercept)    -69.7499    23.1059  -3.019   0.0234 *
## nasCon$average   0.8369     0.3432   2.438   0.0506 .
## ---
## Signif. codes:  0 '***' 0.001 '**' 0.01 '*' 0.05 '.' 0.1 ' ' 1
## 
## Residual standard error: 3.714 on 6 degrees of freedom
## Multiple R-squared:  0.4977, Adjusted R-squared:  0.414 
## F-statistic: 5.946 on 1 and 6 DF,  p-value: 0.05058
```

```
summary(lm(preCon$difference ~ preCon$average))
```

```
## 
## Call:
## lm(formula = preCon$difference ~ preCon$average)
## 
## Residuals:
##       1       2       3       4       5       6 
## -1.2989 -0.7814 -0.7190  3.0753 -3.1643  2.8882 
## 
## Coefficients:
##                Estimate Std. Error t value Pr(>|t|)
## (Intercept)    -15.5085    14.7954  -1.048    0.354
## preCon$average   0.1479     0.2352   0.629    0.564
## 
## Residual standard error: 2.767 on 4 degrees of freedom
## Multiple R-squared:  0.08998,    Adjusted R-squared:  -0.1375 
## F-statistic: 0.3955 on 1 and 4 DF,  p-value: 0.5635
```

```
summary(lm(preCon$percentBias ~ preCon$average))
```

```
## 
## Call:
## lm(formula = preCon$percentBias ~ preCon$average)
## 
## Residuals:
##      1      2      3      4      5      6 
## -1.806 -1.077 -1.079  4.598 -5.411  4.775 
## 
## Coefficients:
##                Estimate Std. Error t value Pr(>|t|)
## (Intercept)    -33.1860    23.7318  -1.398    0.235
## preCon$average   0.3686     0.3773   0.977    0.384
## 
## Residual standard error: 4.438 on 4 degrees of freedom
## Multiple R-squared:  0.1926, Adjusted R-squared:  -0.009216 
## F-statistic: 0.9543 on 1 and 4 DF,  p-value: 0.3839
```

```
summary(lm(occCon$difference ~ occCon$average))
```

```
## 
## Call:
## lm(formula = occCon$difference ~ occCon$average)
## 
## Residuals:
##     Min      1Q  Median      3Q     Max 
## -3.0118 -1.0575 -0.1559  1.1332  3.0341 
## 
## Coefficients:
##                Estimate Std. Error t value Pr(>|t|)
## (Intercept)     -7.9421     9.0523  -0.877    0.414
## occCon$average   0.1627     0.3468   0.469    0.655
## 
## Residual standard error: 2.052 on 6 degrees of freedom
## Multiple R-squared:  0.0354, Adjusted R-squared:  -0.1254 
## F-statistic: 0.2202 on 1 and 6 DF,  p-value: 0.6555
```

```
summary(lm(occCon$percentBias ~ occCon$average))
```

```
## 
## Call:
## lm(formula = occCon$percentBias ~ occCon$average)
## 
## Residuals:
##      Min       1Q   Median       3Q      Max 
## -11.8637  -3.9863  -0.4684   4.3717  11.5314 
## 
## Coefficients:
##                Estimate Std. Error t value Pr(>|t|)
## (Intercept)     -44.962     34.789  -1.292    0.244
## occCon$average    1.173      1.333   0.880    0.413
## 
## Residual standard error: 7.887 on 6 degrees of freedom
## Multiple R-squared:  0.1143, Adjusted R-squared:  -0.03332 
## F-statistic: 0.7743 on 1 and 6 DF,  p-value: 0.4127
```

```
summary(lm(parPro$difference ~ parPro$average))
```

```
## 
## Call:
## lm(formula = parPro$difference ~ parPro$average)
## 
## Residuals:
##     Min      1Q  Median      3Q     Max 
## -1.9824 -0.6926  0.1713  0.6922  1.9647 
## 
## Coefficients:
##                Estimate Std. Error t value Pr(>|t|)
## (Intercept)    -0.12258    3.00023  -0.041    0.969
## parPro$average  0.07094    0.08001   0.887    0.409
## 
## Residual standard error: 1.375 on 6 degrees of freedom
## Multiple R-squared:  0.1158, Adjusted R-squared:  -0.03153 
## F-statistic: 0.7861 on 1 and 6 DF,  p-value: 0.4094
```

```
summary(lm(parPro$percentBias ~ parPro$average))
```

```
## 
## Call:
## lm(formula = parPro$percentBias ~ parPro$average)
## 
## Residuals:
##     Min      1Q  Median      3Q     Max 
## -5.3226 -1.7076  0.3863  1.8535  4.6612 
## 
## Coefficients:
##                Estimate Std. Error t value Pr(>|t|)
## (Intercept)     7.07284    7.59270   0.932    0.388
## parPro$average -0.00816    0.20249  -0.040    0.969
## 
## Residual standard error: 3.479 on 6 degrees of freedom
## Multiple R-squared:  0.0002706,  Adjusted R-squared:  -0.1664 
## F-statistic: 0.001624 on 1 and 6 DF,  p-value: 0.9692
```

```
summary(lm(maxAlv$difference ~ maxAlv$average))
```

```
## 
## Call:
## lm(formula = maxAlv$difference ~ maxAlv$average)
## 
## Residuals:
##     Min      1Q  Median      3Q     Max 
## -3.1799 -0.8079  0.4685  1.1031  1.7186 
## 
## Coefficients:
##                Estimate Std. Error t value Pr(>|t|)
## (Intercept)     1.48756    2.79930   0.531    0.610
## maxAlv$average -0.01398    0.12103  -0.116    0.911
## 
## Residual standard error: 1.629 on 8 degrees of freedom
## Multiple R-squared:  0.001666,   Adjusted R-squared:  -0.1231 
## F-statistic: 0.01335 on 1 and 8 DF,  p-value: 0.9109
```

```
summary(lm(maxAlv$percentBias ~ maxAlv$average))
```

```
## 
## Call:
## lm(formula = maxAlv$percentBias ~ maxAlv$average)
## 
## Residuals:
##     Min      1Q  Median      3Q     Max 
## -11.124  -3.753   2.477   4.352   6.008 
## 
## Coefficients:
##                Estimate Std. Error t value Pr(>|t|)
## (Intercept)     10.6334    10.8159   0.983    0.354
## maxAlv$average  -0.2332     0.4676  -0.499    0.631
## 
## Residual standard error: 6.294 on 8 degrees of freedom
## Multiple R-squared:  0.03016,    Adjusted R-squared:  -0.09107 
## F-statistic: 0.2488 on 1 and 8 DF,  p-value: 0.6314
```

```
summary(lm(alvCan$difference ~ alvCan$average))
```

```
## 
## Call:
## lm(formula = alvCan$difference ~ alvCan$average)
## 
## Residuals:
##       1       2       3       4       5       6 
## -0.2961 -1.0562  4.1060 -1.9163 -1.3343  0.4969 
## 
## Coefficients:
##                Estimate Std. Error t value Pr(>|t|)
## (Intercept)     -8.0271     8.9535  -0.897    0.421
## alvCan$average   0.4898     0.4549   1.077    0.342
## 
## Residual standard error: 2.437 on 4 degrees of freedom
## Multiple R-squared:  0.2247, Adjusted R-squared:  0.0309 
## F-statistic: 1.159 on 1 and 4 DF,  p-value: 0.3422
```

```
summary(lm(alvCan$percentBias ~ alvCan$average))
```

```
## 
## Call:
## lm(formula = alvCan$percentBias ~ alvCan$average)
## 
## Residuals:
##      1      2      3      4      5      6 
## -1.574 -5.115 20.232 -9.267 -7.135  2.859 
## 
## Coefficients:
##                Estimate Std. Error t value Pr(>|t|)
## (Intercept)     -36.850     44.347  -0.831    0.453
## alvCan$average    2.262      2.253   1.004    0.372
## 
## Residual standard error: 12.07 on 4 degrees of freedom
## Multiple R-squared:  0.2012, Adjusted R-squared:  0.001537 
## F-statistic: 1.008 on 1 and 4 DF,  p-value: 0.3723
```

```
summary(lm(diaLen$difference ~ diaLen$average))
```

```
## 
## Call:
## lm(formula = diaLen$difference ~ diaLen$average)
## 
## Residuals:
##      Min       1Q   Median       3Q      Max 
## -2.48809 -0.74721  0.01338  1.14036  1.39769 
## 
## Coefficients:
##                Estimate Std. Error t value Pr(>|t|)
## (Intercept)      3.6237     4.0615   0.892    0.398
## diaLen$average  -0.5056     0.5648  -0.895    0.397
## 
## Residual standard error: 1.318 on 8 degrees of freedom
## Multiple R-squared:  0.09107,    Adjusted R-squared:  -0.02255 
## F-statistic: 0.8015 on 1 and 8 DF,  p-value: 0.3968
```

```
summary(lm(diaLen$percentBias ~ diaLen$average))
```

```
## 
## Call:
## lm(formula = diaLen$percentBias ~ diaLen$average)
## 
## Residuals:
##     Min      1Q  Median      3Q     Max 
## -34.903  -9.708   0.181  14.692  21.516 
## 
## Coefficients:
##                Estimate Std. Error t value Pr(>|t|)
## (Intercept)      55.113     56.788   0.971    0.360
## diaLen$average   -7.610      7.897  -0.964    0.363
## 
## Residual standard error: 18.43 on 8 degrees of freedom
## Multiple R-squared:  0.104,  Adjusted R-squared:  -0.007977 
## F-statistic: 0.9288 on 1 and 8 DF,  p-value: 0.3634
```

Generate plots showing the range in percentage differences for each
metric

```
yMarks <- seq(-40, 40, by = 10)

plot(nasOcc$percentBias ~ nasOcc$average, ylim = c(-40, 40),
     xlab = "Average of Hand and ImageJ (cm)",
     ylab = "% Difference",
     main = "nasOcc Percentage Difference")
axis(side = 2, at = yMarks)
abline(h = yMarks, col = "lightgray", lty = "dotted")
```

```
range(nasOcc$percentBias)
```

```
## [1] -22.286086  -3.267957
```

```
mean(nasOcc$percentBias)
```

```
## [1] -9.580714
```

```
sd(nasOcc$percentBias)
```

```
## [1] 5.216976
```

```
plot(nasCon$percentBias ~ nasCon$average, ylim = c(-40, 40),
     xlab = "Average of Hand and ImageJ (cm)",
     ylab = "% Difference",
     main = "nasCon Percentage Difference")
axis(side = 2, at = yMarks)
abline(h = yMarks, col = "lightgray", lty = "dotted")
```

```
range(nasCon$percentBias)
```

```
## [1] -22.786638  -5.953322
```

```
mean(nasCon$percentBias)
```

```
## [1] -13.50027
```

```
sd(nasCon$percentBias)
```

```
## [1] 4.852385
```

```
plot(preCon$percentBias ~ preCon$average, ylim = c(-40, 40),
     xlab = "Average of Hand and ImageJ (cm)",
     ylab = "% Difference",
     main = "preCon Percentage Difference")
axis(side = 2, at = yMarks)
abline(h = yMarks, col = "lightgray", lty = "dotted")
```

```
range(preCon$percentBias)
```

```
## [1] -16.839986  -3.400809
```

```
mean(preCon$percentBias)
```

```
## [1] -10.07001
```

```
sd(preCon$percentBias)
```

```
## [1] 4.418062
```

```
plot(occCon$percentBias ~ occCon$average, ylim = c(-40, 40),
     xlab = "Average of Hand and ImageJ (cm)",
     ylab = "% Difference",
     main = "occCon Percentage Difference")
axis(side = 2, at = yMarks)
abline(h = yMarks, col = "lightgray", lty = "dotted")
```

```
range(occCon$percentBias)
```

```
## [1] -27.436785  -2.097553
```

```
mean(occCon$percentBias)
```

```
## [1] -14.4486
```

```
sd(occCon$percentBias)
```

```
## [1] 7.758811
```

```
plot(parPro$percentBias ~ parPro$average, ylim = c(-40, 40),
     xlab = "Average of Hand and ImageJ (cm)",
     ylab = "% Difference",
     main = "parPro Percentage Difference")
axis(side = 2, at = yMarks)
abline(h = yMarks, col = "lightgray", lty = "dotted")
```

```
range(parPro$percentBias)
```

```
## [1]  1.446095 11.383556
```

```
mean(parPro$percentBias)
```

```
## [1] 6.7709
```

```
sd(parPro$percentBias)
```

```
## [1] 3.221634
```

```
plot(maxAlv$percentBias ~ maxAlv$average, ylim = c(-40, 40),
     xlab = "Average of Hand and ImageJ (cm)",
     ylab = "% Difference",
     main = "maxAlv Percentage Difference")
axis(side = 2, at = yMarks)
abline(h = yMarks, col = "lightgray", lty = "dotted")
```

```
range(maxAlv$percentBias)
```

```
## [1] -7.243522 11.445752
```

```
mean(maxAlv$percentBias)
```

```
## [1] 5.330734
```

```
sd(maxAlv$percentBias)
```

```
## [1] 6.025754
```

```
plot(alvCan$percentBias ~ alvCan$average, ylim = c(-40, 40),
     xlab = "Average of Hand and ImageJ (cm)",
     ylab = "% Difference",
     main = "alvCan Percentage Difference")
axis(side = 2, at = yMarks)
abline(h = yMarks, col = "lightgray", lty = "dotted")
```

```
range(alvCan$percentBias)
```

```
## [1] -4.089243 29.928793
```

```
mean(alvCan$percentBias)
```

```
## [1] 7.391057
```

```
sd(alvCan$percentBias)
```

```
## [1] 12.08145
```

```
plot(diaLen$percentBias ~ diaLen$average, ylim = c(-40, 40),
     xlab = "Average of Hand and ImageJ (cm)",
     ylab = "% Difference",
     main = "diaLen Percentage Difference")
axis(side = 2, at = yMarks)
abline(h = yMarks, col = "lightgray", lty = "dotted")
```

```
range(diaLen$percentBias)
```

```
## [1] -34.87326  27.96792
```

```
mean(diaLen$percentBias)
```

```
## [1] 0.6737751
```

```
sd(diaLen$percentBias)
```

```
## [1] 18.35743
```

```
grid.arrange(`nasOcc BAPlot`, `nasCon BAPlot`, `preCon BAPlot`, `occCon BAPlot`, `parPro BAPlot`, `maxAlv BAPlot`,
             `alvCan BAPlot`, `diaLen BAPlot`, ncol = 2, nrow = 4)
```

Test to see if canine width is correlated with canine length

```
uintatheres <- read.csv("Uintathere_Measurements.csv")
plot(uintatheres$canWid ~ uintatheres$alvCan)
```

```
canModel <- lm(uintatheres$canWid ~ uintatheres$alvCan)
summary(canModel)
```

```
## 
## Call:
## lm(formula = uintatheres$canWid ~ uintatheres$alvCan)
## 
## Residuals:
##      Min       1Q   Median       3Q      Max 
## -0.98974 -0.38503  0.02552  0.26722  1.17460 
## 
## Coefficients:
##                    Estimate Std. Error t value Pr(>|t|)   
## (Intercept)         3.73338    0.89125   4.189  0.00304 **
## uintatheres$alvCan  0.13230    0.04457   2.969  0.01791 * 
## ---
## Signif. codes:  0 '***' 0.001 '**' 0.01 '*' 0.05 '.' 0.1 ' ' 1
## 
## Residual standard error: 0.6323 on 8 degrees of freedom
##   (17 observations deleted due to missingness)
## Multiple R-squared:  0.5242, Adjusted R-squared:  0.4647 
## F-statistic: 8.812 on 1 and 8 DF,  p-value: 0.01791
```

Working with bison now… First, demonstrate that skull length as
measured in the frontal plane is directly comparable to skull length
measured in the sagittal plane

```
bisonScans <- read.csv("Bison_Morphosource.csv")
bisonScans <- bisonScans[1:5, 1:3]
rownames(bisonScans) <- bisonScans[,1]
bisonScans <- bisonScans[,2:3]
colnames(bisonScans) <- c("Anterior", "Lateral")
bisonScans$Diff <- NA
for (i in 1:nrow(bisonScans)) {
  bisonScans$Diff[i] <- ((bisonScans$Anterior[i]-bisonScans$Lateral[i])/
                            mean(c(bisonScans$Anterior[i], bisonScans$Lateral[i]))) * 100
}
bisonScans$Average <- NA
for (i in 1:nrow(bisonScans)) {
  bisonScans$Average[i] <- mean(c(bisonScans$Anterior[i], bisonScans$Lateral[i]))
}
plot(bisonScans$Diff ~ bisonScans$Average, ylim = c(-40, 40))
```

```
range(bisonScans$Diff)
```

```
## [1] -1.894918  2.158273
```

```
mean(bisonScans$Diff)
```

```
## [1] 0.2972924
```

```
sd(bisonScans$Diff)
```

```
## [1] 1.686314
```

```
model <- lm(bisonScans$Anterior ~ bisonScans$Lateral)
summary(model)
```

```
## 
## Call:
## lm(formula = bisonScans$Anterior ~ bisonScans$Lateral)
## 
## Residuals:
##          1          2          3          4          5 
##  0.0068417  0.0108155  0.0008155 -0.0098475 -0.0086252 
## 
## Coefficients:
##                    Estimate Std. Error t value Pr(>|t|)  
## (Intercept)         0.03689    0.08967   0.411   0.7084  
## bisonScans$Lateral  0.93508    0.16473   5.677   0.0108 *
## ---
## Signif. codes:  0 '***' 0.001 '**' 0.01 '*' 0.05 '.' 0.1 ' ' 1
## 
## Residual standard error: 0.01058 on 3 degrees of freedom
## Multiple R-squared:  0.9148, Adjusted R-squared:  0.8864 
## F-statistic: 32.22 on 1 and 3 DF,  p-value: 0.01083
```

```
dev.off()
```

```
## null device 
##           1
```

```
plot(bisonScans$Anterior ~ bisonScans$Lateral, xlab = "Lateral measurement (m)", ylab = "Anterior measurement (m)", 
     main= "Bison skull length: Lateral vs Anterior")
abline(model, col = "red", lwd = 2)
rect(0.5, 0.567, 0.520, 0.572, col = "lightblue")
text(0.51, 0.57, expression(R^2 == 0.9148))
```

Generate Bland-Altman plots and store relevant statistics for
Bison

```
bisonImageJ <- read.csv("Bison_Measurements.csv")
bisonByHand <- read.csv("Bison_By_Hand_Measurements.csv")
bisonImageJ <- bisonImageJ[-20,]#remove 68350, specimen with adult dentition still erupting
bisonByHand <- bisonByHand[-20,]#remove 68350, specimen with adult dentition still erupting
rownames(bisonImageJ) <- bisonImageJ[,1]
rownames(bisonByHand) <- bisonByHand[,1]
bisonImageJ <- bisonImageJ[,-c(1:4)]
bisonByHand <- bisonByHand[,-1]
bisonByHand <- bisonByHand[,colnames(bisonByHand) %in% colnames(bisonImageJ)]
bisonImageJ <- bisonImageJ[,colnames(bisonImageJ) %in% colnames(bisonByHand)]
bisonByHand <- bisonByHand %>% relocate(basFro, .after = tipFro)

metNames <- colnames(bisonImageJ)

plots <- list()

for (i in 1:length(metNames)) {
  df <- data.frame(
    specimen = rownames(bisonByHand),
    hand = bisonByHand[,i],
    imageJ = bisonImageJ[,i]
  )
  
  df$hand <- as.numeric(df$hand)
  df$imageJ <- as.numeric(df$imageJ)
  df <- na.omit(df)
  
  df <- df %>%
    mutate(
      difference = imageJ - hand,
      average = (hand+imageJ) / 2,
      percentBias = difference / average*100
    )
  
  meanBias <- mean(df$percentBias)
  meanDiff <- mean(df$difference)
  correlation <- cor(df$hand, df$imageJ)
  
  cat("Mean percent bias: ", paste(metNames[i]), round(meanBias, 2), "%\n")
  cat("Mean difference: ", paste(metNames[i]), round(meanDiff, 2), "mm\n")
  cat("Correlation: ", paste(metNames[i]), round(correlation, 4), "\n")
  
  diffPlot <- ggplot(df, aes(x = imageJ, y = hand)) +
    geom_point(size = 3) +
    geom_abline(slope = 1, intercept = 0, color = "red", linetype = "dashed") +
    theme_minimal() +
    labs(
      title = paste("Hand vs. ImageJ Measurements", metNames[i]),
      x = "ImageJ Measurement (cm)",
      y = "Hand Measurement (cm)"
    )
  
  BAPlot <- ggplot(df, aes(x = average, y = difference)) +
    geom_point(size = 3) +
    geom_hline(yintercept = meanDiff, color = "red", linetype = "dashed") +
    geom_hline(yintercept = meanDiff + 1.96 * sd(df$difference), linetype = "dotted") +
    geom_hline(yintercept = meanDiff - 1.96 * sd(df$difference), linetype = "dotted") +
    theme_minimal() +
    labs(
      title = paste(metNames[i], "Bland–Altman Plot"),
      x = "Average of Hand and ImageJ (cm)",
      y = "Difference (Hand - ImageJ) (cm)"
    )
  
  lm_model <- lm(diff ~ avg, data = data.frame(diff = df$hand - df$imageJ, 
                                               avg = (df$hand + df$imageJ)/2))
  
  correction_factor <- 1 + (meanBias / 100)
  df <- df %>%
    mutate(correctedImageJ = imageJ * correction_factor)
  
  assign(paste(metNames[i]), df)
  assign(paste(metNames[i], "diffPlot"), diffPlot)
  assign(paste(metNames[i], "BAPlot"), BAPlot)
  assign(paste(metNames[i], "linear model"), lm_model)
}
```

```
## Mean percent bias:  tipFro -3.83 %
## Mean difference:  tipFro -2 mm
## Correlation:  tipFro 0.9829 
## Mean percent bias:  basFro -11.82 %
## Mean difference:  basFro -2.99 mm
## Correlation:  basFro 0.9503 
## Mean percent bias:  ectEct -8.67 %
## Mean difference:  ectEct -2.47 mm
## Correlation:  ectEct 0.9809 
## Mean percent bias:  maxMax -18.63 %
## Mean difference:  maxMax -2.98 mm
## Correlation:  maxMax 0.8992 
## Mean percent bias:  proNas -13.07 %
## Mean difference:  proNas -3.59 mm
## Correlation:  proNas 0.6746 
## Mean percent bias:  nasNuc -17.62 %
## Mean difference:  nasNuc -3.53 mm
## Correlation:  nasNuc 0.9508 
## Mean percent bias:  basTip 11.41 %
## Mean difference:  basTip 1.73 mm
## Correlation:  basTip 0.9419 
## Mean percent bias:  ectPro -12.34 %
## Mean difference:  ectPro -4.27 mm
## Correlation:  ectPro 0.6966
```

```
for (i in 1:length(metNames)) {
  diffPlot <- paste(metNames[i], "diffPlot")
  baPlot <- paste(metNames[i], "BAPlot")
  print(get(diffPlot))
  print(get(baPlot))
}
```

```
for (i in 1:length(metNames)) {
  plotName <- paste(metNames[i], "BAPlot")
  print(get(plotName))
}
```

```
bigDF <- cbind(bisonByHand, bisonImageJ)

for (i in 1:length(metNames)) {
  avg <- paste(metNames[i], "avg")
  bigDF[,avg] <- NA
}

for (i in 1:length(metNames)) {
  dif <- paste(metNames[i], "dif")
  bigDF[dif] <- NA
}

for (i in 1:ncol(bigDF)) {
  bigDF[,i] <- as.numeric(bigDF[,i])
}

for (j in 1:nrow(bigDF)) {
  for (i in 1:8) {
    if (is.na(bigDF[j,i]) == F && is.na(bigDF[j,i+8]) == F){
      bigDF[j,i+16] <- mean(c(bigDF[j,i], bigDF[j,i+8]))
    }
    if (is.na(bigDF[j,i]) == F && is.na(bigDF[j,i+8]) == F){
      bigDF[j,i+24] <- bigDF[j,i] - bigDF[j,i+8]
    }
  }
}
```

Perform linear models in Bison

```
summary(lm(basFro$difference ~ basFro$average))
```

```
## 
## Call:
## lm(formula = basFro$difference ~ basFro$average)
## 
## Residuals:
##     Min      1Q  Median      3Q     Max 
## -1.3662 -0.8106 -0.2780  0.6556  2.4474 
## 
## Coefficients:
##                Estimate Std. Error t value Pr(>|t|)   
## (Intercept)      3.1702     1.7115   1.852  0.07809 . 
## basFro$average  -0.2494     0.0686  -3.636  0.00155 **
## ---
## Signif. codes:  0 '***' 0.001 '**' 0.01 '*' 0.05 '.' 0.1 ' ' 1
## 
## Residual standard error: 1.198 on 21 degrees of freedom
## Multiple R-squared:  0.3863, Adjusted R-squared:  0.3571 
## F-statistic: 13.22 on 1 and 21 DF,  p-value: 0.001546
```

```
summary(lm(basFro$percentBias ~ basFro$average))
```

```
## 
## Call:
## lm(formula = basFro$percentBias ~ basFro$average)
## 
## Residuals:
##    Min     1Q Median     3Q    Max 
## -6.197 -3.511 -1.016  2.554 12.645 
## 
## Coefficients:
##                Estimate Std. Error t value Pr(>|t|)  
## (Intercept)      1.1680     7.3029   0.160   0.8745  
## basFro$average  -0.5261     0.2927  -1.797   0.0867 .
## ---
## Signif. codes:  0 '***' 0.001 '**' 0.01 '*' 0.05 '.' 0.1 ' ' 1
## 
## Residual standard error: 5.112 on 21 degrees of freedom
## Multiple R-squared:  0.1333, Adjusted R-squared:  0.09203 
## F-statistic:  3.23 on 1 and 21 DF,  p-value: 0.0867
```

```
summary(lm(ectEct$difference ~ ectEct$average))
```

```
## 
## Call:
## lm(formula = ectEct$difference ~ ectEct$average)
## 
## Residuals:
##      Min       1Q   Median       3Q      Max 
## -1.37179 -0.44720  0.03378  0.49930  1.28000 
## 
## Coefficients:
##                Estimate Std. Error t value Pr(>|t|)    
## (Intercept)     2.33485    1.20335    1.94 0.065890 .  
## ectEct$average -0.17119    0.04258   -4.02 0.000619 ***
## ---
## Signif. codes:  0 '***' 0.001 '**' 0.01 '*' 0.05 '.' 0.1 ' ' 1
## 
## Residual standard error: 0.702 on 21 degrees of freedom
## Multiple R-squared:  0.4349, Adjusted R-squared:  0.408 
## F-statistic: 16.16 on 1 and 21 DF,  p-value: 0.0006193
```

```
summary(lm(ectEct$percentBias ~ ectEct$average))
```

```
## 
## Call:
## lm(formula = ectEct$percentBias ~ ectEct$average)
## 
## Residuals:
##     Min      1Q  Median      3Q     Max 
## -5.4203 -1.5624  0.0935  2.0555  3.9232 
## 
## Coefficients:
##                Estimate Std. Error t value Pr(>|t|)  
## (Intercept)     -0.6705     4.2432  -0.158   0.8759  
## ectEct$average  -0.2853     0.1502  -1.900   0.0712 .
## ---
## Signif. codes:  0 '***' 0.001 '**' 0.01 '*' 0.05 '.' 0.1 ' ' 1
## 
## Residual standard error: 2.475 on 21 degrees of freedom
## Multiple R-squared:  0.1467, Adjusted R-squared:  0.1061 
## F-statistic: 3.611 on 1 and 21 DF,  p-value: 0.0712
```

```
summary(lm(proNas$difference ~ proNas$average))
```

```
## 
## Call:
## lm(formula = proNas$difference ~ proNas$average)
## 
## Residuals:
##     Min      1Q  Median      3Q     Max 
## -4.2626 -0.6565  0.3877  0.9035  1.8589 
## 
## Coefficients:
##                Estimate Std. Error t value Pr(>|t|)
## (Intercept)     -1.9097     5.7178  -0.334    0.742
## proNas$average  -0.0610     0.2077  -0.294    0.772
## 
## Residual standard error: 1.586 on 18 degrees of freedom
## Multiple R-squared:  0.004772,   Adjusted R-squared:  -0.05052 
## F-statistic: 0.0863 on 1 and 18 DF,  p-value: 0.7723
```

```
summary(lm(proNas$percentBias ~ proNas$average))
```

```
## 
## Call:
## lm(formula = proNas$percentBias ~ proNas$average)
## 
## Residuals:
##     Min      1Q  Median      3Q     Max 
## -15.433  -2.567   1.387   3.156   6.853 
## 
## Coefficients:
##                Estimate Std. Error t value Pr(>|t|)
## (Intercept)    -18.7567    20.9303  -0.896    0.382
## proNas$average   0.2069     0.7601   0.272    0.789
## 
## Residual standard error: 5.805 on 18 degrees of freedom
## Multiple R-squared:  0.004098,   Adjusted R-squared:  -0.05123 
## F-statistic: 0.07407 on 1 and 18 DF,  p-value: 0.7886
```

```
summary(lm(nasNuc$difference ~ nasNuc$average))
```

```
## 
## Call:
## lm(formula = nasNuc$difference ~ nasNuc$average)
## 
## Residuals:
##     Min      1Q  Median      3Q     Max 
## -1.7389 -0.5097  0.2073  0.4216  1.4769 
## 
## Coefficients:
##                Estimate Std. Error t value Pr(>|t|)
## (Intercept)    -1.50879    1.40408  -1.075    0.295
## nasNuc$average -0.10016    0.06915  -1.448    0.162
## 
## Residual standard error: 0.8347 on 21 degrees of freedom
## Multiple R-squared:  0.09083,    Adjusted R-squared:  0.04754 
## F-statistic: 2.098 on 1 and 21 DF,  p-value: 0.1623
```

```
summary(lm(nasNuc$percentBias ~ nasNuc$average))
```

```
## 
## Call:
## lm(formula = nasNuc$percentBias ~ nasNuc$average)
## 
## Residuals:
##     Min      1Q  Median      3Q     Max 
## -7.6247 -2.6903  0.6867  2.3452  6.1402 
## 
## Coefficients:
##                Estimate Std. Error t value Pr(>|t|)   
## (Intercept)    -24.9659     6.5550  -3.809  0.00103 **
## nasNuc$average   0.3647     0.3228   1.130  0.27136   
## ---
## Signif. codes:  0 '***' 0.001 '**' 0.01 '*' 0.05 '.' 0.1 ' ' 1
## 
## Residual standard error: 3.897 on 21 degrees of freedom
## Multiple R-squared:  0.05729,    Adjusted R-squared:  0.0124 
## F-statistic: 1.276 on 1 and 21 DF,  p-value: 0.2714
```

```
summary(lm(basTip$difference ~ basTip$average))
```

```
## 
## Call:
## lm(formula = basTip$difference ~ basTip$average)
## 
## Residuals:
##     Min      1Q  Median      3Q     Max 
## -2.3424 -0.9120  0.2219  0.8227  2.4041 
## 
## Coefficients:
##                Estimate Std. Error t value Pr(>|t|)  
## (Intercept)     2.51949    1.42577   1.767   0.0963 .
## basTip$average -0.04917    0.08642  -0.569   0.5773  
## ---
## Signif. codes:  0 '***' 0.001 '**' 0.01 '*' 0.05 '.' 0.1 ' ' 1
## 
## Residual standard error: 1.297 on 16 degrees of freedom
## Multiple R-squared:  0.01983,    Adjusted R-squared:  -0.04143 
## F-statistic: 0.3237 on 1 and 16 DF,  p-value: 0.5773
```

```
summary(lm(basTip$percentBias ~ basTip$average))
```

```
## 
## Call:
## lm(formula = basTip$percentBias ~ basTip$average)
## 
## Residuals:
##      Min       1Q   Median       3Q      Max 
## -15.2102  -5.3970   0.9244   4.4451  13.8052 
## 
## Coefficients:
##                Estimate Std. Error t value Pr(>|t|)  
## (Intercept)     25.8511     9.2864   2.784   0.0133 *
## basTip$average  -0.8959     0.5629  -1.592   0.1310  
## ---
## Signif. codes:  0 '***' 0.001 '**' 0.01 '*' 0.05 '.' 0.1 ' ' 1
## 
## Residual standard error: 8.447 on 16 degrees of freedom
## Multiple R-squared:  0.1367, Adjusted R-squared:  0.08273 
## F-statistic: 2.533 on 1 and 16 DF,  p-value: 0.131
```

```
summary(lm(ectPro$difference ~ ectPro$average))
```

```
## 
## Call:
## lm(formula = ectPro$difference ~ ectPro$average)
## 
## Residuals:
##     Min      1Q  Median      3Q     Max 
## -3.1895 -0.6659  0.2048  1.1977  1.8186 
## 
## Coefficients:
##                Estimate Std. Error t value Pr(>|t|)
## (Intercept)      1.2290     6.8707   0.179    0.860
## ectPro$average  -0.1590     0.1983  -0.802    0.433
## 
## Residual standard error: 1.414 on 18 degrees of freedom
## Multiple R-squared:  0.03449,    Adjusted R-squared:  -0.01915 
## F-statistic: 0.643 on 1 and 18 DF,  p-value: 0.4331
```

```
summary(lm(ectPro$percentBias ~ ectPro$average))
```

```
## 
## Call:
## lm(formula = ectPro$percentBias ~ ectPro$average)
## 
## Residuals:
##     Min      1Q  Median      3Q     Max 
## -9.5757 -1.8996  0.6416  3.4872  5.4328 
## 
## Coefficients:
##                Estimate Std. Error t value Pr(>|t|)
## (Intercept)     -8.4240    19.9388  -0.422    0.678
## ectPro$average  -0.1132     0.5754  -0.197    0.846
## 
## Residual standard error: 4.103 on 18 degrees of freedom
## Multiple R-squared:  0.002145,   Adjusted R-squared:  -0.05329 
## F-statistic: 0.03869 on 1 and 18 DF,  p-value: 0.8463
```

Lastly, generate percentage difference plots for Bison

```
plot(basFro$percentBias ~ basFro$average, ylim = c(-40, 40),
     xlab = "Average of Hand and ImageJ (cm)",
     ylab = "% Difference",
     main = "basFro Percentage Difference")
axis(side = 2, at = yMarks)
abline(h = yMarks, col = "lightgray", lty = "dotted")
```

```
range(basFro$percentBias)
```

```
## [1] -18.776940   3.377949
```

```
mean(basFro$percentBias)
```

```
## [1] -11.81631
```

```
sd(basFro$percentBias)
```

```
## [1] 5.364657
```

```
plot(ectEct$percentBias ~ ectEct$average, ylim = c(-40, 40),
     xlab = "Average of Hand and ImageJ (cm)",
     ylab = "% Difference",
     main = "ectEct Percentage Difference")
axis(side = 2, at = yMarks)
abline(h = yMarks, col = "lightgray", lty = "dotted")
```

```
range(ectEct$percentBias)
```

```
## [1] -13.314238  -4.607403
```

```
mean(ectEct$percentBias)
```

```
## [1] -8.674231
```

```
sd(ectEct$percentBias)
```

```
## [1] 2.618293
```

```
plot(proNas$percentBias ~ proNas$average, ylim = c(-40, 40),
     xlab = "Average of Hand and ImageJ (cm)",
     ylab = "% Difference",
     main = "proNas Percentage Difference")
axis(side = 2, at = yMarks)
abline(h = yMarks, col = "lightgray", lty = "dotted")
```

```
range(proNas$percentBias)
```

```
## [1] -28.485815  -6.287463
```

```
mean(proNas$percentBias)
```

```
## [1] -13.07132
```

```
sd(proNas$percentBias)
```

```
## [1] 5.661342
```

```
plot(nasNuc$percentBias ~ nasNuc$average, ylim = c(-40, 40),
     xlab = "Average of Hand and ImageJ (cm)",
     ylab = "% Difference",
     main = "nasNuc Percentage Difference")
axis(side = 2, at = yMarks)
abline(h = yMarks, col = "lightgray", lty = "dotted")
```

```
range(nasNuc$percentBias)
```

```
## [1] -24.29917 -10.14945
```

```
mean(nasNuc$percentBias)
```

```
## [1] -17.61813
```

```
sd(nasNuc$percentBias)
```

```
## [1] 3.920998
```

```
plot(basTip$percentBias ~ basTip$average, ylim = c(-40, 40),
     xlab = "Average of Hand and ImageJ (cm)",
     ylab = "% Difference",
     main = "basTip Percentage Difference")
axis(side = 2, at = yMarks)
abline(h = yMarks, col = "lightgray", lty = "dotted")
```

```
range(basTip$percentBias)
```

```
## [1] -3.82296 26.88344
```

```
mean(basTip$percentBias)
```

```
## [1] 11.41448
```

```
sd(basTip$percentBias)
```

```
## [1] 8.819831
```

```
plot(ectPro$percentBias ~ ectPro$average, ylim = c(-40, 40),
     xlab = "Average of Hand and ImageJ (cm)",
     ylab = "% Difference",
     main = "ectPro Percentage Difference")
axis(side = 2, at = yMarks)
abline(h = yMarks, col = "lightgray", lty = "dotted")
```

```
range(ectPro$percentBias)
```

```
## [1] -21.776303  -6.768165
```

```
mean(ectPro$percentBias)
```

```
## [1] -12.34158
```

```
sd(ectPro$percentBias)
```

```
## [1] 3.998161
```
