# Supplementary material for: Linear morphometrics fail to support strong sexual dimorphism in Uintatherium anceps
Source: PLoS One. 2026 Oct 7;21(10):e0345236. doi: 10.1371/journal.pone.0345236 (PMC13645082; doi:10.1371/journal.pone.0345236)
Supplement: Supplementary file 12 [file pone.0345236.s026.html]

Simulation Test


# Simulation Test

#### 2026-07-24

set wd and load packages

```
#setwd("")

library(ggplot2)
library(dplyr)
```

```
## 
## Attaching package: 'dplyr'
```

```
## The following objects are masked from 'package:stats':
## 
##     filter, lag
```

```
## The following objects are masked from 'package:base':
## 
##     intersect, setdiff, setequal, union
```

```
library(effectsize)
library(cowplot)
library(gridExtra)
```

```
## 
## Attaching package: 'gridExtra'
```

```
## The following object is masked from 'package:dplyr':
## 
##     combine
```

```
test <- read.csv("Simulation_Measurements.csv")
test$angle <- as.factor(test$angle)
test$scalebarDepth <- as.factor(test$scalebarDepth)
test$zoom <- as.factor(test$zoom)
```

First, visualize and quantify the effects of camera angle and
scalebar placement

```
notZoomed <- test[test$zoom != "zoomed",] #only testing effects of angle and scalebarDepth

#plotting each variable for all unzoomed to see patterns in data
plot(notZoomed$tipFro ~ notZoomed$basFro)
text(notZoomed$basFro, notZoomed$tipFro, labels = notZoomed$Trial, pos=3, cex=0.5)
```

```
plot(notZoomed$tipFro)
text(row_number(notZoomed), notZoomed$tipFro, labels = notZoomed$Trial, pos=3, cex=0.5)
```

```
plot(notZoomed$basFro)
text(row_number(notZoomed), notZoomed$basFro, labels = notZoomed$Trial, pos=3, cex=0.5)
```

```
plot(notZoomed$ectEct)
text(row_number(notZoomed), notZoomed$ectEct, labels = notZoomed$Trial, pos=3, cex=0.5)
```

```
#Alternative plotting technique
#Also use ANOVA to quantify effects of scalebar placement and camera angle

#basFro
basFroPlot <- ggplot(notZoomed) +
  aes(x = scalebarDepth, y = basFro, fill = angle) +
  geom_boxplot() + 
  scale_fill_manual(values = c(
    "#0072B2",  # blue
    "#E69F00",  # orange
    "#009E73"   # green
  ))
basFroTest <- aov(basFro ~ angle*scalebarDepth, data = notZoomed)
summary(basFroTest)
```

```
##                     Df Sum Sq Mean Sq F value   Pr(>F)    
## angle                2   1.81    0.90   3.400   0.0559 .  
## scalebarDepth        2 254.82  127.41 479.712 2.44e-16 ***
## angle:scalebarDepth  4   1.06    0.26   0.995   0.4357    
## Residuals           18   4.78    0.27                     
## ---
## Signif. codes:  0 '***' 0.001 '**' 0.01 '*' 0.05 '.' 0.1 ' ' 1
```

```
eta_squared(basFroTest, partial = TRUE)
```

```
## # Effect Size for ANOVA (Type I)
## 
## Parameter           | Eta2 (partial) |       95% CI
## ---------------------------------------------------
## angle               |           0.27 | [0.00, 1.00]
## scalebarDepth       |           0.98 | [0.96, 1.00]
## angle:scalebarDepth |           0.18 | [0.00, 1.00]
## 
## - One-sided CIs: upper bound fixed at [1.00].
```

```
#ectEct
ectEctPlot <- ggplot(notZoomed) +
  aes(x = scalebarDepth, y = ectEct, fill = angle) +
  geom_boxplot() + 
  scale_fill_manual(values = c(
    "#0072B2",  # blue
    "#E69F00",  # orange
    "#009E73"   # green
  ))
ectEctTest <- aov(ectEct~ angle*scalebarDepth, data = notZoomed)
summary(ectEctTest)
```

```
##                     Df Sum Sq Mean Sq F value Pr(>F)    
## angle                2    0.9    0.44   1.330  0.289    
## scalebarDepth        2  546.2  273.11 833.356 <2e-16 ***
## angle:scalebarDepth  4    1.5    0.38   1.146  0.367    
## Residuals           18    5.9    0.33                   
## ---
## Signif. codes:  0 '***' 0.001 '**' 0.01 '*' 0.05 '.' 0.1 ' ' 1
```

```
eta_squared(ectEctTest, partial = TRUE)
```

```
## # Effect Size for ANOVA (Type I)
## 
## Parameter           | Eta2 (partial) |       95% CI
## ---------------------------------------------------
## angle               |           0.13 | [0.00, 1.00]
## scalebarDepth       |           0.99 | [0.98, 1.00]
## angle:scalebarDepth |           0.20 | [0.00, 1.00]
## 
## - One-sided CIs: upper bound fixed at [1.00].
```

```
#proNas
proNasPlot <- ggplot(notZoomed) +
  aes(x = scalebarDepth, y = proNas, fill = angle) +
  geom_boxplot() + 
  scale_fill_manual(values = c(
    "#0072B2",  # blue
    "#E69F00",  # orange
    "#009E73"   # green
  ))
proNasTest <- aov(proNas~ angle*scalebarDepth, data = notZoomed)
summary(proNasTest)
```

```
##                     Df Sum Sq Mean Sq F value   Pr(>F)    
## angle                2  198.5    99.3 151.293 5.55e-12 ***
## scalebarDepth        2  700.2   350.1 533.641  < 2e-16 ***
## angle:scalebarDepth  4   10.0     2.5   3.801   0.0207 *  
## Residuals           18   11.8     0.7                     
## ---
## Signif. codes:  0 '***' 0.001 '**' 0.01 '*' 0.05 '.' 0.1 ' ' 1
```

```
eta_squared(proNasTest, partial = TRUE)
```

```
## # Effect Size for ANOVA (Type I)
## 
## Parameter           | Eta2 (partial) |       95% CI
## ---------------------------------------------------
## angle               |           0.94 | [0.89, 1.00]
## scalebarDepth       |           0.98 | [0.97, 1.00]
## angle:scalebarDepth |           0.46 | [0.07, 1.00]
## 
## - One-sided CIs: upper bound fixed at [1.00].
```

```
#nasNuc
nasNucPlot <- ggplot(notZoomed) +
  aes(x = scalebarDepth, y = nasNuc, fill = angle) +
  geom_boxplot() + 
  scale_fill_manual(values = c(
    "#0072B2",  # blue
    "#E69F00",  # orange
    "#009E73"   # green
  ))
nasNucTest <- aov(nasNuc~ angle*scalebarDepth, data = notZoomed)
summary(nasNucTest)
```

```
##                     Df Sum Sq Mean Sq F value   Pr(>F)    
## angle                2  95.87   47.93 117.251 4.75e-11 ***
## scalebarDepth        2 217.59  108.79 266.126 4.29e-14 ***
## angle:scalebarDepth  4   8.35    2.09   5.106  0.00629 ** 
## Residuals           18   7.36    0.41                     
## ---
## Signif. codes:  0 '***' 0.001 '**' 0.01 '*' 0.05 '.' 0.1 ' ' 1
```

```
eta_squared(nasNucTest, partial = TRUE)
```

```
## # Effect Size for ANOVA (Type I)
## 
## Parameter           | Eta2 (partial) |       95% CI
## ---------------------------------------------------
## angle               |           0.93 | [0.86, 1.00]
## scalebarDepth       |           0.97 | [0.94, 1.00]
## angle:scalebarDepth |           0.53 | [0.16, 1.00]
## 
## - One-sided CIs: upper bound fixed at [1.00].
```

```
#basTip
basTipPlot <- ggplot(notZoomed) +
  aes(x = scalebarDepth, y = basTip, fill = angle) +
  geom_boxplot() + 
  scale_fill_manual(values = c(
    "#0072B2",  # blue
    "#E69F00",  # orange
    "#009E73"   # green
  ))
basTipTest <- aov(basTip~ angle*scalebarDepth, data = notZoomed)
summary(basTipTest)
```

```
##                     Df Sum Sq Mean Sq F value   Pr(>F)    
## angle                2   6.07    3.04  11.671 0.000562 ***
## scalebarDepth        2 175.16   87.58 336.525 5.52e-15 ***
## angle:scalebarDepth  4   1.28    0.32   1.234 0.331616    
## Residuals           18   4.68    0.26                     
## ---
## Signif. codes:  0 '***' 0.001 '**' 0.01 '*' 0.05 '.' 0.1 ' ' 1
```

```
eta_squared(basTipTest, partial = TRUE)
```

```
## # Effect Size for ANOVA (Type I)
## 
## Parameter           | Eta2 (partial) |       95% CI
## ---------------------------------------------------
## angle               |           0.56 | [0.26, 1.00]
## scalebarDepth       |           0.97 | [0.95, 1.00]
## angle:scalebarDepth |           0.22 | [0.00, 1.00]
## 
## - One-sided CIs: upper bound fixed at [1.00].
```

```
#ectPro
ectProPlot <- ggplot(notZoomed) +
  aes(x = scalebarDepth, y = ectPro, fill = angle) +
  geom_boxplot() + 
  scale_fill_manual(values = c(
    "#0072B2",  # blue
    "#E69F00",  # orange
    "#009E73"   # green
  ))
ectProTest <- aov(ectPro ~ angle*scalebarDepth, data = notZoomed)
summary(ectProTest)
```

```
##                     Df Sum Sq Mean Sq F value   Pr(>F)    
## angle                2  101.2    50.6  111.18 7.41e-11 ***
## scalebarDepth        2 1028.3   514.2 1129.68  < 2e-16 ***
## angle:scalebarDepth  4    6.1     1.5    3.35   0.0323 *  
## Residuals           18    8.2     0.5                     
## ---
## Signif. codes:  0 '***' 0.001 '**' 0.01 '*' 0.05 '.' 0.1 ' ' 1
```

```
eta_squared(ectProTest, partial = TRUE)
```

```
## # Effect Size for ANOVA (Type I)
## 
## Parameter           | Eta2 (partial) |       95% CI
## ---------------------------------------------------
## angle               |           0.93 | [0.86, 1.00]
## scalebarDepth       |           0.99 | [0.98, 1.00]
## angle:scalebarDepth |           0.43 | [0.03, 1.00]
## 
## - One-sided CIs: upper bound fixed at [1.00].
```

```
#basWid
basWidPlot <- ggplot(notZoomed) +
  aes(x = scalebarDepth, y = basWid, fill = angle) +
  geom_boxplot() + 
  scale_fill_manual(values = c(
    "#0072B2",  # blue
    "#E69F00",  # orange
    "#009E73"   # green
  ))
basWidTest <- aov(basWid ~ angle*scalebarDepth, data = notZoomed)
summary(basWidTest)
```

```
##                     Df Sum Sq Mean Sq F value   Pr(>F)    
## angle                2  0.015   0.007   0.248    0.783    
## scalebarDepth        2 19.627   9.813 332.942 6.06e-15 ***
## angle:scalebarDepth  4  0.124   0.031   1.050    0.409    
## Residuals           18  0.531   0.029                     
## ---
## Signif. codes:  0 '***' 0.001 '**' 0.01 '*' 0.05 '.' 0.1 ' ' 1
```

```
eta_squared(basWidTest, partial = TRUE)
```

```
## # Effect Size for ANOVA (Type I)
## 
## Parameter           | Eta2 (partial) |       95% CI
## ---------------------------------------------------
## angle               |           0.03 | [0.00, 1.00]
## scalebarDepth       |           0.97 | [0.95, 1.00]
## angle:scalebarDepth |           0.19 | [0.00, 1.00]
## 
## - One-sided CIs: upper bound fixed at [1.00].
```

```
legend <- get_legend(basWidPlot)

basFroPlot <- basFroPlot + theme(legend.position = "none")
ectEctPlot <- ectEctPlot + theme(legend.position = "none")
proNasPlot <- proNasPlot + theme(legend.position = "none")
nasNucPlot <- nasNucPlot + theme(legend.position = "none")
basTipPlot <- basTipPlot + theme(legend.position = "none")
ectProPlot <- ectProPlot + theme(legend.position = "none")
basWidPlot <- basWidPlot + theme(legend.position = "none")

grid.arrange(basFroPlot, ectEctPlot, proNasPlot, nasNucPlot,
             basTipPlot, ectProPlot, basWidPlot, legend, nrow = 4, ncol = 2)
```

Visualize and quantify the effects of camera zoom

```
zoomTest <- test[test$scalebarDepth == "mid",]

#basFro
basFroZoomPlot <- ggplot(zoomTest) +
  aes(x = zoom, y = basFro, fill = angle) +
  geom_boxplot() + 
  scale_fill_manual(values = c(
    "#0072B2",  # blue
    "#E69F00",  # orange
    "#009E73"   # green
  ))
basFroZoomTest <- aov(basFro ~ angle*zoom, data = zoomTest)
summary(basFroZoomTest)
```

```
##             Df Sum Sq Mean Sq F value Pr(>F)  
## angle        2  0.127   0.064   0.151 0.8611  
## zoom         1  3.681   3.681   8.771 0.0119 *
## angle:zoom   2  0.527   0.263   0.628 0.5505  
## Residuals   12  5.036   0.420                 
## ---
## Signif. codes:  0 '***' 0.001 '**' 0.01 '*' 0.05 '.' 0.1 ' ' 1
```

```
eta_squared(basFroZoomTest, partial = TRUE)
```

```
## # Effect Size for ANOVA (Type I)
## 
## Parameter  | Eta2 (partial) |       95% CI
## ------------------------------------------
## angle      |           0.02 | [0.00, 1.00]
## zoom       |           0.42 | [0.08, 1.00]
## angle:zoom |           0.09 | [0.00, 1.00]
## 
## - One-sided CIs: upper bound fixed at [1.00].
```

```
#ectEct
ectEctZoomPlot <- ggplot(zoomTest) +
  aes(x = zoom, y = ectEct, fill = angle) +
  geom_boxplot() + 
  scale_fill_manual(values = c(
    "#0072B2",  # blue
    "#E69F00",  # orange
    "#009E73"   # green
  ))
ectEctZoomTest <- aov(ectEct ~ angle*zoom, data = zoomTest)
summary(ectEctZoomTest)
```

```
##             Df Sum Sq Mean Sq F value  Pr(>F)   
## angle        2  0.268   0.134   0.327 0.72753   
## zoom         1  5.268   5.268  12.832 0.00377 **
## angle:zoom   2  0.259   0.130   0.316 0.73525   
## Residuals   12  4.926   0.411                   
## ---
## Signif. codes:  0 '***' 0.001 '**' 0.01 '*' 0.05 '.' 0.1 ' ' 1
```

```
eta_squared(ectEctZoomTest, partial = TRUE)
```

```
## # Effect Size for ANOVA (Type I)
## 
## Parameter  | Eta2 (partial) |       95% CI
## ------------------------------------------
## angle      |           0.05 | [0.00, 1.00]
## zoom       |           0.52 | [0.16, 1.00]
## angle:zoom |           0.05 | [0.00, 1.00]
## 
## - One-sided CIs: upper bound fixed at [1.00].
```

```
#proNas
proNasZoomPlot <- ggplot(zoomTest) +
  aes(x = zoom, y = proNas, fill = angle) +
  geom_boxplot() + 
  scale_fill_manual(values = c(
    "#0072B2",  # blue
    "#E69F00",  # orange
    "#009E73"   # green
  ))
proNasZoomTest <- aov(proNas ~ angle*zoom, data = zoomTest)
summary(proNasZoomTest)
```

```
##             Df Sum Sq Mean Sq F value  Pr(>F)    
## angle        2 127.77   63.89  97.484 3.8e-08 ***
## zoom         1  18.84   18.84  28.755 0.00017 ***
## angle:zoom   2   6.95    3.48   5.306 0.02234 *  
## Residuals   12   7.86    0.66                    
## ---
## Signif. codes:  0 '***' 0.001 '**' 0.01 '*' 0.05 '.' 0.1 ' ' 1
```

```
eta_squared(proNasZoomTest, partial = TRUE)
```

```
## # Effect Size for ANOVA (Type I)
## 
## Parameter  | Eta2 (partial) |       95% CI
## ------------------------------------------
## angle      |           0.94 | [0.87, 1.00]
## zoom       |           0.71 | [0.41, 1.00]
## angle:zoom |           0.47 | [0.06, 1.00]
## 
## - One-sided CIs: upper bound fixed at [1.00].
```

```
#nasNuc
nasNucZoomPlot <- ggplot(zoomTest) +
  aes(x = zoom, y = nasNuc, fill = angle) +
  geom_boxplot() + 
  scale_fill_manual(values = c(
    "#0072B2",  # blue
    "#E69F00",  # orange
    "#009E73"   # green
  ))
nasNucZoomTest <- aov(nasNuc ~ angle*zoom, data = zoomTest)
summary(nasNucZoomTest)
```

```
##             Df Sum Sq Mean Sq F value   Pr(>F)    
## angle        2  48.42  24.209  93.003 4.95e-08 ***
## zoom         1   3.67   3.666  14.084  0.00276 ** 
## angle:zoom   2   3.11   1.554   5.971  0.01585 *  
## Residuals   12   3.12   0.260                     
## ---
## Signif. codes:  0 '***' 0.001 '**' 0.01 '*' 0.05 '.' 0.1 ' ' 1
```

```
eta_squared(nasNucZoomTest, partial = TRUE)
```

```
## # Effect Size for ANOVA (Type I)
## 
## Parameter  | Eta2 (partial) |       95% CI
## ------------------------------------------
## angle      |           0.94 | [0.86, 1.00]
## zoom       |           0.54 | [0.18, 1.00]
## angle:zoom |           0.50 | [0.09, 1.00]
## 
## - One-sided CIs: upper bound fixed at [1.00].
```

```
#basTip
basTipZoomPlot <- ggplot(zoomTest) +
  aes(x = zoom, y = basTip, fill = angle) +
  geom_boxplot() + 
  scale_fill_manual(values = c(
    "#0072B2",  # blue
    "#E69F00",  # orange
    "#009E73"   # green
  ))
basTipZoomTest <- aov(basTip ~ angle*zoom, data = zoomTest)
summary(basTipZoomTest)
```

```
##             Df Sum Sq Mean Sq F value Pr(>F)  
## angle        2  3.580   1.790   4.191 0.0416 *
## zoom         1  3.829   3.829   8.966 0.0112 *
## angle:zoom   2  0.021   0.010   0.024 0.9762  
## Residuals   12  5.124   0.427                 
## ---
## Signif. codes:  0 '***' 0.001 '**' 0.01 '*' 0.05 '.' 0.1 ' ' 1
```

```
eta_squared(basTipZoomTest, partial = TRUE)
```

```
## # Effect Size for ANOVA (Type I)
## 
## Parameter  | Eta2 (partial) |       95% CI
## ------------------------------------------
## angle      |           0.41 | [0.01, 1.00]
## zoom       |           0.43 | [0.07, 1.00]
## angle:zoom |       4.01e-03 | [0.00, 1.00]
## 
## - One-sided CIs: upper bound fixed at [1.00].
```

```
#ectPro
ectProZoomPlot <- ggplot(zoomTest) +
  aes(x = zoom, y = ectPro, fill = angle) +
  geom_boxplot() + 
  scale_fill_manual(values = c(
    "#0072B2",  # blue
    "#E69F00",  # orange
    "#009E73"   # green
  ))
ectProZoomTest <- aov(ectPro ~ angle*zoom, data = zoomTest)
summary(ectProZoomTest)
```

```
##             Df Sum Sq Mean Sq F value   Pr(>F)    
## angle        2  69.66   34.83  57.169 7.34e-07 ***
## zoom         1  13.15   13.15  21.589 0.000564 ***
## angle:zoom   2   5.00    2.50   4.105 0.043820 *  
## Residuals   12   7.31    0.61                     
## ---
## Signif. codes:  0 '***' 0.001 '**' 0.01 '*' 0.05 '.' 0.1 ' ' 1
```

```
eta_squared(ectProZoomTest, partial = TRUE)
```

```
## # Effect Size for ANOVA (Type I)
## 
## Parameter  | Eta2 (partial) |       95% CI
## ------------------------------------------
## angle      |           0.91 | [0.78, 1.00]
## zoom       |           0.64 | [0.31, 1.00]
## angle:zoom |           0.41 | [0.01, 1.00]
## 
## - One-sided CIs: upper bound fixed at [1.00].
```

```
#basWid
basWidZoomPlot <- ggplot(zoomTest) +
  aes(x = zoom, y = basWid, fill = angle) +
  geom_boxplot() + 
  scale_fill_manual(values = c(
    "#0072B2",  # blue
    "#E69F00",  # orange
    "#009E73"   # green
  ))
basWidZoomTest <- aov(basWid ~ angle*zoom, data = zoomTest)
summary(basWidZoomTest)
```

```
##             Df Sum Sq Mean Sq F value  Pr(>F)   
## angle        2 0.0919  0.0460   0.735 0.50000   
## zoom         1 0.8308  0.8308  13.282 0.00336 **
## angle:zoom   2 0.0020  0.0010   0.016 0.98424   
## Residuals   12 0.7506  0.0625                   
## ---
## Signif. codes:  0 '***' 0.001 '**' 0.01 '*' 0.05 '.' 0.1 ' ' 1
```

```
eta_squared(basWidZoomTest, partial = TRUE)
```

```
## # Effect Size for ANOVA (Type I)
## 
## Parameter  | Eta2 (partial) |       95% CI
## ------------------------------------------
## angle      |           0.11 | [0.00, 1.00]
## zoom       |           0.53 | [0.17, 1.00]
## angle:zoom |       2.64e-03 | [0.00, 1.00]
## 
## - One-sided CIs: upper bound fixed at [1.00].
```

```
legend <- get_legend(basWidZoomPlot)

basFroZoomPlot <- basFroZoomPlot + theme(legend.position = "none")
ectEctZoomPlot <- ectEctZoomPlot + theme(legend.position = "none")
proNasZoomPlot <- proNasZoomPlot + theme(legend.position = "none")
nasNucZoomPlot <- nasNucZoomPlot + theme(legend.position = "none")
basTipZoomPlot <- basTipZoomPlot + theme(legend.position = "none")
ectProZoomPlot <- ectProZoomPlot + theme(legend.position = "none")
basWidZoomPlot <- basWidZoomPlot + theme(legend.position = "none")

grid.arrange(basFroZoomPlot, ectEctZoomPlot, proNasZoomPlot, nasNucZoomPlot,
             basTipZoomPlot, ectProZoomPlot, basWidZoomPlot, legend, nrow = 4, ncol = 2)
```

Calculate maximum variance within a single treatment (same zoom,
angle, and scalebar placement)

```
angles <- unique(test$angle)
depths <- unique(test$scalebarDepth)
zooms <- unique(test$zoom)
metrics <- colnames(test)[2:9]

ranges <- matrix(nrow=144, ncol = 5)
colnames(ranges) <- c("treatment", "min", "max", "mean", "maxVar")
ranges <- as.data.frame(ranges)

allTreats <- vector(mode = "character", length = 144)
treat <- 1
for (column in 1:length(metrics)) {
  for (i in 1:length(angles)) {
    for (j in 1:length(depths)){
      for (k in 1: length(zooms)){
        values <- paste(metrics[column], angles[i], depths[j], zooms[k])
        print(values)
        allTreats[treat] <- values
        treat <- treat + 1
      }
    }
  }
}
```

```
## [1] "tipFro high close standard"
## [1] "tipFro high close zoomed"
## [1] "tipFro high far standard"
## [1] "tipFro high far zoomed"
## [1] "tipFro high mid standard"
## [1] "tipFro high mid zoomed"
## [1] "tipFro low close standard"
## [1] "tipFro low close zoomed"
## [1] "tipFro low far standard"
## [1] "tipFro low far zoomed"
## [1] "tipFro low mid standard"
## [1] "tipFro low mid zoomed"
## [1] "tipFro mid close standard"
## [1] "tipFro mid close zoomed"
## [1] "tipFro mid far standard"
## [1] "tipFro mid far zoomed"
## [1] "tipFro mid mid standard"
## [1] "tipFro mid mid zoomed"
## [1] "basFro high close standard"
## [1] "basFro high close zoomed"
## [1] "basFro high far standard"
## [1] "basFro high far zoomed"
## [1] "basFro high mid standard"
## [1] "basFro high mid zoomed"
## [1] "basFro low close standard"
## [1] "basFro low close zoomed"
## [1] "basFro low far standard"
## [1] "basFro low far zoomed"
## [1] "basFro low mid standard"
## [1] "basFro low mid zoomed"
## [1] "basFro mid close standard"
## [1] "basFro mid close zoomed"
## [1] "basFro mid far standard"
## [1] "basFro mid far zoomed"
## [1] "basFro mid mid standard"
## [1] "basFro mid mid zoomed"
## [1] "ectEct high close standard"
## [1] "ectEct high close zoomed"
## [1] "ectEct high far standard"
## [1] "ectEct high far zoomed"
## [1] "ectEct high mid standard"
## [1] "ectEct high mid zoomed"
## [1] "ectEct low close standard"
## [1] "ectEct low close zoomed"
## [1] "ectEct low far standard"
## [1] "ectEct low far zoomed"
## [1] "ectEct low mid standard"
## [1] "ectEct low mid zoomed"
## [1] "ectEct mid close standard"
## [1] "ectEct mid close zoomed"
## [1] "ectEct mid far standard"
## [1] "ectEct mid far zoomed"
## [1] "ectEct mid mid standard"
## [1] "ectEct mid mid zoomed"
## [1] "proNas high close standard"
## [1] "proNas high close zoomed"
## [1] "proNas high far standard"
## [1] "proNas high far zoomed"
## [1] "proNas high mid standard"
## [1] "proNas high mid zoomed"
## [1] "proNas low close standard"
## [1] "proNas low close zoomed"
## [1] "proNas low far standard"
## [1] "proNas low far zoomed"
## [1] "proNas low mid standard"
## [1] "proNas low mid zoomed"
## [1] "proNas mid close standard"
## [1] "proNas mid close zoomed"
## [1] "proNas mid far standard"
## [1] "proNas mid far zoomed"
## [1] "proNas mid mid standard"
## [1] "proNas mid mid zoomed"
## [1] "nasNuc high close standard"
## [1] "nasNuc high close zoomed"
## [1] "nasNuc high far standard"
## [1] "nasNuc high far zoomed"
## [1] "nasNuc high mid standard"
## [1] "nasNuc high mid zoomed"
## [1] "nasNuc low close standard"
## [1] "nasNuc low close zoomed"
## [1] "nasNuc low far standard"
## [1] "nasNuc low far zoomed"
## [1] "nasNuc low mid standard"
## [1] "nasNuc low mid zoomed"
## [1] "nasNuc mid close standard"
## [1] "nasNuc mid close zoomed"
## [1] "nasNuc mid far standard"
## [1] "nasNuc mid far zoomed"
## [1] "nasNuc mid mid standard"
## [1] "nasNuc mid mid zoomed"
## [1] "basTip high close standard"
## [1] "basTip high close zoomed"
## [1] "basTip high far standard"
## [1] "basTip high far zoomed"
## [1] "basTip high mid standard"
## [1] "basTip high mid zoomed"
## [1] "basTip low close standard"
## [1] "basTip low close zoomed"
## [1] "basTip low far standard"
## [1] "basTip low far zoomed"
## [1] "basTip low mid standard"
## [1] "basTip low mid zoomed"
## [1] "basTip mid close standard"
## [1] "basTip mid close zoomed"
## [1] "basTip mid far standard"
## [1] "basTip mid far zoomed"
## [1] "basTip mid mid standard"
## [1] "basTip mid mid zoomed"
## [1] "ectPro high close standard"
## [1] "ectPro high close zoomed"
## [1] "ectPro high far standard"
## [1] "ectPro high far zoomed"
## [1] "ectPro high mid standard"
## [1] "ectPro high mid zoomed"
## [1] "ectPro low close standard"
## [1] "ectPro low close zoomed"
## [1] "ectPro low far standard"
## [1] "ectPro low far zoomed"
## [1] "ectPro low mid standard"
## [1] "ectPro low mid zoomed"
## [1] "ectPro mid close standard"
## [1] "ectPro mid close zoomed"
## [1] "ectPro mid far standard"
## [1] "ectPro mid far zoomed"
## [1] "ectPro mid mid standard"
## [1] "ectPro mid mid zoomed"
## [1] "basWid high close standard"
## [1] "basWid high close zoomed"
## [1] "basWid high far standard"
## [1] "basWid high far zoomed"
## [1] "basWid high mid standard"
## [1] "basWid high mid zoomed"
## [1] "basWid low close standard"
## [1] "basWid low close zoomed"
## [1] "basWid low far standard"
## [1] "basWid low far zoomed"
## [1] "basWid low mid standard"
## [1] "basWid low mid zoomed"
## [1] "basWid mid close standard"
## [1] "basWid mid close zoomed"
## [1] "basWid mid far standard"
## [1] "basWid mid far zoomed"
## [1] "basWid mid mid standard"
## [1] "basWid mid mid zoomed"
```

```
ranges$treatment <- allTreats
rows <- 1

for (met in 1:length(metrics)) {
  for (i in 1:length(angles)) {
    for (j in 1:length(depths)){
      for (k in 1: length(zooms)){
        column <- test[,colnames(test) == metrics[met]]
        values <- column[test$angle == levels(angles)[i] 
                          & test$scalebarDepth == levels(depths)[j]  
                          & test$zoom == levels(zooms)[k]]
        if (is.numeric(values)) {
          ranges[rows,]$min <- min(values)
          ranges[rows,]$max <- max(values)
          ranges[rows,]$mean <- mean(values)
          ranges[rows,]$maxVar <- ((max(values)/min(values))*100)-100
        }
        rows <- rows+1
      }
    }
  }
}
```

```
## Warning in min(values): no non-missing arguments to min; returning Inf
```

```
## Warning in max(values): no non-missing arguments to max; returning -Inf
## Warning in max(values): no non-missing arguments to max; returning -Inf
```

```
## Warning in min(values): no non-missing arguments to min; returning Inf
## Warning in min(values): no non-missing arguments to min; returning Inf
```

```
## Warning in max(values): no non-missing arguments to max; returning -Inf
## Warning in max(values): no non-missing arguments to max; returning -Inf
```

```
## Warning in min(values): no non-missing arguments to min; returning Inf
## Warning in min(values): no non-missing arguments to min; returning Inf
```

```
## Warning in max(values): no non-missing arguments to max; returning -Inf
## Warning in max(values): no non-missing arguments to max; returning -Inf
```

```
## Warning in min(values): no non-missing arguments to min; returning Inf
## Warning in min(values): no non-missing arguments to min; returning Inf
```

```
## Warning in max(values): no non-missing arguments to max; returning -Inf
## Warning in max(values): no non-missing arguments to max; returning -Inf
```

```
## Warning in min(values): no non-missing arguments to min; returning Inf
## Warning in min(values): no non-missing arguments to min; returning Inf
```

```
## Warning in max(values): no non-missing arguments to max; returning -Inf
## Warning in max(values): no non-missing arguments to max; returning -Inf
```

```
## Warning in min(values): no non-missing arguments to min; returning Inf
## Warning in min(values): no non-missing arguments to min; returning Inf
```

```
## Warning in max(values): no non-missing arguments to max; returning -Inf
## Warning in max(values): no non-missing arguments to max; returning -Inf
```

```
## Warning in min(values): no non-missing arguments to min; returning Inf
## Warning in min(values): no non-missing arguments to min; returning Inf
```

```
## Warning in max(values): no non-missing arguments to max; returning -Inf
## Warning in max(values): no non-missing arguments to max; returning -Inf
```

```
## Warning in min(values): no non-missing arguments to min; returning Inf
## Warning in min(values): no non-missing arguments to min; returning Inf
```

```
## Warning in max(values): no non-missing arguments to max; returning -Inf
## Warning in max(values): no non-missing arguments to max; returning -Inf
```

```
## Warning in min(values): no non-missing arguments to min; returning Inf
## Warning in min(values): no non-missing arguments to min; returning Inf
```

```
## Warning in max(values): no non-missing arguments to max; returning -Inf
## Warning in max(values): no non-missing arguments to max; returning -Inf
```

```
## Warning in min(values): no non-missing arguments to min; returning Inf
## Warning in min(values): no non-missing arguments to min; returning Inf
```

```
## Warning in max(values): no non-missing arguments to max; returning -Inf
## Warning in max(values): no non-missing arguments to max; returning -Inf
```

```
## Warning in min(values): no non-missing arguments to min; returning Inf
## Warning in min(values): no non-missing arguments to min; returning Inf
```

```
## Warning in max(values): no non-missing arguments to max; returning -Inf
## Warning in max(values): no non-missing arguments to max; returning -Inf
```

```
## Warning in min(values): no non-missing arguments to min; returning Inf
## Warning in min(values): no non-missing arguments to min; returning Inf
```

```
## Warning in max(values): no non-missing arguments to max; returning -Inf
## Warning in max(values): no non-missing arguments to max; returning -Inf
```

```
## Warning in min(values): no non-missing arguments to min; returning Inf
## Warning in min(values): no non-missing arguments to min; returning Inf
```

```
## Warning in max(values): no non-missing arguments to max; returning -Inf
## Warning in max(values): no non-missing arguments to max; returning -Inf
```

```
## Warning in min(values): no non-missing arguments to min; returning Inf
## Warning in min(values): no non-missing arguments to min; returning Inf
```

```
## Warning in max(values): no non-missing arguments to max; returning -Inf
## Warning in max(values): no non-missing arguments to max; returning -Inf
```

```
## Warning in min(values): no non-missing arguments to min; returning Inf
## Warning in min(values): no non-missing arguments to min; returning Inf
```

```
## Warning in max(values): no non-missing arguments to max; returning -Inf
## Warning in max(values): no non-missing arguments to max; returning -Inf
```

```
## Warning in min(values): no non-missing arguments to min; returning Inf
## Warning in min(values): no non-missing arguments to min; returning Inf
```

```
## Warning in max(values): no non-missing arguments to max; returning -Inf
## Warning in max(values): no non-missing arguments to max; returning -Inf
```

```
## Warning in min(values): no non-missing arguments to min; returning Inf
## Warning in min(values): no non-missing arguments to min; returning Inf
```

```
## Warning in max(values): no non-missing arguments to max; returning -Inf
## Warning in max(values): no non-missing arguments to max; returning -Inf
```

```
## Warning in min(values): no non-missing arguments to min; returning Inf
## Warning in min(values): no non-missing arguments to min; returning Inf
```

```
## Warning in max(values): no non-missing arguments to max; returning -Inf
## Warning in max(values): no non-missing arguments to max; returning -Inf
```

```
## Warning in min(values): no non-missing arguments to min; returning Inf
## Warning in min(values): no non-missing arguments to min; returning Inf
```

```
## Warning in max(values): no non-missing arguments to max; returning -Inf
## Warning in max(values): no non-missing arguments to max; returning -Inf
```

```
## Warning in min(values): no non-missing arguments to min; returning Inf
## Warning in min(values): no non-missing arguments to min; returning Inf
```

```
## Warning in max(values): no non-missing arguments to max; returning -Inf
## Warning in max(values): no non-missing arguments to max; returning -Inf
```

```
## Warning in min(values): no non-missing arguments to min; returning Inf
## Warning in min(values): no non-missing arguments to min; returning Inf
```

```
## Warning in max(values): no non-missing arguments to max; returning -Inf
## Warning in max(values): no non-missing arguments to max; returning -Inf
```

```
## Warning in min(values): no non-missing arguments to min; returning Inf
## Warning in min(values): no non-missing arguments to min; returning Inf
```

```
## Warning in max(values): no non-missing arguments to max; returning -Inf
## Warning in max(values): no non-missing arguments to max; returning -Inf
```

```
## Warning in min(values): no non-missing arguments to min; returning Inf
## Warning in min(values): no non-missing arguments to min; returning Inf
```

```
## Warning in max(values): no non-missing arguments to max; returning -Inf
## Warning in max(values): no non-missing arguments to max; returning -Inf
```

```
## Warning in min(values): no non-missing arguments to min; returning Inf
## Warning in min(values): no non-missing arguments to min; returning Inf
```

```
## Warning in max(values): no non-missing arguments to max; returning -Inf
## Warning in max(values): no non-missing arguments to max; returning -Inf
```

```
## Warning in min(values): no non-missing arguments to min; returning Inf
## Warning in min(values): no non-missing arguments to min; returning Inf
```

```
## Warning in max(values): no non-missing arguments to max; returning -Inf
## Warning in max(values): no non-missing arguments to max; returning -Inf
```

```
## Warning in min(values): no non-missing arguments to min; returning Inf
## Warning in min(values): no non-missing arguments to min; returning Inf
```

```
## Warning in max(values): no non-missing arguments to max; returning -Inf
## Warning in max(values): no non-missing arguments to max; returning -Inf
```

```
## Warning in min(values): no non-missing arguments to min; returning Inf
## Warning in min(values): no non-missing arguments to min; returning Inf
```

```
## Warning in max(values): no non-missing arguments to max; returning -Inf
## Warning in max(values): no non-missing arguments to max; returning -Inf
```

```
## Warning in min(values): no non-missing arguments to min; returning Inf
## Warning in min(values): no non-missing arguments to min; returning Inf
```

```
## Warning in max(values): no non-missing arguments to max; returning -Inf
## Warning in max(values): no non-missing arguments to max; returning -Inf
```

```
## Warning in min(values): no non-missing arguments to min; returning Inf
## Warning in min(values): no non-missing arguments to min; returning Inf
```

```
## Warning in max(values): no non-missing arguments to max; returning -Inf
## Warning in max(values): no non-missing arguments to max; returning -Inf
```

```
## Warning in min(values): no non-missing arguments to min; returning Inf
## Warning in min(values): no non-missing arguments to min; returning Inf
```

```
## Warning in max(values): no non-missing arguments to max; returning -Inf
## Warning in max(values): no non-missing arguments to max; returning -Inf
```

```
## Warning in min(values): no non-missing arguments to min; returning Inf
## Warning in min(values): no non-missing arguments to min; returning Inf
```

```
## Warning in max(values): no non-missing arguments to max; returning -Inf
## Warning in max(values): no non-missing arguments to max; returning -Inf
```

```
## Warning in min(values): no non-missing arguments to min; returning Inf
## Warning in min(values): no non-missing arguments to min; returning Inf
```

```
## Warning in max(values): no non-missing arguments to max; returning -Inf
## Warning in max(values): no non-missing arguments to max; returning -Inf
```

```
## Warning in min(values): no non-missing arguments to min; returning Inf
## Warning in min(values): no non-missing arguments to min; returning Inf
```

```
## Warning in max(values): no non-missing arguments to max; returning -Inf
## Warning in max(values): no non-missing arguments to max; returning -Inf
```

```
## Warning in min(values): no non-missing arguments to min; returning Inf
## Warning in min(values): no non-missing arguments to min; returning Inf
```

```
## Warning in max(values): no non-missing arguments to max; returning -Inf
## Warning in max(values): no non-missing arguments to max; returning -Inf
```

```
## Warning in min(values): no non-missing arguments to min; returning Inf
## Warning in min(values): no non-missing arguments to min; returning Inf
```

```
## Warning in max(values): no non-missing arguments to max; returning -Inf
## Warning in max(values): no non-missing arguments to max; returning -Inf
```

```
## Warning in min(values): no non-missing arguments to min; returning Inf
## Warning in min(values): no non-missing arguments to min; returning Inf
```

```
## Warning in max(values): no non-missing arguments to max; returning -Inf
## Warning in max(values): no non-missing arguments to max; returning -Inf
```

```
## Warning in min(values): no non-missing arguments to min; returning Inf
## Warning in min(values): no non-missing arguments to min; returning Inf
```

```
## Warning in max(values): no non-missing arguments to max; returning -Inf
## Warning in max(values): no non-missing arguments to max; returning -Inf
```

```
## Warning in min(values): no non-missing arguments to min; returning Inf
## Warning in min(values): no non-missing arguments to min; returning Inf
```

```
## Warning in max(values): no non-missing arguments to max; returning -Inf
## Warning in max(values): no non-missing arguments to max; returning -Inf
```

```
## Warning in min(values): no non-missing arguments to min; returning Inf
## Warning in min(values): no non-missing arguments to min; returning Inf
```

```
## Warning in max(values): no non-missing arguments to max; returning -Inf
## Warning in max(values): no non-missing arguments to max; returning -Inf
```

```
## Warning in min(values): no non-missing arguments to min; returning Inf
## Warning in min(values): no non-missing arguments to min; returning Inf
```

```
## Warning in max(values): no non-missing arguments to max; returning -Inf
## Warning in max(values): no non-missing arguments to max; returning -Inf
```

```
## Warning in min(values): no non-missing arguments to min; returning Inf
## Warning in min(values): no non-missing arguments to min; returning Inf
```

```
## Warning in max(values): no non-missing arguments to max; returning -Inf
## Warning in max(values): no non-missing arguments to max; returning -Inf
```

```
## Warning in min(values): no non-missing arguments to min; returning Inf
## Warning in min(values): no non-missing arguments to min; returning Inf
```

```
## Warning in max(values): no non-missing arguments to max; returning -Inf
## Warning in max(values): no non-missing arguments to max; returning -Inf
```

```
## Warning in min(values): no non-missing arguments to min; returning Inf
## Warning in min(values): no non-missing arguments to min; returning Inf
```

```
## Warning in max(values): no non-missing arguments to max; returning -Inf
## Warning in max(values): no non-missing arguments to max; returning -Inf
```

```
## Warning in min(values): no non-missing arguments to min; returning Inf
## Warning in min(values): no non-missing arguments to min; returning Inf
```

```
## Warning in max(values): no non-missing arguments to max; returning -Inf
## Warning in max(values): no non-missing arguments to max; returning -Inf
```

```
## Warning in min(values): no non-missing arguments to min; returning Inf
## Warning in min(values): no non-missing arguments to min; returning Inf
```

```
## Warning in max(values): no non-missing arguments to max; returning -Inf
## Warning in max(values): no non-missing arguments to max; returning -Inf
```

```
## Warning in min(values): no non-missing arguments to min; returning Inf
## Warning in min(values): no non-missing arguments to min; returning Inf
```

```
## Warning in max(values): no non-missing arguments to max; returning -Inf
## Warning in max(values): no non-missing arguments to max; returning -Inf
```

```
## Warning in min(values): no non-missing arguments to min; returning Inf
## Warning in min(values): no non-missing arguments to min; returning Inf
```

```
## Warning in max(values): no non-missing arguments to max; returning -Inf
## Warning in max(values): no non-missing arguments to max; returning -Inf
```

```
## Warning in min(values): no non-missing arguments to min; returning Inf
## Warning in min(values): no non-missing arguments to min; returning Inf
```

```
## Warning in max(values): no non-missing arguments to max; returning -Inf
## Warning in max(values): no non-missing arguments to max; returning -Inf
```

```
## Warning in min(values): no non-missing arguments to min; returning Inf
```

```
max(ranges$maxVar[!is.na(ranges$maxVar)])
```

```
## [1] 13.48814
```

```
mean(ranges$maxVar[!is.na(ranges$maxVar)])
```

```
## [1] 5.283283
```

Lastly, run analyses performed in study allowing for 13.5% error for
each measurment (maximum observed in a single treatment in
simulations)

Set wd and load packages

```
#setwd("")

library(dplyr)
library(diptest)
library(Amelia)
```

```
## Loading required package: Rcpp
```

```
## ## 
## ## Amelia II: Multiple Imputation
## ## (Version 1.8.3, built: 2024-11-07)
## ## Copyright (C) 2005-2026 James Honaker, Gary King and Matthew Blackwell
## ## Refer to http://gking.harvard.edu/amelia/ for more information
## ##
```

```
library(cluster)
library(ggfortify)
library(ggplot2)
library(ggforce)
library(factoextra)
```

```
## Welcome to factoextra!
```

```
## Want to learn more? See two factoextra-related books at https://www.datanovia.com/en/product/practical-guide-to-principal-component-methods-in-r/
```

```
library(FactoMineR)
library(missMDA)
library(VIM)
```

```
## Loading required package: colorspace
```

```
## Loading required package: grid
```

```
## Registered S3 method overwritten by 'car':
##   method           from
##   na.action.merMod lme4
```

```
## VIM is ready to use.
```

```
## Suggestions and bug-reports can be submitted at: https://github.com/statistikat/VIM/issues
```

```
## 
## Attaching package: 'VIM'
```

```
## The following object is masked from 'package:datasets':
## 
##     sleep
```

```
library(cowplot)
library(plyr)
```

```
## ------------------------------------------------------------------------------
```

```
## You have loaded plyr after dplyr - this is likely to cause problems.
## If you need functions from both plyr and dplyr, please load plyr first, then dplyr:
## library(plyr); library(dplyr)
```

```
## ------------------------------------------------------------------------------
```

```
## 
## Attaching package: 'plyr'
```

```
## The following objects are masked from 'package:dplyr':
## 
##     arrange, count, desc, mutate, rename, summarise, summarize
```

```
library(ggrepel)
library(gridExtra)
library(mclust)
```

```
## Package 'mclust' version 6.1.3
## Type 'citation("mclust")' for citing this R package in publications.
```

```
## 
## Attaching package: 'mclust'
```

```
## The following object is masked from 'package:plyr':
## 
##     count
```

```
## The following object is masked from 'package:VIM':
## 
##     diabetes
```

```
## The following object is masked from 'package:dplyr':
## 
##     count
```

```
library(GGally)
library(BayesFactor)
```

```
## Loading required package: coda
```

```
## Loading required package: Matrix
```

```
## ************
## Welcome to BayesFactor 0.9.12-4.8. If you have questions, please contact Richard Morey (richarddmorey@gmail.com).
## 
## Type BFManual() to open the manual.
## ************
```

```
library(pheatmap)
library(ComplexHeatmap)
```

```
## ========================================
## ComplexHeatmap version 2.24.1
## Bioconductor page: http://bioconductor.org/packages/ComplexHeatmap/
## Github page: https://github.com/jokergoo/ComplexHeatmap
## Documentation: http://jokergoo.github.io/ComplexHeatmap-reference
## 
## If you use it in published research, please cite either one:
## - Gu, Z. Complex Heatmap Visualization. iMeta 2022.
## - Gu, Z. Complex heatmaps reveal patterns and correlations in multidimensional 
##     genomic data. Bioinformatics 2016.
## 
## 
## The new InteractiveComplexHeatmap package can directly export static 
## complex heatmaps into an interactive Shiny app with zero effort. Have a try!
## 
## This message can be suppressed by:
##   suppressPackageStartupMessages(library(ComplexHeatmap))
## ========================================
## ! pheatmap() has been masked by ComplexHeatmap::pheatmap(). Most of the arguments
##    in the original pheatmap() are identically supported in the new function. You 
##    can still use the original function by explicitly calling pheatmap::pheatmap().
```

```
## 
## Attaching package: 'ComplexHeatmap'
```

```
## The following object is masked from 'package:pheatmap':
## 
##     pheatmap
```

```
## The following object is masked from 'package:VIM':
## 
##     prepare
```

```
library(circlize)
```

```
## ========================================
## circlize version 0.4.18
## CRAN page: https://cran.r-project.org/package=circlize
## Github page: https://github.com/jokergoo/circlize
## Documentation: https://jokergoo.github.io/circlize_book/book/
## 
## If you use it in published research, please cite:
## Gu, Z. circlize implements and enhances circular visualization
##   in R. Bioinformatics 2014.
## 
## This message can be suppressed by:
##   suppressPackageStartupMessages(library(circlize))
## ========================================
```

```
library(mixtools)
```

```
## mixtools package, version 2.0.0.1, Released 2022-12-04
## This package is based upon work supported by the National Science Foundation under Grant No. SES-0518772 and the Chan Zuckerberg Initiative: Essential Open Source Software for Science (Grant No. 2020-255193).
```

```
## 
## Attaching package: 'mixtools'
```

```
## The following object is masked from 'package:mclust':
## 
##     dmvnorm
```

```
## The following object is masked from 'package:grid':
## 
##     depth
```

```
library(patchwork)
```

```
## 
## Attaching package: 'patchwork'
```

```
## The following object is masked from 'package:cowplot':
## 
##     align_plots
```

```
library(EnvStats)
```

```
## 
## Attaching package: 'EnvStats'
```

```
## The following object is masked from 'package:Matrix':
## 
##     print
```

```
## The following objects are masked from 'package:stats':
## 
##     predict, predict.lm
```

```
## The following object is masked from 'package:base':
## 
##     print.default
```

```
library(dimorph)
library(effectsize)
library(pvclust)
library(ggnewscale)
library(MASS)
```

```
## 
## Attaching package: 'MASS'
```

```
## The following object is masked from 'package:EnvStats':
## 
##     boxcox
```

```
## The following object is masked from 'package:patchwork':
## 
##     area
```

```
## The following object is masked from 'package:dplyr':
## 
##     select
```

Load and manipulate data

```
Uintatheres <- read.csv("Uintathere_Measurements.csv", check.names = F) #load dataframe
Uintatheres <- Uintatheres[Uintatheres$Specimen != "YPM VP 011039L",] #remove damaged view of YPM 11039
rownames(Uintatheres) <- Uintatheres[,1] #rename rows as specimen numbers
uinFem <- Uintatheres$Specimen[Uintatheres$sex=="F"]
uinMale <- Uintatheres$Specimen[Uintatheres$sex=="M"]
uinNB <- Uintatheres$Specimen[Uintatheres$sex=="?"]
Uintatheres <- Uintatheres[,4:13] #remove helper variables
#Uintatheres[1,]$alvCan <- NA #This canine is potentially pathologic so it's inclusion is questionable

str(Uintatheres)
```

```
## 'data.frame':    26 obs. of  10 variables:
##  $ nasOcc: num  73.4 65.6 71.4 72.7 75.4 ...
##  $ nasCon: num  73.9 60.2 69.1 67.5 71.7 ...
##  $ preCon: num  68.9 56.9 NA 62.3 66.6 ...
##  $ occCon: num  26.7 22.5 24.8 27.6 30.3 ...
##  $ parPro: num  32.7 37.5 43.2 NA 42.6 ...
##  $ maxAlv: num  19.1 24.3 21.4 28.4 30.5 ...
##  $ maxCan: num  26.7 NA 37 49.9 45.6 ...
##  $ alvCan: num  8.49 NA NA 22.07 NA ...
##  $ diaLen: num  7.92 5.58 7.2 9.08 8.81 ...
##  $ canWid: num  4.75 5.36 5.39 7.11 7.09 ...
```

```
colnames(Uintatheres)
```

```
##  [1] "nasOcc" "nasCon" "preCon" "occCon" "parPro" "maxAlv" "maxCan" "alvCan"
##  [9] "diaLen" "canWid"
```

```
set.seed(123)

Uintatheres <- Uintatheres * runif(length(unlist(Uintatheres)), 0.865, 1.135)
```

PART 1: Generate histograms for each variable and test for normality
and unimodality

Setting up “Stats” dataframe to store values of various test
statistics of each measurement

```
columns <- colnames(Uintatheres)
columns <- c("Length from Nasals to Occipital Crest", 
             "Length from Nasals to Occipital Condyles", 
             "Length from Premaxillaries to Occipital Condyles", 
             "Height from Occipital Crest to Occipital Condyles",
             "Height from Parietal Protuberance to tip of Postglenoid Process",
             "Height of Maxillary protuberance from Canine Alveolus", 
             #             "Height from Maxillary Protuberance to Tip of Canine", #For now, I have commented out this measurement
             "Length of Canine from Alveolus to Tip",
             "Diastemal Length",
             "Canine Width at Base")
NVariables <- length(columns)

Uintatheres <- Uintatheres[,-7] #Remove old variable

Measurement <- vector()
n <- vector()
wStatistic <- vector()
pValue <- vector()
dipStatistic <- vector() 

Stats <- data.frame(Measurement, n, wStatistic, pValue, dipStatistic, pValue)
```

RUN for-loop that plots histograms for each variable, runs
Shapiro-Wilk and Hartigan’s Dip Test, and stores these variables in
“Stats” dataframe.

```
for (i in 1:NVariables) {
  histi <- hist(Uintatheres[,i][!is.na(Uintatheres[,i])], main = paste(columns[i]),
                col = "skyblue1", xlab = columns[i], cex.main = 2, cex.lab=1.5)
  lengthi <- unique(Uintatheres[,i][!is.na(Uintatheres[,i])])
  xfit <- seq(min(Uintatheres[,i][!is.na(Uintatheres[,i])], na.rm = T), 
              max(Uintatheres[,i][!is.na(Uintatheres[,i])], na.rm = T), 
              length = length(lengthi)) 
  yfit <- dnorm(xfit, mean = mean(Uintatheres[,i][!is.na(Uintatheres[,i])], na.rm = T), 
                sd = sd(Uintatheres[,i][!is.na(Uintatheres[,i])], na.rm = T)) 
  yfit <- yfit * diff(histi$mids[1:2]) * length(Uintatheres[,i][!is.na(Uintatheres[,i])]) 
  lines(xfit, yfit, col = "red", lwd = 3)
  Stats[i,1] <- columns[i]
  Stats[i,2] <- length(Uintatheres[,i][!is.na(Uintatheres[,i])])
  Stats[i,3] <- shapiro.test(Uintatheres[,i])[1]
  Stats[i,4] <- shapiro.test(Uintatheres[,i])[2]
  Stats[i,5] <- dip.test(Uintatheres[,i])[1]
  Stats[i,6] <- dip.test(Uintatheres[,i])[2]
  mtext(paste("w-statistic: ", Stats[i,3], "; p-value: ", Stats[i,4], "\n",
              "dip-statistic: ", Stats[i,5], "; p-value: ", Stats[i,6],  sep = ""), 
        padj = .3, side = 4, cex = 2)
}
```

```
par(mfrow = c(1,1))
```

Generate Violin Plots for Each Measurement

```
par(mfrow = c(3,3))
par(mar=c(2,2,2,2))

dims <- list(length(colnames(Uintatheres)))
dims[[9]] <- ggplot(data = Uintatheres,
                    mapping = aes(x="", y= `nasals to occipital crest` )) +
  geom_violin( fill = "gray80", trim = F) + geom_point() + geom_boxplot(width = 0.1, fill = "gray70") +
  labs(y = "Length (cm)", x="", title = "Nasals to Occipital Crests")


for (i in 1:length(colnames(Uintatheres))) {
  dims[[i]] <-  ggplot(data = Uintatheres,
                       mapping = aes(x="", y=.data[[colnames(Uintatheres)[i]]])) +
    geom_violin( fill = "gray80", trim = F) + geom_point() + geom_boxplot(width = 0.1, fill = "gray70") +
    labs(y = "Length (cm)", x="", title = colnames(Uintatheres)[i])
}


grid.arrange(dims[[1]], dims[[2]], dims[[3]], dims[[4]], dims[[5]], dims[[6]], dims[[7]], dims[[8]], dims[[9]],
             ncol = 3, nrow = 3)
```

```
## Warning: Removed 2 rows containing non-finite outside the scale range
## (`stat_ydensity()`).
```

```
## Warning: Removed 2 rows containing non-finite outside the scale range
## (`stat_boxplot()`).
```

```
## Warning: Removed 2 rows containing missing values or values outside the scale range
## (`geom_point()`).
```

```
## Warning: Removed 7 rows containing non-finite outside the scale range
## (`stat_ydensity()`).
```

```
## Warning: Removed 7 rows containing non-finite outside the scale range
## (`stat_boxplot()`).
```

```
## Warning: Removed 7 rows containing missing values or values outside the scale range
## (`geom_point()`).
```

```
## Warning: Removed 12 rows containing non-finite outside the scale range
## (`stat_ydensity()`).
```

```
## Warning: Removed 12 rows containing non-finite outside the scale range
## (`stat_boxplot()`).
```

```
## Warning: Removed 12 rows containing missing values or values outside the scale range
## (`geom_point()`).
```

```
## Warning: Removed 7 rows containing non-finite outside the scale range
## (`stat_ydensity()`).
```

```
## Warning: Removed 7 rows containing non-finite outside the scale range
## (`stat_boxplot()`).
```

```
## Warning: Removed 7 rows containing missing values or values outside the scale range
## (`geom_point()`).
```

```
## Warning: Removed 7 rows containing non-finite outside the scale range
## (`stat_ydensity()`).
```

```
## Warning: Removed 7 rows containing non-finite outside the scale range
## (`stat_boxplot()`).
```

```
## Warning: Removed 7 rows containing missing values or values outside the scale range
## (`geom_point()`).
```

```
## Warning: Removed 5 rows containing non-finite outside the scale range
## (`stat_ydensity()`).
```

```
## Warning: Removed 5 rows containing non-finite outside the scale range
## (`stat_boxplot()`).
```

```
## Warning: Removed 5 rows containing missing values or values outside the scale range
## (`geom_point()`).
```

```
## Warning: Removed 16 rows containing non-finite outside the scale range
## (`stat_ydensity()`).
```

```
## Warning: Removed 16 rows containing non-finite outside the scale range
## (`stat_boxplot()`).
```

```
## Warning: Removed 16 rows containing missing values or values outside the scale range
## (`geom_point()`).
```

```
## Warning: Removed 7 rows containing non-finite outside the scale range
## (`stat_ydensity()`).
```

```
## Warning: Removed 7 rows containing non-finite outside the scale range
## (`stat_boxplot()`).
```

```
## Warning: Removed 7 rows containing missing values or values outside the scale range
## (`geom_point()`).
```

```
## Warning: Removed 9 rows containing non-finite outside the scale range
## (`stat_ydensity()`).
```

```
## Warning: Removed 9 rows containing non-finite outside the scale range
## (`stat_boxplot()`).
```

```
## Warning: Removed 9 rows containing missing values or values outside the scale range
## (`geom_point()`).
```

Generate random normal data for reference

```
set.seed(123) #generating random normal data for reference

MyMin <- min(Uintatheres$parPro, na.rm = T)
MyMax <- max(Uintatheres$parPro, na.rm = T)
MyMean <- mean(Uintatheres$parPro, na.rm = T)
MyVar <- var(Uintatheres$parPro, na.rm = T)
MySD <- sqrt(MyVar)
MyNorm <- rnorm(19, mean = MyMean, sd = MySD)
MyData <- data.frame(random = 1:19)
MyData$random <- MyNorm

random <- ggplot(data = MyData,
                 mapping = aes(x="", y= `random` )) +
  geom_violin( fill = "gray80", trim = F) + geom_point() + geom_boxplot(width = 0.1, fill = "gray70") +
  labs(y = "Length (cm)", x="", title = "Random Normal Data")

#plot with random normal data

grid.arrange(dims[[1]], dims[[2]], dims[[3]], dims[[4]], dims[[5]], dims[[6]], dims[[7]], dims[[8]], dims[[9]], random,
             ncol = 2, nrow = 5)
```

```
## Warning: Removed 2 rows containing non-finite outside the scale range
## (`stat_ydensity()`).
```

```
## Warning: Removed 2 rows containing non-finite outside the scale range
## (`stat_boxplot()`).
```

```
## Warning: Removed 2 rows containing missing values or values outside the scale range
## (`geom_point()`).
```

```
## Warning: Removed 7 rows containing non-finite outside the scale range
## (`stat_ydensity()`).
```

```
## Warning: Removed 7 rows containing non-finite outside the scale range
## (`stat_boxplot()`).
```

```
## Warning: Removed 7 rows containing missing values or values outside the scale range
## (`geom_point()`).
```

```
## Warning: Removed 12 rows containing non-finite outside the scale range
## (`stat_ydensity()`).
```

```
## Warning: Removed 12 rows containing non-finite outside the scale range
## (`stat_boxplot()`).
```

```
## Warning: Removed 12 rows containing missing values or values outside the scale range
## (`geom_point()`).
```

```
## Warning: Removed 7 rows containing non-finite outside the scale range
## (`stat_ydensity()`).
```

```
## Warning: Removed 7 rows containing non-finite outside the scale range
## (`stat_boxplot()`).
```

```
## Warning: Removed 7 rows containing missing values or values outside the scale range
## (`geom_point()`).
```

```
## Warning: Removed 7 rows containing non-finite outside the scale range
## (`stat_ydensity()`).
```

```
## Warning: Removed 7 rows containing non-finite outside the scale range
## (`stat_boxplot()`).
```

```
## Warning: Removed 7 rows containing missing values or values outside the scale range
## (`geom_point()`).
```

```
## Warning: Removed 5 rows containing non-finite outside the scale range
## (`stat_ydensity()`).
```

```
## Warning: Removed 5 rows containing non-finite outside the scale range
## (`stat_boxplot()`).
```

```
## Warning: Removed 5 rows containing missing values or values outside the scale range
## (`geom_point()`).
```

```
## Warning: Removed 16 rows containing non-finite outside the scale range
## (`stat_ydensity()`).
```

```
## Warning: Removed 16 rows containing non-finite outside the scale range
## (`stat_boxplot()`).
```

```
## Warning: Removed 16 rows containing missing values or values outside the scale range
## (`geom_point()`).
```

```
## Warning: Removed 7 rows containing non-finite outside the scale range
## (`stat_ydensity()`).
```

```
## Warning: Removed 7 rows containing non-finite outside the scale range
## (`stat_boxplot()`).
```

```
## Warning: Removed 7 rows containing missing values or values outside the scale range
## (`geom_point()`).
```

```
## Warning: Removed 9 rows containing non-finite outside the scale range
## (`stat_ydensity()`).
```

```
## Warning: Removed 9 rows containing non-finite outside the scale range
## (`stat_boxplot()`).
```

```
## Warning: Removed 9 rows containing missing values or values outside the scale range
## (`geom_point()`).
```

Mixture modeling

```
mixtureModels <- data.frame(
  metric = character(),
  numberComponents = numeric(),
  BIC = numeric()
)

for (i in 1:length(colnames(Uintatheres))) {
  mixMod <- Mclust(Uintatheres[,i][!is.na(Uintatheres[,i])])
  assign(paste(colnames(Uintatheres)[i], "mixMod"), mixMod)
  mixtureModels[i,1] <- colnames(Uintatheres)[i]
  mixtureModels[i,2] <- mixMod$G
  mixtureModels[i,3] <- mixMod$bic
}
```

PART 2: Multivariate analysis

First, missing data must be imputed for PCA (Impute if 3 or fewer
missing, otherwise, remove specimen)

```
aggr(Uintatheres)
```

```
uinTrim <- Uintatheres[rowSums(is.na(Uintatheres)) < 5,]
aggr(uinTrim)
```

```
uinScale <- scale(uinTrim)


ut.ncp <- estim_ncpPCA(uinScale, method.cv= "Kfold")
```

```
##   |                                                                              |                                                                      |   0%  |                                                                              |=                                                                     |   1%  |                                                                              |=                                                                     |   2%  |                                                                              |==                                                                    |   3%  |                                                                              |===                                                                   |   4%  |                                                                              |====                                                                  |   5%  |                                                                              |====                                                                  |   6%  |                                                                              |=====                                                                 |   7%  |                                                                              |======                                                                |   8%  |                                                                              |======                                                                |   9%  |                                                                              |=======                                                               |  10%  |                                                                              |========                                                              |  11%  |                                                                              |========                                                              |  12%  |                                                                              |=========                                                             |  13%  |                                                                              |==========                                                            |  14%  |                                                                              |===========                                                           |  15%  |                                                                              |===========                                                           |  16%  |                                                                              |============                                                          |  17%  |                                                                              |=============                                                         |  18%  |                                                                              |=============                                                         |  19%  |                                                                              |==============                                                        |  20%  |                                                                              |===============                                                       |  21%  |                                                                              |================                                                      |  22%  |                                                                              |================                                                      |  23%  |                                                                              |=================                                                     |  24%  |                                                                              |==================                                                    |  25%  |                                                                              |==================                                                    |  26%  |                                                                              |===================                                                   |  27%  |                                                                              |====================                                                  |  28%  |                                                                              |=====================                                                 |  29%  |                                                                              |=====================                                                 |  30%  |                                                                              |======================                                                |  31%  |                                                                              |=======================                                               |  32%  |                                                                              |=======================                                               |  33%  |                                                                              |========================                                              |  34%  |                                                                              |=========================                                             |  35%  |                                                                              |=========================                                             |  36%  |                                                                              |==========================                                            |  37%  |                                                                              |===========================                                           |  38%  |                                                                              |============================                                          |  39%  |                                                                              |============================                                          |  40%  |                                                                              |=============================                                         |  41%  |                                                                              |==============================                                        |  42%  |                                                                              |==============================                                        |  43%  |                                                                              |===============================                                       |  44%  |                                                                              |================================                                      |  45%  |                                                                              |=================================                                     |  46%  |                                                                              |=================================                                     |  47%  |                                                                              |==================================                                    |  48%  |                                                                              |===================================                                   |  49%  |                                                                              |===================================                                   |  51%  |                                                                              |====================================                                  |  52%  |                                                                              |=====================================                                 |  53%  |                                                                              |=====================================                                 |  54%  |                                                                              |======================================                                |  55%  |                                                                              |=======================================                               |  56%  |                                                                              |========================================                              |  57%  |                                                                              |========================================                              |  58%  |                                                                              |=========================================                             |  59%  |                                                                              |==========================================                            |  60%  |                                                                              |==========================================                            |  61%  |                                                                              |===========================================                           |  62%  |                                                                              |============================================                          |  63%  |                                                                              |=============================================                         |  64%  |                                                                              |=============================================                         |  65%  |                                                                              |==============================================                        |  66%  |                                                                              |===============================================                       |  67%  |                                                                              |===============================================                       |  68%  |                                                                              |================================================                      |  69%  |                                                                              |=================================================                     |  70%  |                                                                              |=================================================                     |  71%  |                                                                              |==================================================                    |  72%  |                                                                              |===================================================                   |  73%  |                                                                              |====================================================                  |  74%  |                                                                              |====================================================                  |  75%  |                                                                              |=====================================================                 |  76%  |                                                                              |======================================================                |  77%  |                                                                              |======================================================                |  78%  |                                                                              |=======================================================               |  79%  |                                                                              |========================================================              |  80%  |                                                                              |=========================================================             |  81%  |                                                                              |=========================================================             |  82%  |                                                                              |==========================================================            |  83%  |                                                                              |===========================================================           |  84%  |                                                                              |===========================================================           |  85%  |                                                                              |============================================================          |  86%  |                                                                              |=============================================================         |  87%  |                                                                              |==============================================================        |  88%  |                                                                              |==============================================================        |  89%  |                                                                              |===============================================================       |  90%  |                                                                              |================================================================      |  91%  |                                                                              |================================================================      |  92%  |                                                                              |=================================================================     |  93%  |                                                                              |==================================================================    |  94%  |                                                                              |==================================================================    |  95%  |                                                                              |===================================================================   |  96%  |                                                                              |====================================================================  |  97%  |                                                                              |===================================================================== |  98%  |                                                                              |===================================================================== |  99%  |                                                                              |======================================================================| 100%
```

```
plot(ut.ncp$criterion~names(ut.ncp$criterion),xlab="number of dimensions", 
     ylab ="ncp criterion")
```

```
ncp <- as.numeric(ut.ncp$ncp[1]) 

uinImp <- imputePCA(uinScale, ncp = ncp)
uinImp <- uinImp$completeObs
uinImp <- as.data.frame(uinImp)
```

Hierarchical Clustering

```
uinSex <- uinImp
uinSex$sex <- NA

uinSex$sex <- ifelse(rownames(uinSex) %in% uinFem, "F", 
                     ifelse(rownames(uinSex) %in% uinMale, "M", "?"))

row_colors <- ifelse(uinSex$sex == "F", "red", 
                     ifelse(uinSex$sex == "M", "blue", "black"))

Heatmap(uinImp,
        name = "Z-score",
        cluster_rows = TRUE,
        cluster_columns = TRUE,
        clustering_distance_rows = "euclidean",
        clustering_distance_columns = "euclidean",
        clustering_method_rows = "complete",
        clustering_method_columns = "complete",
        column_names_rot = 65,
        column_names_gp = gpar(fontsize = 10),
        row_names_gp = gpar(col = row_colors, fontsize = 15),
        row_dend_width = unit(75, "points")
)
```

```
## Warning: The input is a data frame-like object, convert it to a matrix.
```

```
###ALTERNATIVE CLUSTERING WITH BOOTSTRAP SUPPORT VALUES

bootMap <- pvclust(t(uinImp), method.dist="euclidean", method.hclust="complete", nboot=1000)
```

```
## Bootstrap (r = 0.44)... Done.
## Bootstrap (r = 0.56)... Done.
## Bootstrap (r = 0.67)... Done.
## Bootstrap (r = 0.78)... Done.
## Bootstrap (r = 0.89)... Done.
## Bootstrap (r = 1.0)... Done.
## Bootstrap (r = 1.11)... Done.
## Bootstrap (r = 1.22)... Done.
## Bootstrap (r = 1.33)... Done.
```

```
plot(bootMap)
pvrect(bootMap, alpha=0.95)
```

```
par(mar = c(4.5, 2, 2, 2))
plot(bootMap, labels = FALSE, hang = -5, main = "Hierarchical Clustering of Uintathere Skulls", print.num = F,
     xlab = "", sub="")
tipLabels <- bootMap$hclust$labels[bootMap$hclust$order]
tipColors <- ifelse(bootMap$hclust$labels[bootMap$hclust$order] %in% uinFem, "red",
                    ifelse(bootMap$hclust$labels[bootMap$hclust$order] %in% uinMale, "blue", "black"))
text(x = 1:length(tipLabels), y = -0.02, labels = tipLabels, col = tipColors,
     srt = 90,adj = 1,xpd = TRUE, cex = 0.75, font = 2)
```

```
dev.off()
```

```
## null device 
##           1
```

```
uinImp$sex <- uinSex$sex
uinImp$sex <- as.factor(uinImp$sex)

###MANOVA AND R2 FOR HEATMAP

uinHeatMan <- manova(cbind(nasOcc, nasCon, preCon, occCon, parPro, maxAlv, 
                           alvCan, diaLen, canWid) ~ sex, data = uinImp)
summary(uinHeatMan, test = "Pillai")
```

```
##           Df Pillai approx F num Df den Df  Pr(>F)  
## sex        2 1.4108   2.1285     18     16 0.06755 .
## Residuals 15                                        
## ---
## Signif. codes:  0 '***' 0.001 '**' 0.01 '*' 0.05 '.' 0.1 ' ' 1
```

```
summary.aov(uinHeatMan)
```

```
##  Response nasOcc :
##             Df Sum Sq Mean Sq F value  Pr(>F)  
## sex          2 7.6831  3.8416  6.1849 0.01099 *
## Residuals   15 9.3169  0.6211                  
## ---
## Signif. codes:  0 '***' 0.001 '**' 0.01 '*' 0.05 '.' 0.1 ' ' 1
## 
##  Response nasCon :
##             Df  Sum Sq Mean Sq F value Pr(>F)
## sex          2  0.2262 0.11308  0.1075 0.8987
## Residuals   15 15.7739 1.05159               
## 
##  Response preCon :
##             Df  Sum Sq Mean Sq F value Pr(>F)
## sex          2  1.2347 0.61733  0.7732 0.4791
## Residuals   15 11.9759 0.79840               
## 
##  Response occCon :
##             Df  Sum Sq Mean Sq F value Pr(>F)
## sex          2  1.9878 0.99390   1.064 0.3697
## Residuals   15 14.0123 0.93415               
## 
##  Response parPro :
##             Df Sum Sq Mean Sq F value   Pr(>F)   
## sex          2 7.5973  3.7986  6.5568 0.008991 **
## Residuals   15 8.6901  0.5793                    
## ---
## Signif. codes:  0 '***' 0.001 '**' 0.01 '*' 0.05 '.' 0.1 ' ' 1
## 
##  Response maxAlv :
##             Df  Sum Sq Mean Sq F value  Pr(>F)  
## sex          2  6.0612 3.03058  4.1557 0.03664 *
## Residuals   15 10.9388 0.72926                  
## ---
## Signif. codes:  0 '***' 0.001 '**' 0.01 '*' 0.05 '.' 0.1 ' ' 1
## 
##  Response alvCan :
##             Df Sum Sq Mean Sq F value  Pr(>F)  
## sex          2 3.7716  1.8858  5.0008 0.02167 *
## Residuals   15 5.6565  0.3771                  
## ---
## Signif. codes:  0 '***' 0.001 '**' 0.01 '*' 0.05 '.' 0.1 ' ' 1
## 
##  Response diaLen :
##             Df  Sum Sq Mean Sq F value Pr(>F)
## sex          2  1.9756 0.98782  1.0452 0.3759
## Residuals   15 14.1759 0.94506               
## 
##  Response canWid :
##             Df  Sum Sq Mean Sq F value Pr(>F)
## sex          2  3.5654 1.78268  2.3666 0.1278
## Residuals   15 11.2989 0.75326
```

```
eta_squared(uinHeatMan, partial = T)
```

```
## # Effect Size for ANOVA (Type I)
## 
## Parameter | Eta2 (partial) |       95% CI
## -----------------------------------------
## sex       |           0.71 | [0.00, 1.00]
## 
## - One-sided CIs: upper bound fixed at [1.00].
```

PCA

```
uinImpPCA <- PCA(uinImp[,1:9], scale.unit=F)
```

```
# write.csv(uinImpPCA$eig, "eigVals.csv")
# write.csv(uinImpPCA$var$coord, "pcLoad.csv")
# write.csv(uinImpPCA$var$cos2, "pcContVar.csv")
# write.csv(uinImpPCA$var$contrib, "varContPc.csv")

fviz_pca_var(uinImpPCA, col.var = "contrib")
```

```
fviz_pca_ind(uinImpPCA)
```

```
fviz_pca_biplot(uinImpPCA)
```

```
#Designate purported males and females to be separated into different hulls in PCA

coords <- uinImpPCA$ind$coord
coords <- as.data.frame(coords)
isFem <- rownames(coords) %in% uinFem
coords$Fem <- isFem
coords$sex <- uinSex$sex
coords$sex <- as.factor(coords$sex)

hull_sex <- coords %>%
  group_by(sex) %>%
  slice(chull(Dim.1, Dim.2))

hull_sex$sex <- as.factor(hull_sex$sex)
hull_sex$Sex <- hull_sex$sex

coords$Sex <- as.factor(coords$sex)

MyPlot <- ggplot(coords, aes(Dim.1, Dim.2)) + geom_point(shape = 21)
MyPlot + aes(fill = factor(sex)) + geom_polygon(data = hull_sex, alpha = 0.5) +
  labs(x = "PC1", y = "PC2", title = "PCA of Uintatherium Skulls")+ geom_vline(xintercept = 0) + geom_hline(yintercept = 0) +
  theme(legend.title = element_blank()) +
  geom_label_repel(aes(label = rownames(coords))) +
  scale_fill_manual(values = c("#B8B8B8", "#F8766D", "#619CFF"))
```

```
MyPlot +
  geom_polygon(data = hull_sex, aes(group = sex, fill = sex), alpha = 0.5) +
  labs(x = "PC1", y = "PC2", title = "PCA of Uintatherium Skulls") +
  geom_vline(xintercept = 0) +
  geom_hline(yintercept = 0) +
  geom_label_repel(aes(label = rownames(coords))) +
  scale_fill_manual(
    name = "Sex",  # legend title
    values = c(
      "F" = "#F8766D",
      "M" = "#619CFF",
      "?" = "#B8B8B8"
    )
  )
```

Determine number of clusters data appears to form

```
gapStat <- fviz_nbclust(uinImp[,1:9], kmeans, k.max = 8, method = "gap_stat")
fviz_nbclust(uinImp[,1:9], kmeans, k.max = 8, method = "gap_stat")
```

```
gapStat <- fviz_nbclust(uinImp[,1:9], kmeans, k.max = 17, method = "gap_stat")
wss <- fviz_nbclust(uinImp[,1:9], kmeans, k.max = 17, method = "wss")
silh <- fviz_nbclust(uinImp[,1:9], kmeans, k.max = 17, method = "silhouette")

wss / silh / gapStat
```

Use k-means clustering to form two most favorable clusters

```
km.UinTrimImp <- kmeans(uinImp[,1:9], centers = 2)
UintatheresClustered <- uinImp
clustVec <- km.UinTrimImp$cluster
UintatheresClustered <- as.data.frame(UintatheresClustered)
UintatheresClustered$cluster = clustVec
UintatheresClustered$cluster <- as.factor(UintatheresClustered$cluster)

ClusteredPCA <- PCA(UintatheresClustered[,1:9])
```

```
clusterCoords <-ClusteredPCA$ind$coord
clusterCoords <- as.data.frame(coords)
clusterCoords <- clusterCoords[,-c(6,7)]
clusterCoords$Cluster <- UintatheresClustered$cluster

hull_cluster <- clusterCoords %>%
  group_by(Cluster) %>%
  slice(chull(Dim.1, Dim.2))

MyClusterPlot <- ggplot(clusterCoords, aes(x = Dim.1, y = Dim.2)) + geom_point(shape = 21)

MyClusterPlot + aes(fill = Cluster) + geom_polygon(data = hull_cluster, alpha = 0.5) +
  labs(x = "PC1", y = "PC2", colour = "Cluster", title = "PCA of Uintatherium Skulls") + 
  geom_vline(xintercept = 0) + geom_hline(yintercept = 0) +
  geom_label_repel(aes(label = rownames(clusterCoords))) +
  scale_fill_manual(values = c("#619CFF", "#F8766D"))
```

```
## Ignoring unknown labels:
## • colour : "Cluster"
```

```
hull_sex$sex <- as.factor(hull_sex$sex)
hull_sex$Sex <- hull_sex$sex
```

Plot purported sex and cluster on the same PCA

```
MyClusterPlot +
  geom_polygon(
    data = hull_cluster,
    aes(x = Dim.1, y = Dim.2, group = Cluster, fill = factor(Cluster)),
    alpha = 0.25,
    linewidth = 0.3,
    colour = "gray25",
    inherit.aes = FALSE) +
  scale_fill_manual(name = "Cluster", values = c("1" = "#4DB6AC", "2" = "#D4A72C")) +
  new_scale_fill() +
  geom_point(aes(fill = Sex), shape = 21, size = 3) +
  geom_label_repel(aes(label = rownames(clusterCoords), fill = Sex),colour = "black" ) +
  scale_fill_manual(values = c("?" = "#B8B8B8","F" = "#F8766D","M" = "#619CFF")) +
  labs(x = "PC1 (60.05%)", y = "PC2 (19.61%)", title = "PCA of Uintathere Skulls") +
  geom_vline(xintercept = 0) +
  geom_hline(yintercept = 0)
```

Perform MANOVA to see if significant PC scores are predicted by
purported sex

```
sigCols <- uinImpPCA$ind$coord[,1:4]
sigCols <- as.data.frame(sigCols)
sigCols$sex <- NA
sigCols$sex <- ifelse(rownames(sigCols) %in% uinFem, "F", 
                      ifelse(rownames(sigCols) %in% uinMale, "M", "?"))
uinPCAMan <- manova(cbind(Dim.1, Dim.2, Dim.3, Dim.4) ~ sex, data = sigCols)
summary(uinPCAMan)
```

```
##           Df Pillai approx F num Df den Df   Pr(>F)   
## sex        2 1.0229   3.4024      8     26 0.008338 **
## Residuals 15                                          
## ---
## Signif. codes:  0 '***' 0.001 '**' 0.01 '*' 0.05 '.' 0.1 ' ' 1
```

```
eta_squared(uinPCAMan, partial = TRUE)
```

```
## # Effect Size for ANOVA (Type I)
## 
## Parameter | Eta2 (partial) |       95% CI
## -----------------------------------------
## sex       |           0.51 | [0.13, 1.00]
## 
## - One-sided CIs: upper bound fixed at [1.00].
```

Lastly, estimate the degree of dimorphism in each metric using finite
mixture analysis and the method of means

```
estimateTable <- matrix(nrow = 9, ncol = 2)
rownames(estimateTable) <- colnames(Uintatheres)
colnames(estimateTable) <- c("FMA", "MM")
estimateTable <- as.data.frame(estimateTable)

pearsonTable <- read.csv("pearsonTable.csv")

for (i in 1:nrow(estimateTable)) {
  metric <- na.omit(Uintatheres[,i])
  mean <- mean(metric)
  OR <- max(metric) - min(metric)
  n <- length(metric)
  k <- pearsonTable$stDevsInRange[pearsonTable$SampleSize == n]
  sigmaTot <- OR/k
  expSub <- k*(2^.5) #expected number of subpop st devs in total OR
  twoSubPercent <- 2/expSub #portion of OR expected to be occupied by 2 sigmaSub
  oneSubPercent <- twoSubPercent/2 #portion of OR expected to be occupied by 1 sigmaSub
  distanceToSub <- oneSubPercent*OR #numeric value of one sigmaSub <- distance from overall mean to mean of each subpop
  bigMean <- mean + distanceToSub
  smallMean <- mean - distanceToSub
  fmaEstimate <- bigMean/smallMean
  estimateTable$FMA[i] <- fmaEstimate
  bigs <- metric[metric > mean(metric)]
  bigMean <- mean(bigs)
  smalls <- metric[metric < mean(metric)]
  smallMean <- mean(smalls)
  mmEstimate <- bigMean/smallMean
  estimateTable$MM[i] <- mmEstimate
}
```

Same with bison now Load and manipulate data

```
bison <- read.csv("Bison_Measurements.csv", check.names = F) #load dataframe
bisonAdults <- bison[-20, ] #remove 68350, specimen with adult dentition still erupting
rownames(bisonAdults) <- bisonAdults[,1] #rename rows as specimen numbers
adultSex <- bisonAdults[,2] #make a vector of sex for adult specimens
bisonAdults <- bisonAdults[,-c(1:4)] #remove helper variables
for (i in 1:ncol(bisonAdults)) {
  bisonAdults[,i] <- as.numeric(bisonAdults[,i])
}

str(bisonAdults)
```

```
## 'data.frame':    23 obs. of  9 variables:
##  $ tipFro: num  41.1 NA 43.9 47.8 NA ...
##  $ basFro: num  19.8 26.5 22.1 20.2 18.8 ...
##  $ ectEct: num  24.6 29.5 27.4 24.1 23.6 ...
##  $ maxMax: num  13.3 16.5 14.4 14.2 12.4 ...
##  $ proNas: num  23 27.8 25.1 24.4 23.5 ...
##  $ nasNuc: num  16.2 19.1 19 19.4 16 ...
##  $ basTip: num  12.9 20.5 12.9 16.5 NA ...
##  $ ectPro: num  29.8 34.7 31.8 32.9 29.7 ...
##  $ basWid: num  4.34 7.14 5.07 5.17 4.3 ...
```

```
bisonAdults <- bisonAdults[,-4] #remove maxMax to retain 8 variables

bisonSex <- bisonAdults
bisonSex$sex <- adultSex

set.seed(123)

bisonAdults <- bisonAdults * runif(length(unlist(bisonAdults)), 0.865, 1.135)
```

PART 1: Generate histograms for each variable and test for normality
and unimodality

Setting up “Stats” dataframe to store values of various test
statistics of each measurement

```
columns <- c("Width between tips of frontal Horns", "Width bewteen bases of Frontal Horns", 
             "Width between exorbitale",
             "Length from prosthion to nasion", "Length from nasion to nuchal crest", 
             "Length of frontal horn from base to tip", "Length of skull from orbit to prosthion", 
             "Width of frontal horn at base")
NVariables <- length(columns)
Measurement <- vector()
n <- vector()
wStatistic <- vector()
pValue <- vector()
dipStatistic <- vector() 

#The 'width  between tips of frontal horns' measurement is not considered in final analyses

Stats <- data.frame(Measurement, n, wStatistic, pValue, dipStatistic, pValue)
```

RUN for-loop that plots histograms for each variable, runs
Shapiro-Wilk and Hartigan’s Dip Test, and stores these variables in
“Stats” dataframe.

```
par(mfrow = c(4,2))
par(mar=c(2,2,2,2))

for (i in 1:NVariables) {
  histi <- hist(bisonAdults[,i][!is.na(bisonAdults[,i])], main = paste(columns[i]),
                col = "skyblue1", xlab = paste(columns[i], "(cm)"))
  lengthi <- unique(bisonAdults[,i][!is.na(bisonAdults[,i])])
  xfit <- seq(min(bisonAdults[,i][!is.na(bisonAdults[,i])], na.rm = T), 
              max(bisonAdults[,i][!is.na(bisonAdults[,i])], na.rm = T), 
              length = length(lengthi)) 
  yfit <- dnorm(xfit, mean = mean(bisonAdults[,i][!is.na(bisonAdults[,i])], na.rm = T), 
                sd = sd(bisonAdults[,i][!is.na(bisonAdults[,i])], na.rm = T)) 
  yfit <- yfit * diff(histi$mids[1:2]) * length(bisonAdults[,i][!is.na(bisonAdults[,i])]) 
  lines(xfit, yfit, col = "red", lwd = 3)
  Stats[i,1] <- columns[i]
  Stats[i,2] <- length(bisonAdults[,i][!is.na(bisonAdults[,i])])
  Stats[i,3] <- shapiro.test(bisonAdults[,i])[1]
  Stats[i,4] <- shapiro.test(bisonAdults[,i])[2]
  Stats[i,5] <- dip.test(bisonAdults[,i])[1]
  Stats[i,6] <- dip.test(bisonAdults[,i])[2]
  mtext(paste("w-statistic: ", Stats[i,3], "; p-value: ", Stats[i,4], "\n",
              "dip-statistic: ", Stats[i,5], "; p-value: ", Stats[i,6],  sep = ""), 
        padj = .3, side = 4, cex = 0.45)
}
```

```
par(mfrow = c(1,1))
```

Generate Violin Plots for Each Measurement

```
par(mfrow = c(4,2))
par(mar=c(2,2,2,2))

dims <- list(length(colnames(bisonAdults)))
dims[[8]] <- ggplot(data = bisonAdults,
                  mapping = aes(x="", y= 'width between tips of frontal Horns' )) +
  geom_violin( fill = "gray80", trim = F) + geom_point() + geom_boxplot(width = 0.1, fill = "gray70") +
  labs(y = "Length (cm)", x="", title = "Width between tips of frontal Horns")


for (i in 1:length(colnames(bisonAdults))) {
  dims[[i]] <-  ggplot(data = bisonAdults,
                       mapping = aes(x="", y=.data[[colnames(bisonAdults)[i]]])) +
    geom_violin( fill = "gray80", trim = F) + geom_point() + geom_boxplot(width = 0.1, fill = "gray70") +
    labs(y = "Length (cm)", x="", title = colnames(bisonAdults)[i])
}

grid.arrange(dims[[1]], dims[[2]], dims[[3]], dims[[4]], dims[[5]], dims[[6]], dims[[7]], dims[[8]],
             ncol = 3, nrow = 3)
```

```
## Warning: Removed 12 rows containing non-finite outside the scale range
## (`stat_ydensity()`).
```

```
## Warning: Removed 12 rows containing non-finite outside the scale range
## (`stat_boxplot()`).
```

```
## Warning: Removed 12 rows containing missing values or values outside the scale range
## (`geom_point()`).
```

```
## Warning: Removed 3 rows containing non-finite outside the scale range
## (`stat_ydensity()`).
```

```
## Warning: Removed 3 rows containing non-finite outside the scale range
## (`stat_boxplot()`).
```

```
## Warning: Removed 3 rows containing missing values or values outside the scale range
## (`geom_point()`).
```

```
## Warning: Removed 5 rows containing non-finite outside the scale range
## (`stat_ydensity()`).
```

```
## Warning: Removed 5 rows containing non-finite outside the scale range
## (`stat_boxplot()`).
```

```
## Warning: Removed 5 rows containing missing values or values outside the scale range
## (`geom_point()`).
```

```
## Warning: Removed 3 rows containing non-finite outside the scale range
## (`stat_ydensity()`).
```

```
## Warning: Removed 3 rows containing non-finite outside the scale range
## (`stat_boxplot()`).
```

```
## Warning: Removed 3 rows containing missing values or values outside the scale range
## (`geom_point()`).
```

Generate random normal data for reference

```
set.seed(123)

MyMin <- min(bisonAdults$tipFro, na.rm = T)
MyMax <- max(bisonAdults$tipFro, na.rm = T)
MyMean <- mean(bisonAdults$tipFro, na.rm = T)
MyVar <- var(bisonAdults$tipFro, na.rm = T)
MySD <- sqrt(MyVar)
MyNorm <- rnorm(22, mean = MyMean, sd = MySD)
MyData <- data.frame(random = 1:22)
MyData$random <- MyNorm

random <- ggplot(data = MyData,
       mapping = aes(x="", y= `random` )) +
  geom_violin( fill = "gray80", trim = F) + geom_point() + geom_boxplot(width = 0.1, fill = "gray70") +
  labs(y = "Length (cm)", x="", title = "Random Normal Data")

#plot with random normal data

grid.arrange(dims[[2]], dims[[3]], dims[[4]], dims[[5]], dims[[6]], dims[[7]], dims[[8]], random,
             ncol = 2, nrow = 4)
```

```
## Warning: Removed 3 rows containing non-finite outside the scale range
## (`stat_ydensity()`).
```

```
## Warning: Removed 3 rows containing non-finite outside the scale range
## (`stat_boxplot()`).
```

```
## Warning: Removed 3 rows containing missing values or values outside the scale range
## (`geom_point()`).
```

```
## Warning: Removed 5 rows containing non-finite outside the scale range
## (`stat_ydensity()`).
```

```
## Warning: Removed 5 rows containing non-finite outside the scale range
## (`stat_boxplot()`).
```

```
## Warning: Removed 5 rows containing missing values or values outside the scale range
## (`geom_point()`).
```

```
## Warning: Removed 3 rows containing non-finite outside the scale range
## (`stat_ydensity()`).
```

```
## Warning: Removed 3 rows containing non-finite outside the scale range
## (`stat_boxplot()`).
```

```
## Warning: Removed 3 rows containing missing values or values outside the scale range
## (`geom_point()`).
```

Mixture modeling

```
mixtureModels <- data.frame(
  metric = character(),
  numberComponents = numeric(),
  BIC = numeric()
)

for (i in 1:length(colnames(bisonAdults))) {
  mixMod <- Mclust(bisonAdults[,i][!is.na(bisonAdults[,i])])
  assign(paste(colnames(bisonAdults)[i], "mixMod"), mixMod)
  mixtureModels[i,1] <- colnames(bisonAdults)[i]
  mixtureModels[i,2] <- mixMod$G
  mixtureModels[i,3] <- mixMod$bic
}

par(mfrow=c(1,1))
```

PART 2: Multivariate analysis

First, missing data must be imputed for PCA (Impute if 3 or fewer
missing, otherwise, remove specimen)

```
bisonAdults <- bisonAdults[,-1] #remove extra horn variable

bis.ncp <- estim_ncpPCA(bisonAdults, method.cv= "Kfold")
```

```
##   |                                                                              |                                                                      |   0%  |                                                                              |=                                                                     |   1%  |                                                                              |=                                                                     |   2%  |                                                                              |==                                                                    |   3%  |                                                                              |===                                                                   |   4%  |                                                                              |====                                                                  |   5%  |                                                                              |====                                                                  |   6%  |                                                                              |=====                                                                 |   7%  |                                                                              |======                                                                |   8%  |                                                                              |======                                                                |   9%  |                                                                              |=======                                                               |  10%  |                                                                              |========                                                              |  11%  |                                                                              |========                                                              |  12%  |                                                                              |=========                                                             |  13%  |                                                                              |==========                                                            |  14%  |                                                                              |===========                                                           |  15%  |                                                                              |===========                                                           |  16%  |                                                                              |============                                                          |  17%  |                                                                              |=============                                                         |  18%  |                                                                              |=============                                                         |  19%  |                                                                              |==============                                                        |  20%  |                                                                              |===============                                                       |  21%  |                                                                              |================                                                      |  22%  |                                                                              |================                                                      |  23%  |                                                                              |=================                                                     |  24%  |                                                                              |==================                                                    |  25%  |                                                                              |==================                                                    |  26%  |                                                                              |===================                                                   |  27%  |                                                                              |====================                                                  |  28%  |                                                                              |=====================                                                 |  29%  |                                                                              |=====================                                                 |  30%  |                                                                              |======================                                                |  31%  |                                                                              |=======================                                               |  32%  |                                                                              |=======================                                               |  33%  |                                                                              |========================                                              |  34%  |                                                                              |=========================                                             |  35%  |                                                                              |=========================                                             |  36%  |                                                                              |==========================                                            |  37%  |                                                                              |===========================                                           |  38%  |                                                                              |============================                                          |  39%  |                                                                              |============================                                          |  40%  |                                                                              |=============================                                         |  41%  |                                                                              |==============================                                        |  42%  |                                                                              |==============================                                        |  43%  |                                                                              |===============================                                       |  44%  |                                                                              |================================                                      |  45%  |                                                                              |=================================                                     |  46%  |                                                                              |=================================                                     |  47%  |                                                                              |==================================                                    |  48%  |                                                                              |===================================                                   |  49%  |                                                                              |===================================                                   |  51%  |                                                                              |====================================                                  |  52%  |                                                                              |=====================================                                 |  53%  |                                                                              |=====================================                                 |  54%  |                                                                              |======================================                                |  55%  |                                                                              |=======================================                               |  56%  |                                                                              |========================================                              |  57%  |                                                                              |========================================                              |  58%  |                                                                              |=========================================                             |  59%  |                                                                              |==========================================                            |  60%  |                                                                              |==========================================                            |  61%  |                                                                              |===========================================                           |  62%  |                                                                              |============================================                          |  63%  |                                                                              |=============================================                         |  64%  |                                                                              |=============================================                         |  65%  |                                                                              |==============================================                        |  66%  |                                                                              |===============================================                       |  67%  |                                                                              |===============================================                       |  68%  |                                                                              |================================================                      |  69%  |                                                                              |=================================================                     |  70%  |                                                                              |=================================================                     |  71%  |                                                                              |==================================================                    |  72%  |                                                                              |===================================================                   |  73%  |                                                                              |====================================================                  |  74%  |                                                                              |====================================================                  |  75%  |                                                                              |=====================================================                 |  76%  |                                                                              |======================================================                |  77%  |                                                                              |======================================================                |  78%  |                                                                              |=======================================================               |  79%  |                                                                              |========================================================              |  80%  |                                                                              |=========================================================             |  81%  |                                                                              |=========================================================             |  82%  |                                                                              |==========================================================            |  83%  |                                                                              |===========================================================           |  84%  |                                                                              |===========================================================           |  85%  |                                                                              |============================================================          |  86%  |                                                                              |=============================================================         |  87%  |                                                                              |==============================================================        |  88%  |                                                                              |==============================================================        |  89%  |                                                                              |===============================================================       |  90%  |                                                                              |================================================================      |  91%  |                                                                              |================================================================      |  92%  |                                                                              |=================================================================     |  93%  |                                                                              |==================================================================    |  94%  |                                                                              |==================================================================    |  95%  |                                                                              |===================================================================   |  96%  |                                                                              |====================================================================  |  97%  |                                                                              |===================================================================== |  98%  |                                                                              |===================================================================== |  99%  |                                                                              |======================================================================| 100%
```

```
plot(bis.ncp$criterion~names(bis.ncp$criterion),xlab="number of dimensions", 
     ylab = "ncp criterion")
```

```
ncp <- as.numeric(bis.ncp$ncp[1]) #The optimal number of dimensions(components) which should considered when imputing data, as obtained by k-fold cross validation

bis.impute <- imputePCA(bisonAdults, ncp = ncp)
bis.impute <- bis.impute$completeObs
bis.impute <- as.data.frame(bis.impute)

bis.impute #Missing values are now imputed according to the PCA cross-validation.
```

```
##          basFro   ectEct   proNas   nasNuc   basTip   ectPro   basWid
## 21904  22.43759 22.81333 22.62433 15.50454 11.64457 33.71468 4.171087
## 16211  27.62145 29.25824 29.73194 19.89689 20.78792 34.08614 7.423984
## 21903  23.36142 25.64330 26.01449 18.05434 14.45573 30.21712 4.902527
## 141977 20.41000 26.46815 25.83637 17.74121 16.83001 32.13666 4.966396
## 141976 19.26398 20.73195 20.35229 17.20983 12.06505 25.80386 4.341897
## 92698  25.28390 29.82160 28.53461 20.10693 19.48144 33.64320 8.945973
## 2504   16.94349 25.20527 22.69236 15.79836 12.96757 33.87510 4.163826
## 2499   24.91459 21.50215 24.79090 17.06392 13.39562 30.54836 4.651769
## 2500   22.08737 23.55228 24.82751 17.41564 13.93133 28.54386 4.428249
## 2501   19.92682 21.98398 25.24106 14.52519 13.57709 28.55643 4.938906
## 2495   25.81192 22.95906 23.46955 17.43205 20.41288 30.80221 4.683121
## 140290 25.11506 34.72821 30.24401 25.05206 21.67793 34.89809 8.468720
## 2414   27.71293 29.47231 30.40836 17.56995 16.55479 38.05760 6.810346
## 2494   22.66277 24.26949 26.29312 18.22266 16.18873 33.53726 4.712936
## 21905  22.48600 26.93349 27.45646 21.07362 16.88696 31.40612 4.719242
## 75033  24.95000 28.87220 26.61374 20.56141 21.75438 33.37557 8.352380
## 117340 24.73054 29.90500 23.22999 22.18487 23.53600 28.74650 7.805108
## 21906  23.90608 30.02102 31.17274 19.33279 22.29509 33.30483 9.183687
## 48115  23.87887 31.50738 28.96853 18.14200 19.42218 33.87057 7.930531
## 73940  20.07198 24.60295 26.02564 16.43452 14.60303 28.25048 5.136008
## 2498   19.64965 25.56900 23.72314 13.39508 14.02587 31.63173 3.842569
## 2497   19.43145 26.69003 22.35749 19.36641 12.68220 29.18763 4.269539
## 2022   24.44407 32.24877 28.21718 23.85531 17.10858 33.41017 6.346077
```

Hierarchical Clustering

```
bis.impute$sex <- adultSex

bis.impute$sex <- as.factor(bis.impute$sex)

bisNum <- bis.impute[,1:7]

bisScale <- scale(bisNum) #need to scale data before running PCA

bisScale <- as.data.frame(bisScale)

row_colors <- ifelse(adultSex == "F", "red", 
                     ifelse(adultSex == "M", "blue", "black"))
Heatmap(bisScale,
        name = "Z-score",
        cluster_rows = TRUE,
        cluster_columns = TRUE,
        column_names_rot = 65,
        column_names_gp = gpar(fontsize = 10),
        row_names_gp = gpar(col = row_colors, fontsize = 15),
        row_dend_width = unit(100, "points"))  # <- color the labels
```

```
## Warning: The input is a data frame-like object, convert it to a matrix.
```

```
###ALTERNATIVE CLUSTERING WITH BOOTSTRAP SUPPORT VALUES

bootMap <- pvclust(t(bisScale), method.dist="euclidean", method.hclust="complete", nboot=1000)
```

```
## Bootstrap (r = 0.43)... Done.
## Bootstrap (r = 0.57)... Done.
## Bootstrap (r = 0.71)... Done.
## Bootstrap (r = 0.86)... Done.
## Bootstrap (r = 1.0)... Done.
## Bootstrap (r = 1.14)... Done.
## Bootstrap (r = 1.29)... Done.
```

```
par(mar=c(1.25,2,2,2))
plot(bootMap,labels = FALSE, hang = -1, main = "Hierarchical Clustering of Bison Skulls", print.num = F)
tipLabels <- bootMap$hclust$labels[bootMap$hclust$order]
bisFem <- rownames(bisonSex)[bisonSex$sex=="F"]
bisMale <- rownames(bisonSex)[bisonSex$sex=="M"]
bisNB <- rownames(bisonSex)[bisonSex$sex=="?"]
tipColors <- ifelse(bootMap$hclust$labels[bootMap$hclust$order] %in% bisFem, "red",
                    ifelse(bootMap$hclust$labels[bootMap$hclust$order] %in% bisMale, "blue", "black"))
text(x = 1:length(tipLabels), y = -0.02, labels = tipLabels, col = tipColors,
  srt = 90,adj = 1,xpd = TRUE,cex = 0.75)
```

```
###MANOVA AND R2 FOR HEATMAP
bisScale$sex <- bisonSex$sex

bisHeatMan <- manova(cbind(basFro, ectEct, proNas, nasNuc, basTip, ectPro, basWid) ~ sex, data = bisScale)
summary(bisHeatMan)
```

```
##           Df  Pillai approx F num Df den Df Pr(>F)
## sex        2 0.85432   1.5979     14     30 0.1373
## Residuals 20
```

```
summary.aov(bisHeatMan)
```

```
##  Response basFro :
##             Df  Sum Sq Mean Sq F value Pr(>F)
## sex          2  4.5115 2.25575  2.5797 0.1008
## Residuals   20 17.4885 0.87443               
## 
##  Response ectEct :
##             Df  Sum Sq Mean Sq F value   Pr(>F)   
## sex          2  9.4307  4.7153  7.5029 0.003706 **
## Residuals   20 12.5693  0.6285                    
## ---
## Signif. codes:  0 '***' 0.001 '**' 0.01 '*' 0.05 '.' 0.1 ' ' 1
## 
##  Response proNas :
##             Df Sum Sq Mean Sq F value   Pr(>F)   
## sex          2 10.256  5.1279  8.7328 0.001879 **
## Residuals   20 11.744  0.5872                    
## ---
## Signif. codes:  0 '***' 0.001 '**' 0.01 '*' 0.05 '.' 0.1 ' ' 1
## 
##  Response nasNuc :
##             Df Sum Sq Mean Sq F value Pr(>F)
## sex          2  1.326 0.66301  0.6414 0.5371
## Residuals   20 20.674 1.03370               
## 
##  Response basTip :
##             Df  Sum Sq Mean Sq F value  Pr(>F)  
## sex          2  5.1734 2.58671  3.0745 0.06851 .
## Residuals   20 16.8266 0.84133                  
## ---
## Signif. codes:  0 '***' 0.001 '**' 0.01 '*' 0.05 '.' 0.1 ' ' 1
## 
##  Response ectPro :
##             Df  Sum Sq Mean Sq F value Pr(>F)  
## sex          2  7.5442  3.7721  5.2188  0.015 *
## Residuals   20 14.4558  0.7228                 
## ---
## Signif. codes:  0 '***' 0.001 '**' 0.01 '*' 0.05 '.' 0.1 ' ' 1
## 
##  Response basWid :
##             Df Sum Sq Mean Sq F value   Pr(>F)   
## sex          2 10.209  5.1043  8.6576 0.001956 **
## Residuals   20 11.791  0.5896                    
## ---
## Signif. codes:  0 '***' 0.001 '**' 0.01 '*' 0.05 '.' 0.1 ' ' 1
```

```
eta_squared(bisHeatMan, partial = TRUE)
```

```
## # Effect Size for ANOVA (Type I)
## 
## Parameter | Eta2 (partial) |       95% CI
## -----------------------------------------
## sex       |           0.43 | [0.00, 1.00]
## 
## - One-sided CIs: upper bound fixed at [1.00].
```

PCA

```
normPCA <- PCA(bisScale[,1:7])
```

```
#write.csv(normPCA$eig, "newBisonEigVals.csv")
#write.csv(normPCA$var$coord, "neBisonPCLoad.csv")
# write.csv(normPCA$var$cos2, "bisonPCContVar.csv")
# write.csv(normPCA$var$contrib, "bisonVarContPc.csv")
# 
fviz_pca_ind(normPCA)
```

```
fviz_pca_biplot(normPCA)
```

```
coords <- normPCA$ind$coord
coords <- as.data.frame(coords)
bisonSex <- bisonAdults
bisonSex$sex <- adultSex
isFem <- bisonSex$sex=="F"
coords$Fem <- isFem
coords$sex <- adultSex
coords$sex <- as.factor(coords$sex)

hull_sex <- coords %>%
  group_by(sex) %>%
  slice(chull(Dim.1, Dim.2))

coords$Sex <- as.factor(coords$sex)

MyPlot <- ggplot(coords, aes(Dim.1, Dim.2)) + geom_point(shape = 21)
MyPlot + aes(fill = factor(sex)) + geom_polygon(data = hull_sex, alpha = 0.5) +
  labs(x = "PC1", y = "PC2", title = "PCA of Bison Skulls")+ geom_vline(xintercept = 0) + geom_hline(yintercept = 0) +
  theme(legend.title = element_blank()) +
  geom_label_repel(aes(label = rownames(coords))) +
  scale_fill_manual(values = c("#B8B8B8", "#F8766D", "#619CFF"))
```

```
# write.csv(normPCA$eig, "eigVals.csv")
# write.csv(normPCA$var$coord, "pcLoad.csv")
# write.csv(normPCA$var$cos2, "pcContVar.csv")
# write.csv(normPCA$var$contrib, "varContPc.csv")
```

Determine number of clusters data appears to form

```
gapStat <- fviz_nbclust(bisScale[,1:7], kmeans, k.max = 22, method = "gap_stat")
wss <- fviz_nbclust(bisScale[,1:7], kmeans, k.max = 22, method = "wss")
silh <- fviz_nbclust(bisScale[,1:7], kmeans, k.max = 22, method = "silhouette")

wss / silh / gapStat
```

Use k-means clustering to form two most favorable clusters

```
kmBison <- kmeans(bisScale[,1:7], centers = 2)
bisClust <- bisScale
clustVec <- kmBison$cluster
bisClust <- as.data.frame(bisClust)
bisClust$cluster = clustVec
bisClust$cluster <- as.factor(bisClust$cluster)

ClusteredPCA <- PCA(bisClust[,1:7])
```

```
clusterCoords <-ClusteredPCA$ind$coord
clusterCoords <- as.data.frame(coords)
clusterCoords <- clusterCoords[,-c(6,7)]
clusterCoords$cluster <- bisClust$cluster
clusterCoords$Cluster <- as.factor(clusterCoords$cluster)

hull_cluster <- clusterCoords %>%
  group_by(cluster) %>%
  slice(chull(Dim.1, Dim.2))

MyClusterPlot <- ggplot(clusterCoords, aes(x = Dim.1, y = Dim.2)) + geom_point(shape = 21)

MyClusterPlot + aes(fill = cluster) + geom_polygon(data = hull_cluster, alpha = 0.5) +
  labs(x = "PC1", y = "PC2", colour = "Cluster", title = "PCA of Bison Skulls") + 
  geom_vline(xintercept = 0) + geom_hline(yintercept = 0) +
  geom_label_repel(aes(label = rownames(clusterCoords))) +
  scale_fill_manual(values = c("#619CFF", "#F8766D"))
```

```
## Ignoring unknown labels:
## • colour : "Cluster"
```

```
hull_sex$Sex <- as.factor(hull_sex$sex)
hull_cluster$Cluster <- as.factor(hull_cluster$cluster)
```

Plot purported sex and cluster on the same PCA

```
MyClusterPlot +
  geom_polygon(
    data = hull_cluster,
    aes(x = Dim.1, y = Dim.2, group = Cluster, fill = factor(Cluster)),
    alpha = 0.25,
    linewidth = 0.3,
    colour = "gray25",
    inherit.aes = FALSE) +
  scale_fill_manual(name = "Cluster", values = c("1" = "#4DB6AC", "2" = "#D4A72C")) +
  new_scale_fill() +
  geom_point(aes(fill = Sex), shape = 21, size = 3) +
  geom_label_repel(aes(label = rownames(clusterCoords), fill = Sex),colour = "black" ) +
  scale_fill_manual(values = c("?" = "#B8B8B8","F" = "#F8766D","M" = "#619CFF")) +
  labs(x = "PC1 (75.50%)", y = "PC2 (16.47%)", title = "PCA of Bison Skulls") +
  geom_vline(xintercept = 0) +
  geom_hline(yintercept = 0)
```

Perform MANOVA to see if significant PC scores are predicted by
purported sex

```
sigCols <- normPCA$ind$coord[,1:3] #only include PC's accounting for first 95% of variance
sigCols <- as.data.frame(sigCols)
sigCols$sex <- NA
bisFem <- rownames(bisonSex)[bisonSex$sex == "F"]
bisMale <- rownames(bisonSex)[bisonSex$sex == "M"]
bisNB <- rownames(bisonSex)[bisonSex$sex == "?"]
sigCols$sex <- ifelse(rownames(sigCols) %in% bisFem, "F", 
                      ifelse(rownames(sigCols) %in% bisMale, "M", "?"))
bisPCAMan <- manova(cbind(Dim.1, Dim.2, Dim.3) ~ sex, data = sigCols)
summary(bisPCAMan)
```

```
##           Df  Pillai approx F num Df den Df Pr(>F)  
## sex        2 0.58785   2.6364      6     38 0.0309 *
## Residuals 20                                        
## ---
## Signif. codes:  0 '***' 0.001 '**' 0.01 '*' 0.05 '.' 0.1 ' ' 1
```

```
eta_squared(bisPCAMan, partial = TRUE)
```

```
## # Effect Size for ANOVA (Type I)
## 
## Parameter | Eta2 (partial) |       95% CI
## -----------------------------------------
## sex       |           0.29 | [0.02, 1.00]
## 
## - One-sided CIs: upper bound fixed at [1.00].
```

Lastly, estimate the degree of dimorphism in each metric using finite
mixture analysis and the method of means

```
estimateTable <- matrix(nrow = 7, ncol = 2)
rownames(estimateTable) <- colnames(bisonAdults)
colnames(estimateTable) <- c("FMA", "MM")
estimateTable <- as.data.frame(estimateTable)

pearsonTable <- read.csv("pearsonTable.csv")

for (i in 1:nrow(estimateTable)) {
  metric <- na.omit(bisonAdults[,i])
  mean <- mean(metric)
  OR <- max(metric) - min(metric)
  n <- length(metric)
  k <- pearsonTable$stDevsInRange[pearsonTable$SampleSize == n]
  sigmaTot <- OR/k
  expSub <- k*(2^.5) #expected number of subpop st devs in total OR
  twoSubPercent <- 2/expSub #portion of OR expected to be occupied by 2 sigmaSub
  oneSubPercent <- twoSubPercent/2 #portion of OR expected to be occupied by 1 sigmaSub
  distanceToSub <- oneSubPercent*OR #numeric value of one sigmaSub <- distance from overall mean to mean of each subpop
  bigMean <- mean + distanceToSub
  smallMean <- mean - distanceToSub
  fmaEstimate <- bigMean/smallMean
  estimateTable$FMA[i] <- fmaEstimate
  bigs <- metric[metric > mean(metric)]
  bigMean <- mean(bigs)
  smalls <- metric[metric < mean(metric)]
  smallMean <- mean(smalls)
  mmEstimate <- bigMean/smallMean
  estimateTable$MM[i] <- mmEstimate
}
```
